# MeadoWatch: a long-term community-science database of wildflower phenology in Mount Rainier National Park

**DOI:** 10.1038/s41597-022-01206-8

**Published:** 2022-04-01

**Authors:** Rubén D. Manzanedo, Aji John, Meera L. Sethi, Elli J. Theobald, Berry Brosi, Joshua Jenkins, Ava Kloss-Schmidt, Emilia Lia, Annie Schiffer, Jordana Sevigny, Anna Wilson, Yonit Yogev, Janneke Hille Ris Lambers

**Affiliations:** 1 Plant Ecology, Institute of Integrative Biology, D-USYS, ETH Zürich, Zürich, Switzerland; 2 Biology Department, University of Washington, Seattle, USA; 3 National Park Service, Washington, USA

**Keywords:** Biodiversity, Population dynamics, Community ecology, Grassland ecology

## Abstract

We present a long-term and high-resolution phenological dataset from 17 wildflower species collected in Mt. Rainier National Park, as part of the MeadoWatch (MW) community science project. Since 2013, 457 unique volunteers and scientists have gathered data on the timing of four key reproductive phenophases (budding, flowering, fruiting, and seeding) in 28 plots over two elevational gradients alongside popular park trails. Trained volunteers (87.2%) and University of  Washington scientists (12.8%) collected data 3–9 times/week during the growing season, using a standardized method. Taxonomic assessments were highly consistent between scientists and volunteers, with high accuracy and specificity across phenophases and species. Sensitivity, on the other hand, was lower than accuracy and specificity, suggesting that a few species might be challenging to reliably identify in community-science projects. Up to date, the MW database includes 42,000+ individual phenological observations from 17 species, between 2013 and 2019. However, MW is a living dataset that will be updated through continued contributions by volunteers, and made available for its use by the wider ecological community.

## Abstract (summary)

**Table:** 

Measurement(s)	Timing of four key reproductive phenophases of wildflowers
Technology Type(s)	Field observations

## Background

Increasing temperatures linked to anthropogenic climate change have caused and are expected to continue causing major phenological shifts (i.e., changes in the timing of relevant biological events) of many ecosystems. Higher early season temperatures, changing precipitation patterns, and increasing severity or frequency of extreme weather events have already significantly affected the timing of migration and reproduction across taxa^1–3^. These profound phenological changes have reportedly even affected large-scale pheno-patterns, such as homogenizing the leaf-out timing across tree species in the Alps^4^.

Phenology is increasingly recognized as a key indicator of the impact of climate change on natural ecosystems^1^. However, the complexity and species-specific nature of phenological responses requires the use of long-term, multi-species, and high-resolution data to provide assessments of the response to environmental change both at the individual and whole-community level (e.g.^5,6^). An example of how long-term, open phenological data can contribute to our understanding of ecological change is the Rocky Mountain Biological Laboratory (RMBL) phenology dataset. RMBL researchers have recorded flowering phenology of 135 plant species every other day (recently changed to 3 times per week) since 1973 (DOI:10.17605/OSF.IO/JT4N5). RMBL resolution and scale have allowed researchers to account for factors such as long-term and metric definition variability when studying shifts in community phenology^7^, and interactions between species^8^, in ways that would have been impossible without this long-term dataset. Other large-scale initiatives, such as the National Phenological Network (https://usanpn.org) have expanded the taxonomical and spatial scales of phenological assessments and engaged tens of thousands of volunteers across the United States diverse ecosystems, producing 143 peer-reviewed publications to date. Increasing the number and specially the availability of long-term phenological datasets is a remarkable scientific investment to improve our understanding of climate change effects on ecological communities.

However, a challenge to implement and maintain long-term phenological assessments of many species is their costs (monetary and time) (but see e.g.^9–11^). Community-science approaches (also known as citizen-science, public participation in scientific research, or crowd-sourced data) could prove to be a particularly useful tool for acquiring phenological data with remarkable resolution and scale (e.g.^12^), while simultaneously boosting public participation in science^13^. Furthermore, the increase in scale need not come at the cost of lower data quality, as trained volunteers have consistently shown high accuracy and reliability in locating and determining species and phenophases^14–16^. While community-sourced data have its challenges (such as inconsistent temporal resolution^17^) and requirements for data quality and consistency; it is clear that community science opens a cost-effective and engaging way to enhance traditional scientific approaches^18,19^.

**Fig. 1:**
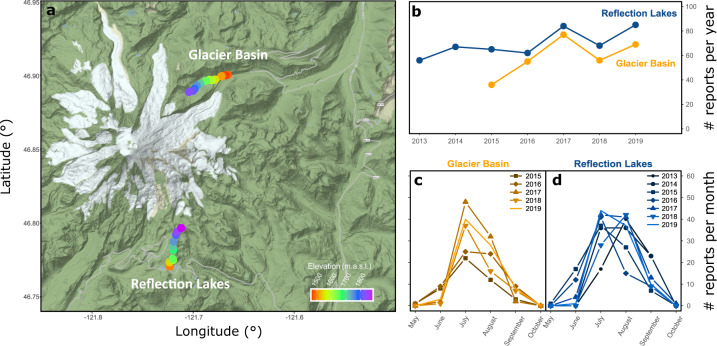
Location of MW transects and temporal replication of MW data. (**a**) Transects are located along two popular hiking trails (Glacier Basin and Reflection Lakes). Individual plots are shown as dots, colored by elevation. (**b**) Total number of trail reports per year for each MW transect. (**c,d**) Monthly trip report replication per trail and year.

Here, we provide access to a long-term and high-resolution phenological dataset of 17 wildflower species, from one of the most iconic ecosystems in North America: the alpine and subalpine meadows of Mt. Rainier National Park (named Mount Tahoma by the local Salish and Puyallup people, Fig. 1), collected within the MeadoWatch (MW) project (http://www.meadowatch.org/). MW includes data collected by volunteers and scientists on wildflower phenology, soil temperature, and snow coverage on 28 permanent plots located along two elevational transects. Data from the seasons 2020 and 2021 have been (or are being) collected and are in process of data cleaning and preparation. The link provided here will be regularly updated to grant access to the most recent data.

## Methods

### Study origin and design

**Fig. 2:**
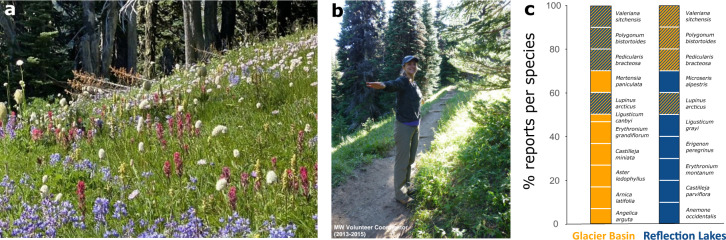
Alpine meadows, plot extension, and target species. (**a**) Species-rich alpine meadow in Mount Rainier National Park (Mount Tahoma), showing many of the target species in the foreground. (**b**) MW volunteer coordinator Anna Wilson at a plot, indicating the arm span that defines the plot area (personal likeness used with confirmed consent). (**c**) Species composition and proportion of reports per species in each of the transects; species common to both trails are highlighted with striped shadowing. Photographs: A. John (**a**), L. Felker (**b**).

The MeadoWatch project (MW) is a project run collaboratively between the University of Washington (UW) and the United States National Park Service to monitor the phenology of alpine and subalpine wildflower species across large elevational gradients in Mount Rainier National Park (Fig. 2). MW was established in 2013 with the goal of understanding long-term effects of climate change on Mount Rainier National Park wildflower communities using community-science approaches. The first MW transect was established along Reflection Lakes, Skyline, and Paradise Glacier trail system in 2013 (9–11 plots). In 2015, MW expanded to include a second transect (15–17 plots) along the Glacier Basin trail (Fig. 1a). The MW transects span around 5 km each, over a 400 m altitudinal gradient (Reflection Lakes: 1490m–1889m a.s.l.; Glacier Basin: 1460m–1831m a.s.l.)

Plots are located along the side of each trail, marked with a colored survey marker. The area of each plot is defined by the arm-span of volunteers when they position themselves over the plot marker looking away from the trail (Fig. 2b). While less accurate than marking the corners of plots, this approach was used to avoid establishing permanent structures in wilderness areas within the National Park. The surveyed area in each plot is, on average, 1.25 m^2^. Each plot is also equipped with temperature sensors (HOBO Pendant Logger, Onset Computer Corp.) buried approximately 4 cm below the ground. Sensors are placed at the start of each fall season and removed at the beginning of each summer season for data downloading. The HOBO sensors provide an estimate for the date of snow disappearance and *in-situ* temperature at 3 hours intervals. Once plots are covered in snow, soil temperatures remain at 0 °C and show no diurnal variation, so that daily changes in temperatures above 1 °C can be used to determine the earliest date without snow cover^20^. We use these approaches to provide dates of snow appearance and disappearance, snow cover duration, and minimum soil temperatures for each year and plot. Occasionally, temperature data during the snow disappearing window were lost due to sensor failure or loss of sensors (which occurs because plots are not permanently marked and/or well-meaning visitors remove sensors). This, and the lack of temperature sensors in the first year of the project, resulted in approx. 20% of cases of missing data. In those cases, we used a data imputation method to estimate the missing values based on data from nearby plots and a parallel temperature data collection with 890 total observations. These estimates were highly reliable in filling the data gaps (see Appendix C in^16^ for further details).

### Focal species

We originally targeted 16 native wildflower species along each transect, which were chosen based on their abundance, ease of identification, and presence in the plot. Four of those target species were present on both transects. In 2016 we replaced one species with a different one (see further information below), making for a total of 17 species monitored (Fig. 2c). The focal species are: American bistort* (*Polygonum bistortoides*), avalanche lily (*Erythronium montanum*), bracted lousewort* (*Pedicularis bracteosa*), broadleaf arnica (*Arnica latifolia*), cascade aster (*Aster ledophyllus*; synonym *Eucephalus ledophyllus*), glacier lily (*Erythronium grandiflorum*), Gray’s lovage (*Ligusticum grayi*), magenta paintbrush (*Castilleja parviflora*), mountain daisy (*Erigenon peregrinus*; synonym *Erigeron glacialis*), northern microseris (*Microseris alpestris*; synonym *Nothocalais alpestris*), scarlet paintbrush (*Castilleja miniata*), sharptooth angelica (*Angelica arguta*), sitka valerian* (*Valeriana sitchensis*), subalpine lupine* (*Lupinus arcticus;* synonym *Lupinus latifolius* var. *subalpinus*), tall bluebell (*Mertensia paniculata*), Canby’s licorice-root (*Ligusticum canbyi*), and western anemone (*Anemone occidentalis*). Asterisks denote species monitored along both trails.

Due to challenges in species identification, we dropped Canby’s licorice-root (*Ligusticum canbyi)* as a target species in 2016. Consequently, *Ligusticum canbyi* has limited replication in the database (Fig. 2c). While we included the phenological records of this species for the sake of completeness, we recommend focusing on the other 16 species, which are both better represented (in terms of data coverage) and are free of any potential misidentification issues.

For additional information on the species, methods, identification cues, and image resources see: http://www.meadowatch.org, https://www.youtube.com/channel/UCGBFTKxf8FIWswMDxBavpuQ, and the appendices therein^16^.

### Data collection and volunteer training

During the summer months, MW volunteers and scientists collect reproductive phenology data with a frequency between 3 and 9 trail reports per week. Each report records the presence or absence of 4 phenophases for each target species present in each of the plots. The phenophases are ‘*budding’, ‘flowering’, ‘ripening fruit’*, and ‘*releasing seed’*. Phenophases were defined as follows:

#### Budding

The beginning growth of the flower which has not yet opened. A plant is considered budding if buds are present, but the stamen and pistils are not yet visible and available to pollinators.

#### Flowering

The generally “showy” part of the plant that holds the reproductive parts (stamens and pistils). A plant is considered flowering when at least one flower is open, and the stamens and pistils are visible and available for pollination and reproduction.

#### Ripening fruit

The fruit develops from the female part of the flower following successful pollination. In the target species, fruits can take many forms, from hard, fleshy capsules, juicy berries, to a feathery tuft on the end of a seed. A plant is in the ripening fruit stage when reproductive parts on at least one reproductive stalk are non-functional and the formation of the fruit part is clearly ongoing (visible), but seeds are not yet fully mature and not yet being dispersed.

#### Releasing seed

After the fruit ripens, seeds are released to be dispersed by gravity, wind, or animals. A plant is considered in the releasing seed stage if seeds are actively being released on at least one reproductive stalk (but there are still seeds present).

A full description, including illustrations for each species’ phenophase and identification cues is available in http://www.meadowatch.org/volunteer-resources.html, as well as in Annex 1 - Supplementary Documentation. Multiple phenophases can be present simultaneously, depending on the species, and are noted independently. Additionally, volunteers are also asked to record the presence of snow (*‘snow covered plot’, ‘partially covered plot’*, or *‘snow-free plot’*), and, since 2017, the presence of damage by herbivory (*‘presence of browsed stems’*) on each plot.

In years not impacted by the SARS-Cov-2 pandemic MW volunteers attend an in-person 3-hour botanical and phenological training session taught by UW scientists at the beginning of each sampling season. Volunteers were also provided with detailed species-identification guides, including an extensive description of sampling methods and location of the plots. The trainings for the 2020 and 2021 seasons were held virtually via a series of online training videos. In these years, volunteers took a quiz on wildflower phenology, plant identification and data collection methods after viewing these videos and were required to ‘pass’ a certain threshold to volunteer (unlimited attempts were allowed). During these virtual trainings, volunteers were provided with digital copies of the species identification guides, with many returning volunteers using printed guides they had kept from previous years.

At the end of their phenological hike, volunteers submit their data sheets either by depositing them in lockboxes located within the park, or by scanning and emailing them directly to mwatch@uw.edu. Data are then entered manually and stored in the UW repositories after being checked for consistency at the end of each sampling season.

The parallel data collection by members of UW’s Hille Ris Lambers group (including PI, postdoctoral researchers, graduate students, and trained interns) acted as the following: (i) a quality-control, i.e., allowing us to compare the consistency in phenology assessments between volunteers and scientists, and (ii) a way to increase the temporal resolution and scale of the data, e.g., by reducing early season gaps and ‘weekend bias’^17^. This parallel expert sampling was carried out around once a week between 2013 and 2020, showing great consistency between the two groups. For detailed comparisons between volunteers and scientists’ assessments see the data validation section (as well as Appendix E in^16^).

### Processed data

In addition to the raw phenological data, we also provide here parameters to construct the year, species, and plot-specific flowering phenology based on the timing of snow disappearance (as in^16^). Models describe unimodal probability distributions that were fitted with maximum likelihood models to binomial flowering data from each species, year, and plot. These curves have been used to estimate peak flowering dates and diversity and link them to reported visitor experiences^16^. Here, we provide the 3 parameters defining the unimodal curve of flowering probability per species *i*, plot *j* and year *k*: the duration of flowering (𝛿_*ijk*_), the maximum probability of flowering (𝜇_*ijk*_), and peak flowering (in DOY - ρ_*ijk*_); following the equations described in^16^ and https://github.com/ajijohn/MeadoWatch).

The parameters of these probability distribution curves are ready-to-use values that can be broadly and easily used to estimate floral compositional change over past seasons due to changing environmental conditions—for example, to inform plant-pollinator interaction networks if combined with pollinator behavioral data (e.g.^21^).

## Data Records

MW data are stored permanently in the repository: 10.5061/dryad.g1jwstqs2^22^, as well as privately in UW’s private repositories, to ensure public availability and future safety of the data.

When MW is updated, files will be updated and named accordingly in both repositories. The DOI for MW data, however, will remain unchanged and constantly updated using version control.

MW dataset consists of 6 files in csv format:

1. **MW_metadata.xlsx**: this file contains information on each variable, including variable name, description, units, file, reference for methods. It also includes the equivalency between 4-letter species code used in the reports and the full botanical name.
2. **MW_SiteInfo_2013_2020.csv**: this file contains the identification and location of each MW plot::
  - Site_Loc. Plot id.
  - Location. Plot geographical coordinates. Latitude, longitude. WGS84.
  - Elevation. Plot elevation. Meters above sea level. Directly measured in the field.
  - Forest type. Plot is located on Forest, Low meadows, or High meadows.
3. **MW_Phenodat_2013_2019.csv**: this file contains raw phenological records:
  - Transect: Name of the trail where the plot is located: Glacier Basin or Reflection Lakes.
  - Date: in DD/MM/YYYY format.
  - Observer 1–6: Author(s) of the phenology assessment. Names have been anonymized to a unique number string for privacy purposes.
  - Observer_group: variable determining if the observation is made by one individual (‘single’), two individuals (‘pair’) or three or more individuals (‘group’).
  - Scientist_or_volunteer: variable determining if the assessment is done by a volunteer or a professional scientist. Groups that contained at least one scientist were labeled as ‘Scientist’.
  - Site code: joint site number and transect id (e.g., GB11 = plot #11 in Glacier Basin trail).
  - Species: wildflower species identified by its 4-letter species code (e.g., CAPA = Castilleja parviflora). See equivalency of 4-letter code and full botanical name in the MW_metadata.xlsx file.
  - Snow: presence of snow on the plot, where 0 = ‘snow free plot’; 0.5 = ‘partially covered plot’; and 1 = ‘snow-covered plot’.
  - Bud: presence (1) or absence (0) of budding phenophase of the species in the plot.
  - Bud_rank: each phenophase for each species at each plot was also ranked based on relative abundance, from 1 (most common phenophase) to 4 (least common phenophase) observed. These measurements occurred from 2013 to 2015 but were dropped in subsequent assessments (replaced with NA in the database subsequently).
  - Flower: presence (1) or absence (0) of flowering phenophase of the species in the plot.
  - Flower_rank: similar to ‘Bud_rank’ (see above but focused on flowering phenophases).
  - Fruit: presence (1) or absence (0) of ripening fruit phenophase of the species in the plot.
  - Fruit_rank: similar to ‘Bud_rank’ (see above but focused on fruiting phenophases).
  - Disperse: presence (1) or absence (0) of releasing seed phenophase of the species in the plot.
  - Disperse_rank: similar to ‘Bud_rank’ (see above but focused on seeding phenophases).
  - Herb: whether damage by herbivory was present (1) or not present (0). Whenever volunteers did not fill up this, we assigned a NA value. However, since all volunteers are asked to assess herbivory, these NA are functionally equivalent to the non-presence (0).
4. **MW_SDDall.csv:** temperature and snow condition data per plot and year.
  - Year, Transect, Site_Num, Site_Code, Site_Loc: are consistent with those in MW_SiteInfo_2013_2020.csv and MW_Phenodat_2013_2019.csv.
  - Calibration: Calibration parameter for PredSDD, if required.
  - Snow_appearance_date: earliest day in the year with consistent snow cover in MM/DD/YYYY.
  - Snow_disappearance_date: latest day in the year with consistent snow cover in MM/DD/YYYY.
  - Snow_cover_duration: number of days with consistent snow cover over the plot.
  - Minimum_soil_temp: minimum recorded temperature recorded in the plot, in °C.
  - SDD: Date of snow disappearance, in day of the year (DOY).
  - Notes: Comments on the SDD estimation.
  - predSDD: Predicted snow disappearance date using a linear model that included a combination of site location (code) and year. For more information on this model see^16^.
5. **MW_Phenocurves.csv**: this file contains the derived phenological probability curves per species and plot used in^16^, which can also be accessed in the on-line platform, which are defined per species, year and plot using the following variables:
  - Year, transect, site_code, plot, SDD, and species: consistent with the nomenclature used in previous documents.
  - Peak: date (in DOY) for the maximum flowering probability ρ_*ijk*_.
  - Duration: Duration of flowering (𝛿_*ijk*_) parameter.
  - Maximum: Maximum probability of flowering (*μ*_*ijk*_) parameter.
6. **MW_Volunteer_info_2013_2019.csv:** this file contains information for the MW volunteers, including:

- Observer: anonymized ID of the MW volunteer

- First_participation: year of first participation of the volunteer as author of a datasheet

- Year_of_first_training: year of first recorded participation of the volunteer in the training courses organized by MW.

- Training_type: whether the observer has been trained in-person (noted as ‘Traditional’) or only on-line (noted as ‘Online’). Observers that were attended online trainings after attending in-person ones were considered as having a traditional training.

## Technical Validation

### Temporal resolution

Phenological reports span the growing season (typically between May and October), but have consistently good temporal resolution between June and September (Fig. 1c,d). The average time between reports across the whole growing season was variable from year to year but was consistently between 3 and 5 assessments per week for each of the trails (Table 1).

**Table 1:** Temporal resolution, as the average number of days between reports during the growing season, for each year and trail in the MW dataset. For this table, the growing season is defined by the first and last report for each.

Year	Mean time between reports (days)	
	*Glacier Basin*	*Reflection Lakes*
2013	—	*1.35*
2014	—	*1.29*
2015	*2.88*	*1.92*
2016	*2.12*	*1.91*
2017	*1.82*	*1.57*
2018	*2.20*	*1.56*
2019	*1.69*	*1.63*

### Comparison of scientist and volunteer phenological assessments

**Fig. 3:**
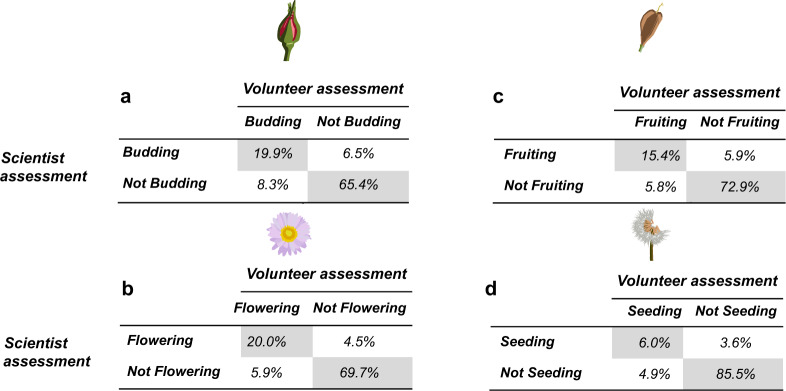
Confusion matrices comparing scientists (rows) and volunteers (columns) assessments of four wildflowering phenophases. Values represent the percentage of assessment across paired scientist-volunteer assessments. For example, Budding-Budding in (**a**) would represent the percentage of total reports in which both scientists and volunteers concluded a particular species to be budding). Agreeing assessments (i.e., true positives and true negatives), are highlighted in grey. The addition of these agreement assessment percentages (e.g., in a: 19.9% + 65.4% = 85.3% accuracy) represent the mean phenostate accuracy across trails as shown in Fig. 4a.

To estimate the consistency in phenological assessments between scientists and volunteers, we compared scientist and volunteer reports collected on the same day and trail (Fig. 3). After pairing the reports, we followed a confusion matrix approach; where ‘true positives’ and ‘true negatives’ would be instances of agreement between scientist and volunteers, respectively; ‘false positives’ would be instances when scientists recorded absence of a phenophase and volunteers recorded presence; and ‘false negatives’ are the opposite case (volunteer reported presence while scientist recorded absence). We are aware that many of our volunteers are highly botanically trained (even more than some of our scientists!), therefore this terminology and approach has the sole objective of quantifying the agreement or disagreements in species and phenophases ID.

Using this framework, we calculated the accuracy, sensitivity, and specificity metrics for each trail, phenophase, and species as follows:

1 $$Accuracy=\frac{SY.CY+SN.CN}{Total}=\frac{Scientist-Volunteer\;agreements}{Total}$$

2 $$Sensitivity=\frac{SY.CY}{SY.SY+SY.CN}=\frac{True\;Positives}{True\;Positives+False\;Negatives}$$

3 $$Specificity=\frac{SN.CN}{SN.CN+SN.CY}=\frac{True\;Negatives}{True\;Negatives+False\;Positives}$$

Where *‘SY.CY’* indicate true positives (scientist and volunteer agree on presence), ‘*SN.CN*’ are true negatives (both agree on absence), ‘*SY.CN*’ are false negatives (scientists fills in ‘presence’ while volunteer interprets it as ‘absent’), and ‘*SN.CY*’ are false positives (scientist considers a phenophase absent while volunteer reports it as present).

Therefore, within our study:

- accuracy represents the percentage of consistent assessments (either positives or negatives);

- sensitivity indicates the chance for a phenological observation to be only captured by volunteers, but not by professional scientists;

- and specificity the chance for a phenological observation to be only captured by a scientist, but not by a volunteer.

**Fig. 4:**
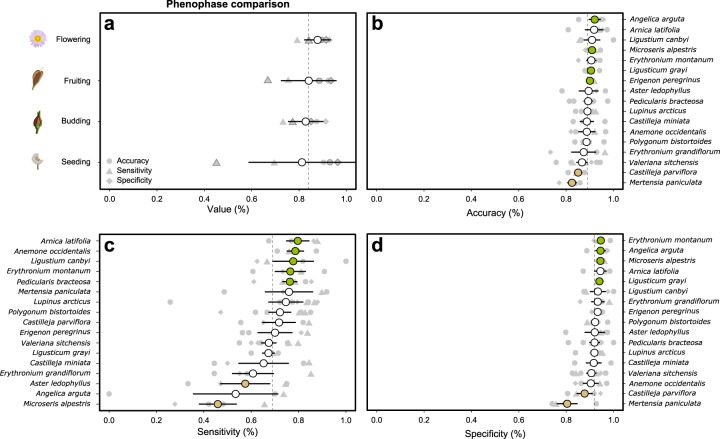
Agreement between scientists and volunteers in the estimation of different phenophases (**a**) and across species (**b,c,d**). Mean value per phenophase or species are shown for each group. Accuracy (**b**) represents the percentage of consistent assessments (either positives or negatives), (i.e., 80% accuracy would indicate that 80% of reports show agreement between scientists and volunteers). Sensitivity (**c**) represents the chance for a phenological observation to be captured only by volunteers but not by scientists (i.e., 80% sensitivity would indicate that 20% of reports by volunteers missed that species). Finally, specificity (**d**) represents the chances for a phenological observation to be captured only by scientists but not by volunteers (i.e., 80% specificity would indicate that 20% of reports by scientists missed that species). Species are sorted in decreasing order. Averages and standard errors are shown per species. Species significantly better (green) or worse (orange) than the across species average (vertical dotted line) are highlighted.

We found consistently high accuracy for the assessments of all phenophases (>82%) and species (>73%, Figs. 3, 4). As expected, the flowering phenophases showed the highest accuracy, specificity, and sensitivity. However, differences between phenophases were not significant (Fig. 4a). Similarly, differences between transects were small and not consistent. While accuracy and specificity were consistently high across all species (Fig. 4b,d), sensitivity was lower and more variable among species (Fig. 4c). Sensitivity was particularly low for the seeding and fruiting phenophases, indicating a higher chance for volunteers to miss the fruiting or seeding bodies of our target species. By species, *Mertensia paniculata* and *Castilleja parviflora* had the lowest accuracy and specificity. This may be caused by their smaller size and the more subtle transition between similar-looking phenophases (e.g., fruiting and releasing seeds stages in *Castilleja parviflora*). Note, however that in both cases accuracies and specificities are still >80% for both species (Fig. 4b,d). The species with lowest sensitivity (<60%) were *Microseris alpestris, Angelica arguta*, and *Aster ledophyllus*, which were particularly challenging to detect for volunteers in their seeding state (Fig. 4c).

All the analyses were performed in R^23^. Graphs were exported from R to Inkscape Software (https://inkscape.org/) to produce the final published figures.

### Data exploration tool

**Fig. 5:**
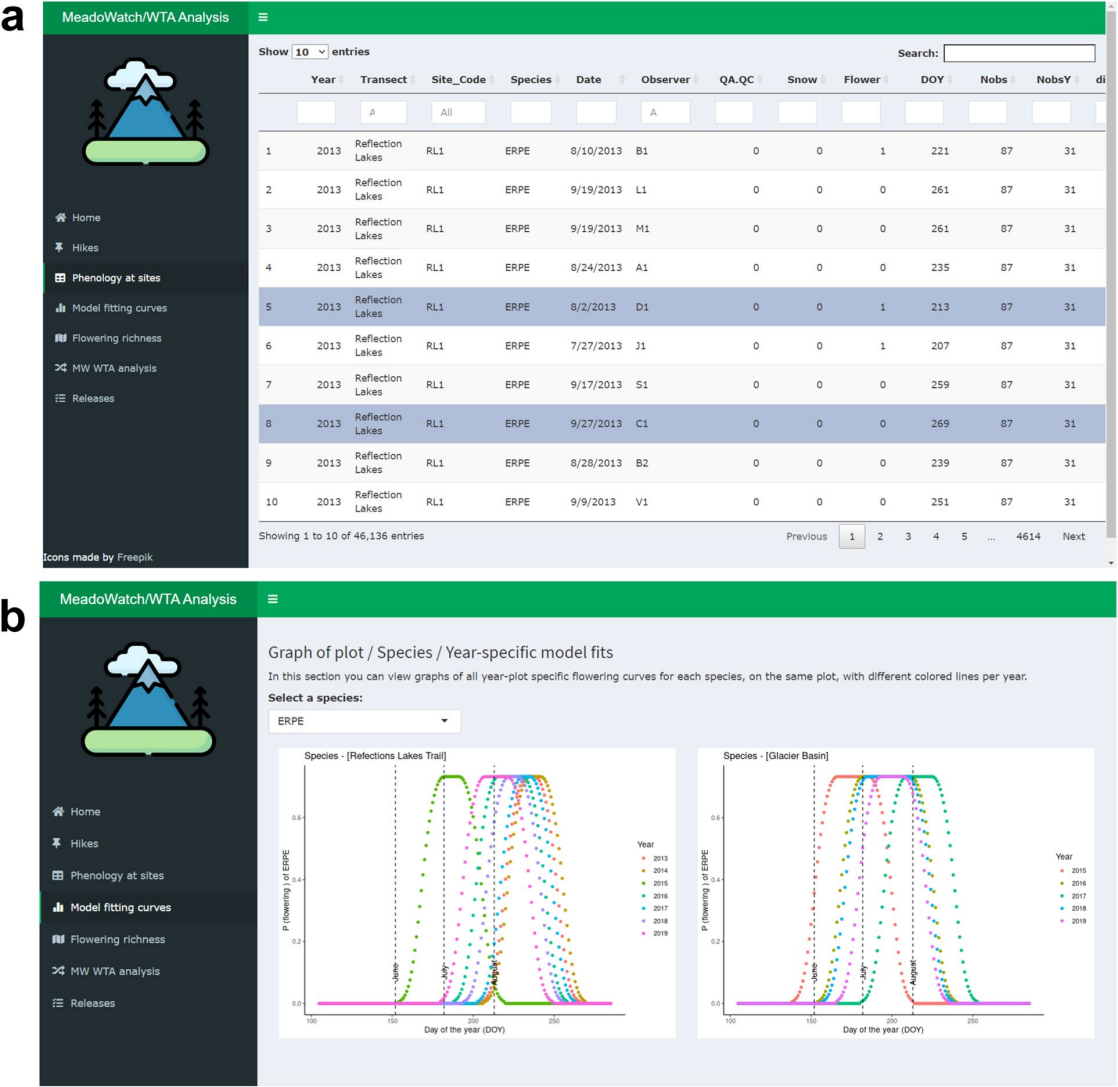
Screen captures of the interface of the data exploration tool (https://explorations.shinyapps.io/MeadoWatch/). The raw data exploration menu and search options (**a**) and multi-year curves of flowering probability for Erigenon peregrinus (ERPE) (**b**) are shown. The code to run the data exploration tool locally is also available in the MW GitHub repository (https://github.com/rdmanzanedo/MeadoWatch_SDATA_2022.git).

To interactively showcase the possibilities of the collected data and allow users to easily explore the data, we developed a Shiny App^24^. Shiny App is an interactive web application framework written in R^23^ that aims at creating cross-platform web experiences with customizable user-interface (UI) components. Shiny Apps can be launched via any locally installed R environment or can be hosted on the shinnyapps webserver (shinyapps.io). The data exploration tool is accessible at https://explorations.shinyapps.io/MeadoWatch/. Using dropdown menus, users can search and explore the raw data (‘*Phenology at sites*’ menu, Fig. 5a), the species-specific phenological curves (‘*Model fitting curves*’, Fig. 5b), the number of flowering species for each day of the year in each of the projects year (‘*Flowering richness*’), and the connection between flower phenology and hiker satisfaction reported in^16^ (‘*MW WTA analysis*’). Data releases and improving functionalities will be progressively implemented to this application and announced on the GitHub and ‘Releases’ section of the web.

## Usage Notes

### Data availability and use

This paper represents MW’s first data release, which will be updated regularly via the provided links. MW data is freely available to use for any non-commercial purposes by citing this reference or the DOI to the database^22^. Commercial use of the database requires express authorization by the MW coordinator (Prof. Dr. Hille Ris Lambers). For privacy, the identities of MW volunteers in the database have been anonymized.

### Applications

MW data can be used to explore changes in wildflower community phenology, species climate sensitivity, and could be combined with other datasets, such as pollinator data, to study shifts in trophic interactions in North American natural ecosystems. The database has been used to date to study the reliability of wildflower phenology estimates using traditional and community science methods^25^, to show social-ecological mismatches in changing phenology^12^, as ‘ground truth’ to fit models that detect montane phenology from satellite imagery^26^, and to explore the effects of warming climate on flowering diversity and timing and how it affects the experience of visitors to National Parks^16^. We also provide information on volunteer identity (anonymized) and training type, which can be used to evaluate methods characteristics of community-science volunteers. Further information on MW volunteers may be available upon private request to the authors. We hope that by making these data openly and easily accessible, multidisciplinary works and models can help better understand phenological changes in natural ecosystems.

## Supplementary information

Annex 1 - Supplementary Documentation.

## Data Availability

The most up-to-date data will be available and updated regularly at: 10.5061/dryad.g1jwstqs2. The code to replicate all the analyses, as well as the raw data finalized as of August 2021 is openly available via: https://github.com/rdmanzanedo/MeadoWatch_SDATA_2022.git. The code to launch the Shiny app data exploration tool is available in the same GitHub folder and as a browser application here: https://explorations.shinyapps.io/MeadoWatch/.
