# Supplementary material for: MeadoWatch: a long-term community-science database of wildflower phenology in Mount Rainier National Park
Source: Sci Data. 2022 Apr 1;9:151. doi: 10.1038/s41597-022-01206-8 (PMC8976009; doi:10.1038/s41597-022-01206-8)
Supplement: Supplementary file 1 — Annex 1 - Supplementary Documentation. [file 41597_2022_1206_MOESM1_ESM.pdf]

MEADOWATCH

A Citizen Science Project
Exploring Wildflower Phenology
At Mount Rainier National Park

Glacier Basin

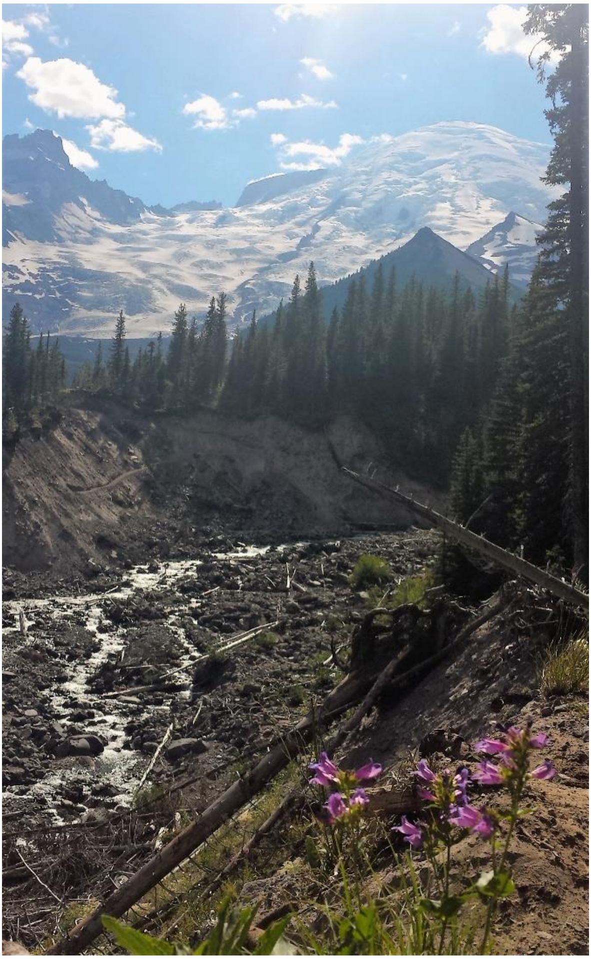

www.meadowatch.org

Background

The goal of **MeadoWatch** is to collect high quality data on the seasonal timing of flowering (i.e. wildflower phenology) in the high mountain meadows of Mt. Rainier. Scientists need this kind of data to gain an understanding of the biological impacts of climate change, and we cannot do it without your help! As volunteers, you will help us by monitoring the phenology (see picture below for different 'phenophases') of several plant species over a large gradient in elevation (and climate). This information helps us understand how climate change might alter wildflower phenology, and eventually, could aid resource managers at Mt. Rainier National Park prepare for climate change. By participating, you get the chance to engage in scientific research, and (we hope) gain an understanding of the potential impacts of climate change. Thank you for your help!

Phenology is the study of the timing of recurring life stages (e.g. budding, flowering, fruiting, and releasing seeds) – in other words, the biological manifestation of the seasons. Phenology is frequently cued by climatic variables (e.g. snow disappearance, temperature), and thus, will likely be sensitive to climate change. **Phenophases** are stages of phenology, as illustrated below by Subalpine Lupine.

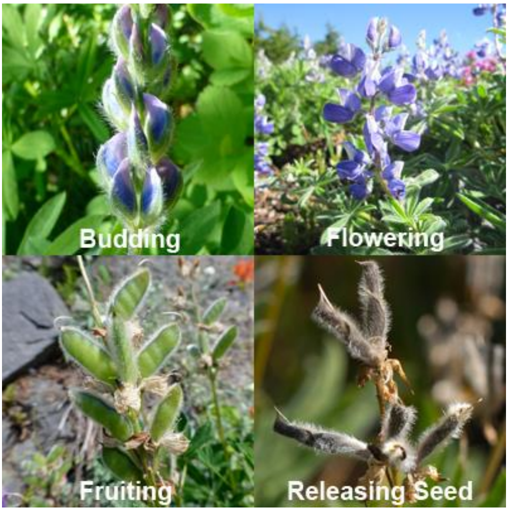

Directions to the Glacier Basin Hike

To help us collect data, you will: **1)** Hike along the Glacier Basin Trail to reach the seventeen plots (detailed directions on pages 4-20) **2)** identify the phenophases present and absent for ten focal species (descriptions and photos on pages 23-42) **3)** record what you see on a data sheet (which you should have with you) **4)** then return your data sheet to the brown metal box at the trailhead of the Glacier Basin Trail (attached to the map display) or mail it back to us.

The seventeen plots are located along the Glacier Basin Trail (6.2 miles roundtrip, 1600' elevation gain). To start your hike, park at the climber and picnicker parking lot in the White River campground, and walk through the D Loop of the campground. Your hike will climb approximately 1,600 feet (~500 meters) of elevation. Our directions to each plot are written assuming you will be collecting data uphill.

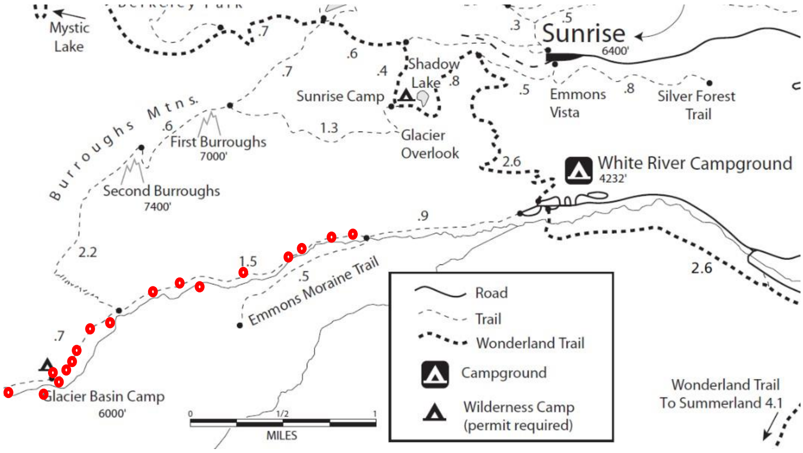

Finding the Plots

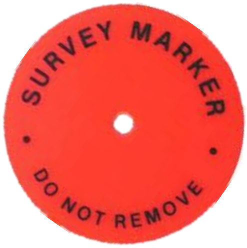

Use the directions and photos within the following pages to find the seventeen plots. The location of the plot will also be marked by an orange or yellow plastic survey washer located in the trail (see picture right). However, these survey markers might not be present early in the season, although you may see flagging marking our buried microclimate sensors. They may also get covered in trail dust or well-meaning visitors may remove them (we will check this and regularly replace any missing markers). We also provide GPS coordinates (if you have your own unit – WSG 84 datum), but you should be able to find the plots without a GPS unit. Directions to some plots describe streams or small drainages, which may be dry in late summer (but the ditch where the stream once ran will still be visible).

Once at the location, stand with the survey washer between your feet, facing in the direction explained in the plot-specific directions. You will be observing the phenology of our focal species in a rectangular plot made with your arm span as the length of the plot and one arms' length as the width of the plot (see below).

Please help maintain this beautiful natural resource by <u>staying on the trail at all times</u> (you will never need to leave the trail to collect data)

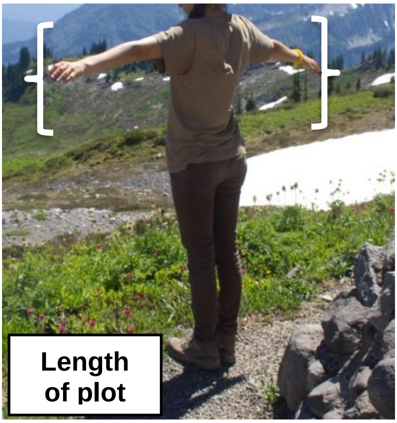

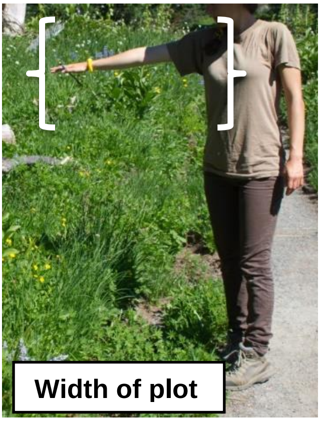

Plot 1

Plot 1 is ~150 feet past the turn off to the Emmons Moraine Trail. Soon after you pass that sign, there will be a small clearing on the right followed by a small stream that you will cross (which might be seasonally dry). A fir tree is on the left edge of the trail just before crossing the stream. Plot 1 is on your right, just past this small stream (when you are facing plot 1, your right fingertips will be at the edge of the small stream).

GPS: 46.90000 lat 121.66604 long
Elevation: 1483 m

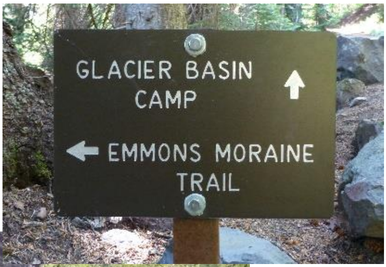

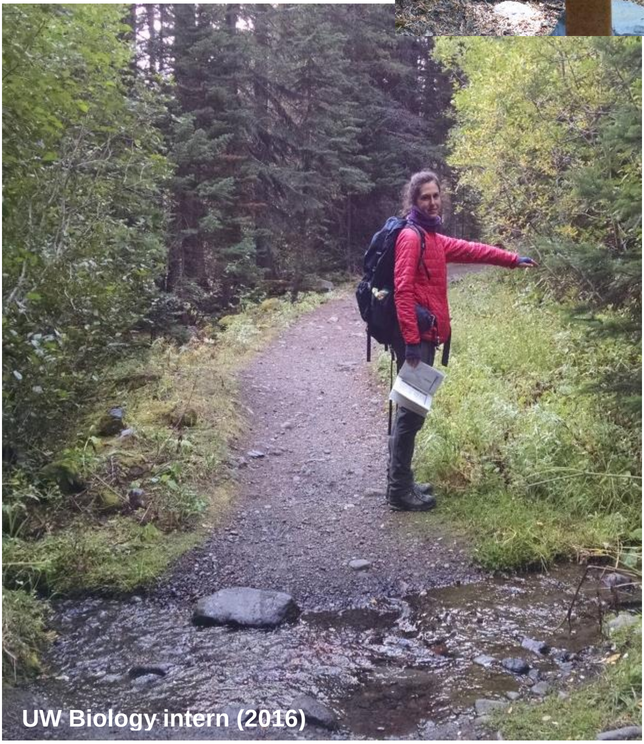

Plot 2

Continue uphill from crossing a series of 5 or 6 streams (depending on the time of year!). Just before you turn uphill on a switchback, there will be 3 largish jumbled rocks in a row on the right side of the trail. At the first rock, turn to your left and Plot 2 will be there as you face the river. Don't forget to look to your right for a beautiful view of the White River and Mt. Rainier!

GPS: 46.89985 lat 121.66798 long
Elevation: 1469 m

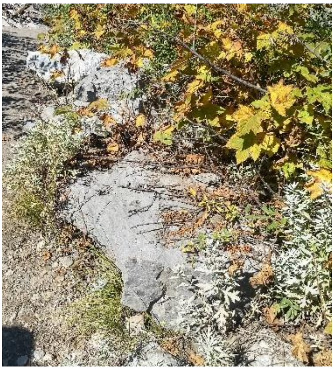

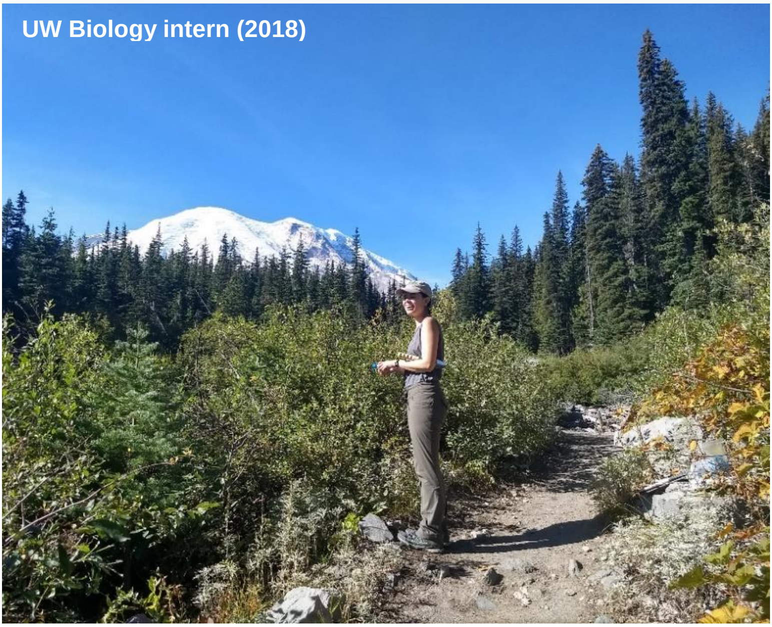

Plot 3

Continue up the trail, through a switchback, crossing a bridge, another switchback and yet another bridge. After the bridge, continue through a large clearing caused by a rockslide, and the trail will wind back into the forest, into a second smaller clearing with lots of moss and other vegetation. You will cross two small streams, after which you will encounter Plot 3 is on the right of trail (90 feet past the last small stream, just after a large flat rock that is also on the right). Directly behind you (as you face the plot) there will be many stumps, logs and debris.

GPS: 46.89950 lat 121.67163 long
Elevation: 1500 m

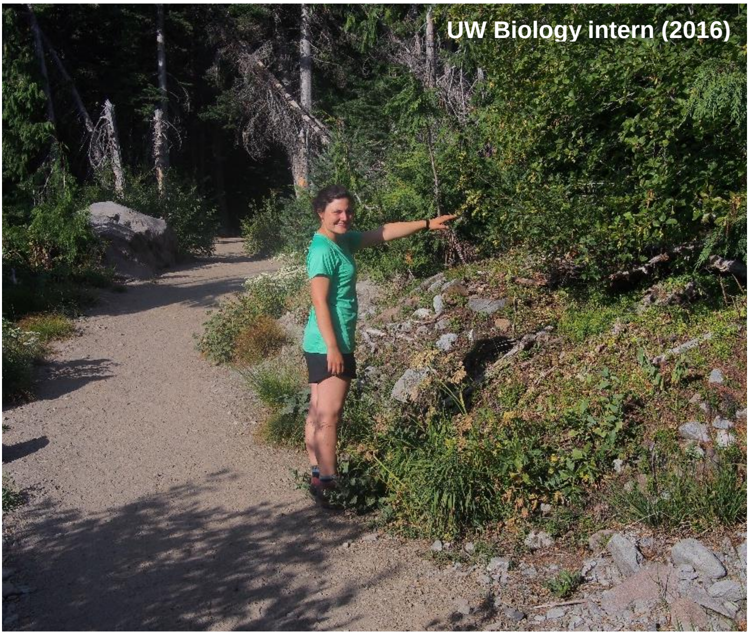

Plot 4

Continue hiking for about 1/4th mile. You will come into the forest ~1/4 mile, with many roots crossing the trail in the forest. Pop out into a clearing with a view of Rainier, and the trail will hug the bank to the right. Plot 4 is on the right, about 7-8 steps after the end of the wooden bridge over the stream. There is an erosion control beam on the opposite side of the trail from Plot 4 (behind you). If you pass metal mining equipment, you have gone too far.

GPS: 46.89704 lat 121.67803 long
Elevation: 1547 m

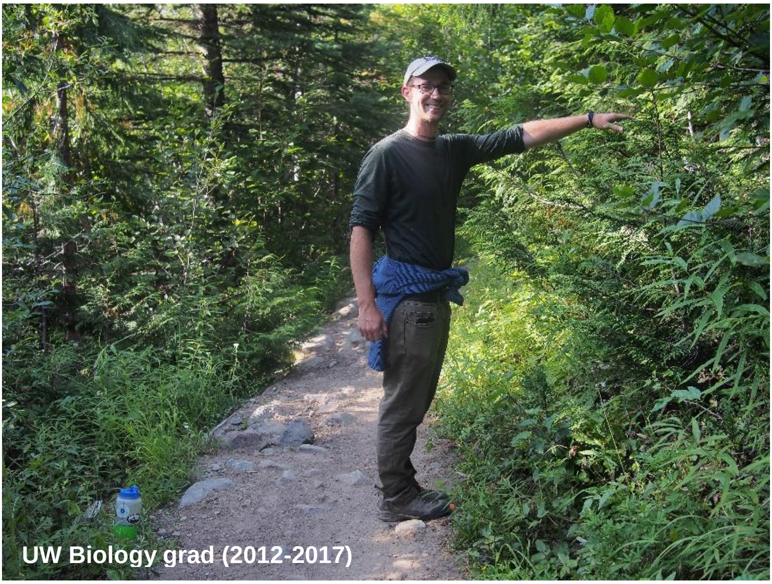
UW Biology grad (2012-2017)

Plot 5

Keep heading uphill at a moderate incline until you pass through 2 large rocks on either side of the trail. Plot 5 is on the right of the trail ~25 feet beyond those rocks. There will be a downed tree stuck in the alders above you at Plot 5.

GPS: 46.89661 lat 121.68150 long
Elevation: 1613 m

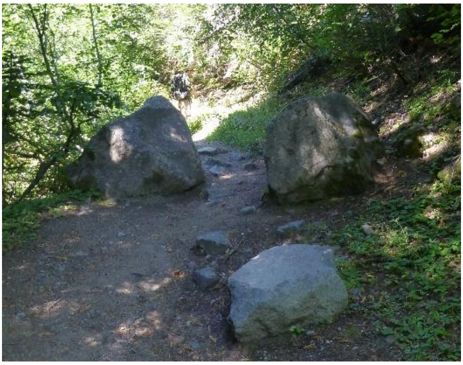

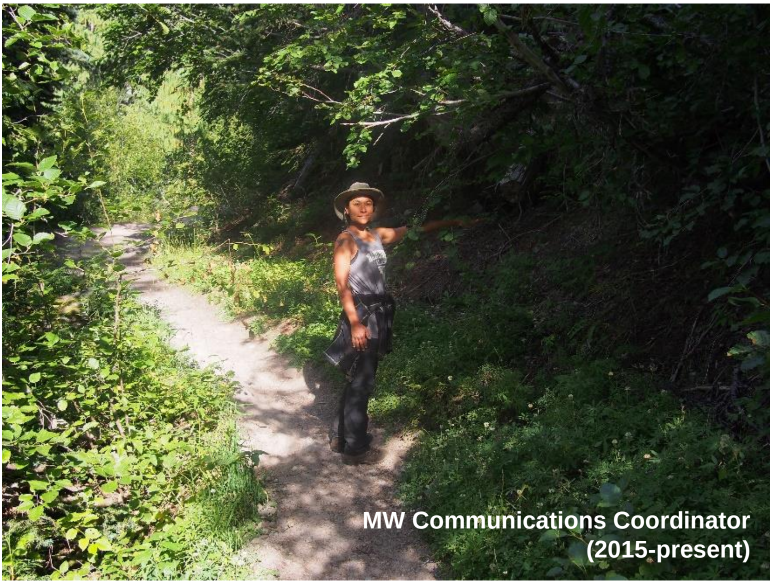

Plot 6

Continue hiking uphill from plot 5 (Plot 6 is very near plot 5). Just after you cross a small stream / gully (dry by the end of the season), and before you get to a large boulder in the trail that is on the right you will find plot 6 on the right of the trail.

GPS: 46.89685 lat 121.68232 long
Elevation: 1582 m

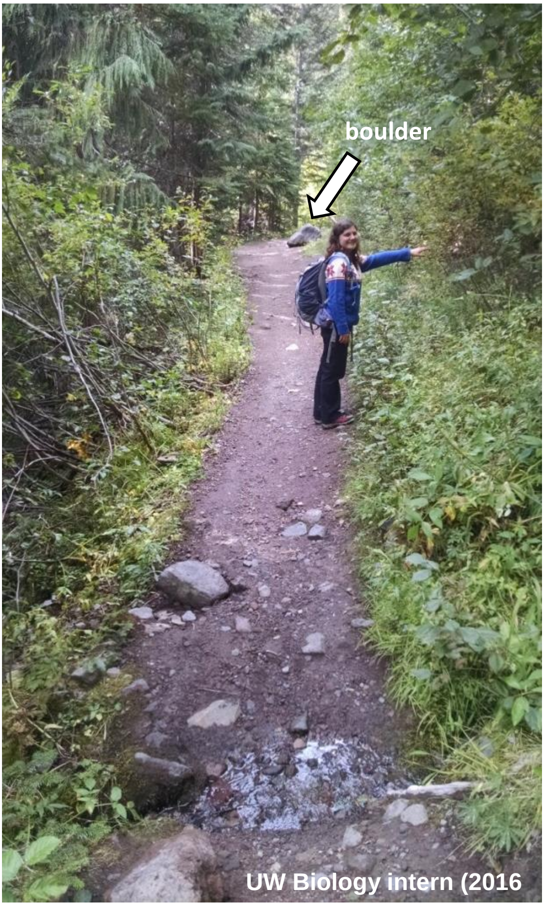

Plot 7

Continue hiking uphill, passing three large boulders on your right. You will pass through small clearings with views of Mt. Rainier, and duck back into the forest. Just after the second of two additional large boulders (about 20 feet apart), also on the right side of the trail, you will find plot 7.

GPS: 46.89693 lat 121.68362 long
Elevation: 1600 m

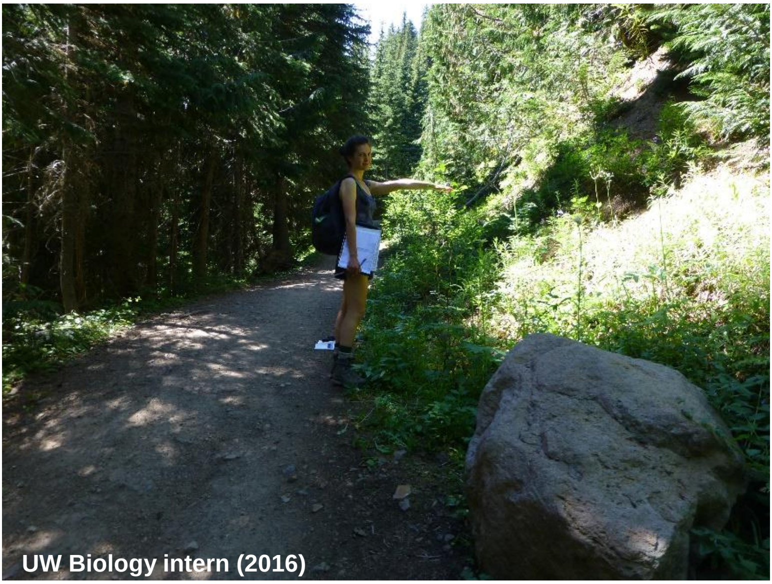
UW Biology intern (2016)

Plot 8

Continue hiking uphill. You will cross a bridge with a large piece of rusty pipe in a stream followed by a boulder. Continue past several openings where you can see a landslide on the left across the stream. You will also pass a boulder on the left and a hillslide on the right with several downed trees. After passing through another opening (created by a recent avalanche), you will pass another boulder on the left, until you see many dead trees and stumps on the right in front of you. Stop ~40ft before this opening, and plot 8 will be on the left, about 10 feet past a 3-trunked fir tree. Your right arm will reach towards a two trunked fir tree.

GPS: 46.89612 lat 121.68729 long
Elevation: 1647 m

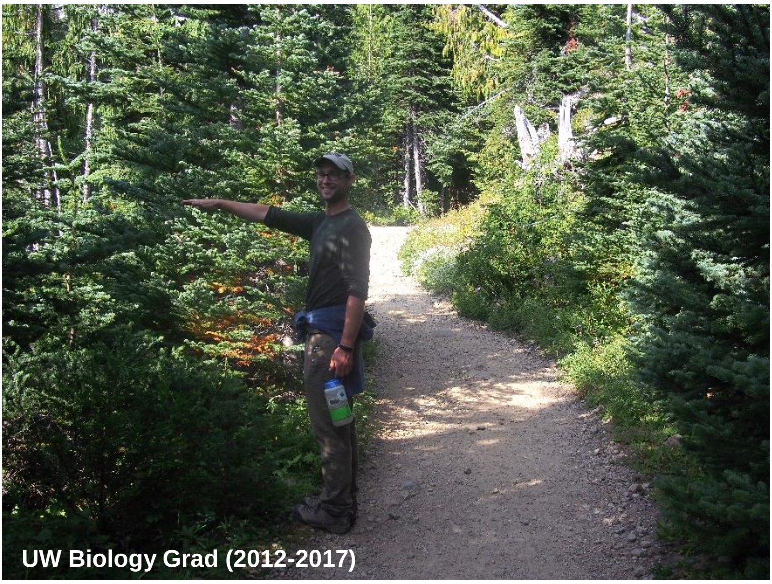

UW Biology Grad (2012-2017)

Plot 9

Continue uphill and cross 2 bridges. Proceed about 180ft past the turnoff to Sunrise (5 miles). Plot 9 will be on the left, just past a fir tree with large gall at waist height which has a smaller chest high fir in front of it. Stand facing Plot 9 (on the left) with your left hand reaching towards the small fir tree.

GPS: 46.89431 lat 121.69288 long
Elevation: 1713 m

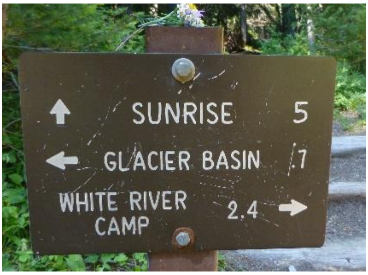

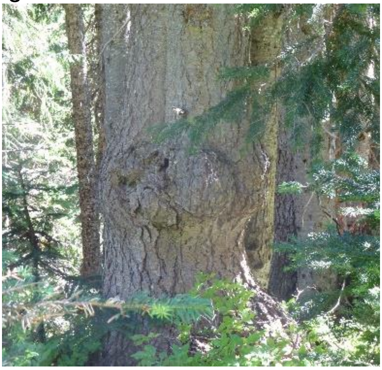

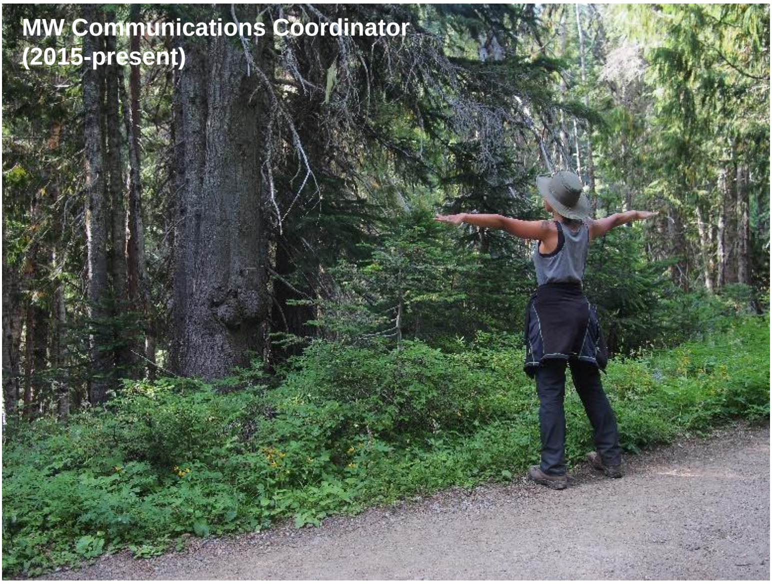

Plot 10

Continue hiking. You will cross 2 pipes and 2 bridges. Pass a sign near a switch back directing you to Glacier Basin. Ascend into a small meadow and keep climbing uphill. Stop right before a set of stairs that curve to the left. Plot 10 will be on the right where the trail widens near a two trunked fir tree.

GPS: 46.89297 lat
121.69640 long
Elevation: 1748m

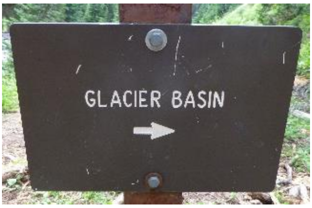

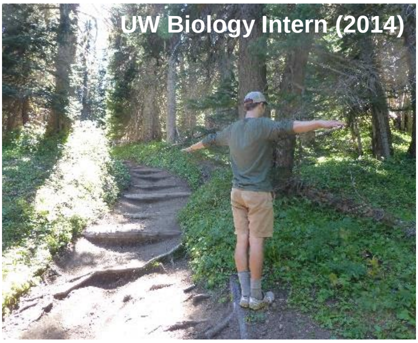

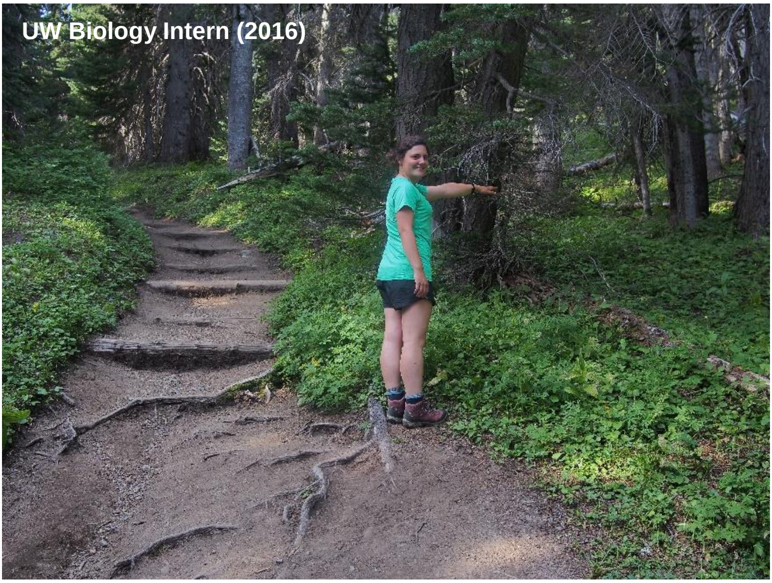

Plot 11

Follow the stairs uphill and continue to climb through meadows. You will ascend another set of stairs. Cross a bridge and continue until you pass between 2 trees that are very close together, like a tree tunnel! Plot 11 will be on your right about 10 ft. past the tree tunnel.

GPS: 46.89089 lat 121.69839 long
Elevation: 1766m

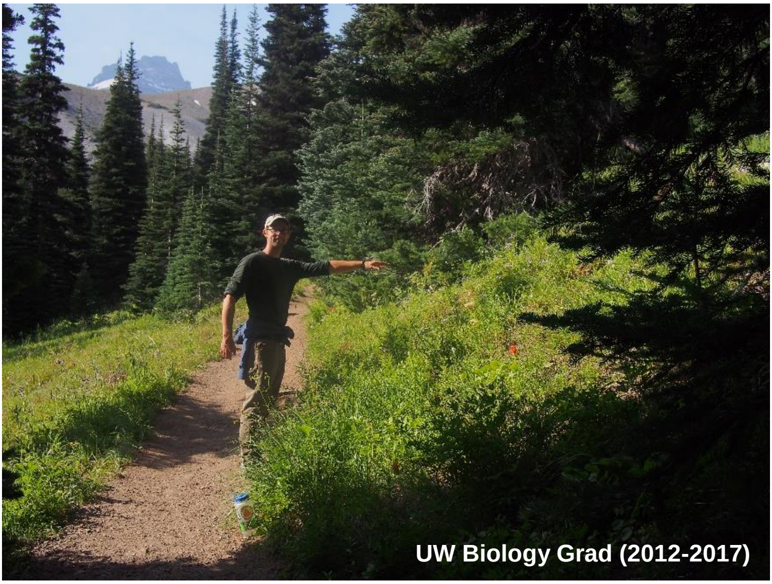
UW Biology Grad (2012-2017)

Plot 12

The trail narrows and you will ascend 8 stairs to Plot 12 (about 500 feet from plot 11, in the same meadow), which is on the left, just before a diagonally angled stair step.

GPS: 46.89051 lat 121.69870 long
Elevation: 1776m

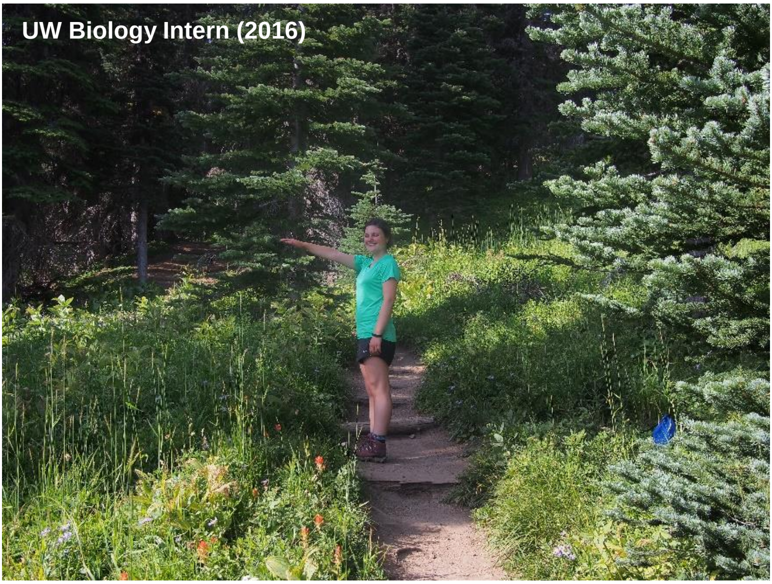

Plot 13

Plot 13 is about 200 feet past plot 12. Ascend a stairway through a small patch of trees into a meadow. Climb 4 stairs, pass between 2 large trees on either side of the trail (not quite opposite to each other). Plot 13 will be approximately 5 steps after these trees on the right.

GPS: 46.88998 lat 121.69927 long
Elevation: 1775m

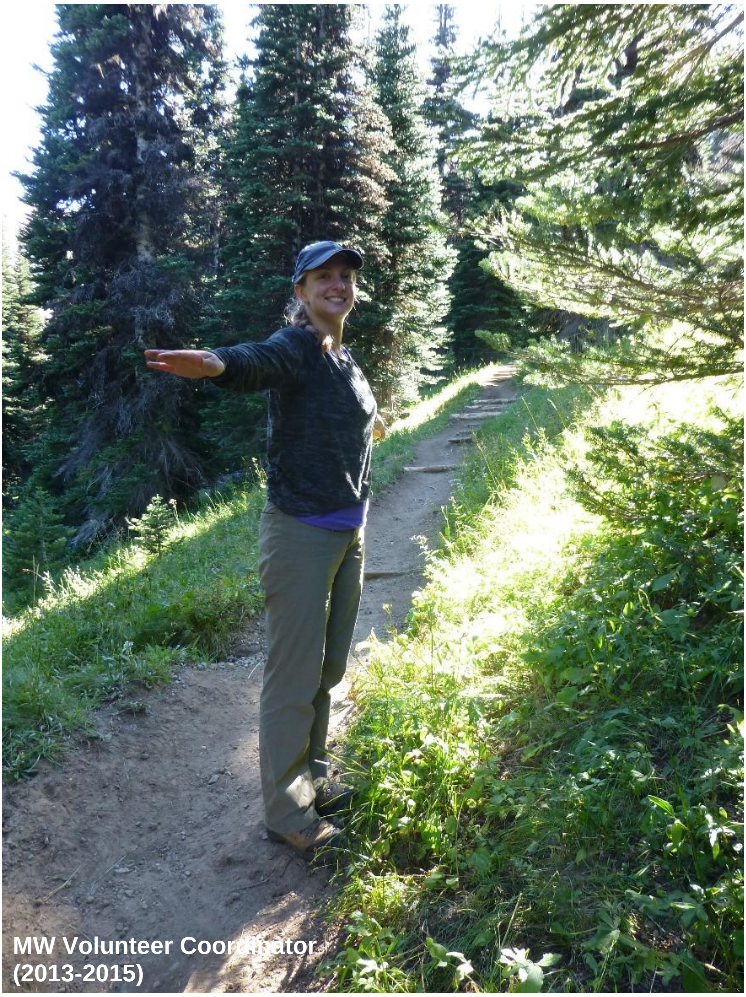

Plot 14

You will climb about 60 stairs until you reach a meadow and the trail flattens. At the end of the meadow, there is a short tree on the left side of the trail immediately before a patch of bigger trees. You will turn to your right and align your left hand with the tree to find Plot 14.

GPS: 46.88932 lat 121.70019 long
Elevation: 1804m

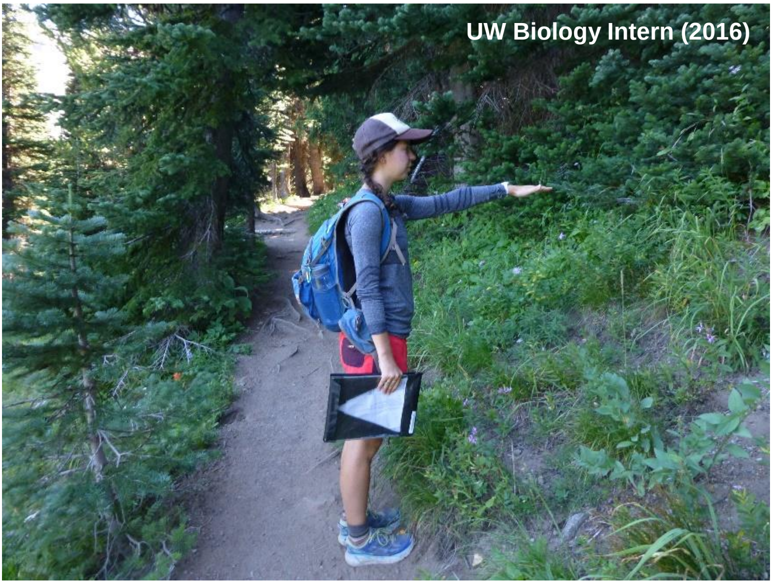

Plot 15

Shortly after leaving plot 14, and just before you reach Glacier Basin Campsite, you will find a trail heading to the pit toilet to the right with a sign. Take a short detour down that trail, and veer right at the Y-intersection with the second toilet sign (i.e. head towards the pit toilet). About 100 feet beyond that Y intersection, at a small clearing (to the left) is plot 15, on your left. You should be passing a dead fallen tree that is about 1 ft in diameter on the right. Plot is located on the left where there are many roots in the path and a 3-trunked fir tree is directly on the right of the trail (it will be behind you when standing at the plot). BTW, Plot 15 is well out of eyesight from the toilet, so don't worry about disturbing anyone's privacy!

GPS: 46.88959 lat 121.70039 long
Elevation: 1823 m

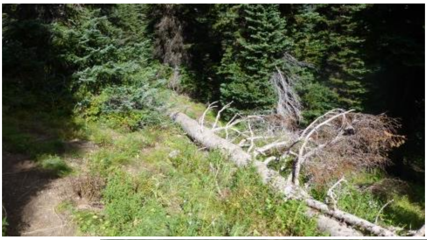

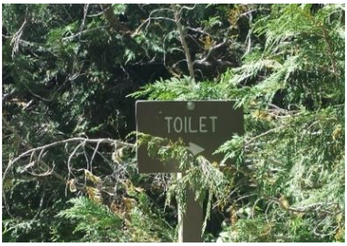

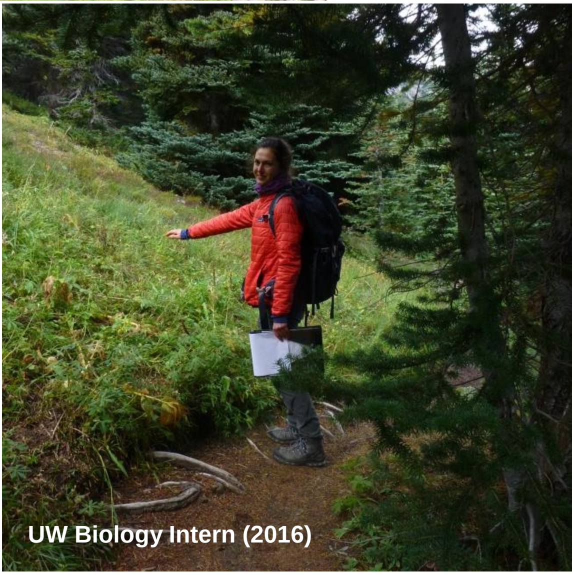

Plot 16

After censusing plot 15, go back to the main trail and hike into the campground. Just past the “End of Maintained Trail” sign you will walk out of the trees and into Glacier Basin. Don’t forget to look at the stunning views! Just after you pass through a clump of willows at the beginning of Glacier Basin, take a left on the side trail to the river / a lookout. Just at the end of the willows (about 20 feet from the mail trail) is plot 16, to your left.

GPS: 46.88874 lat 121.70200 long
Elevation: 1810 m

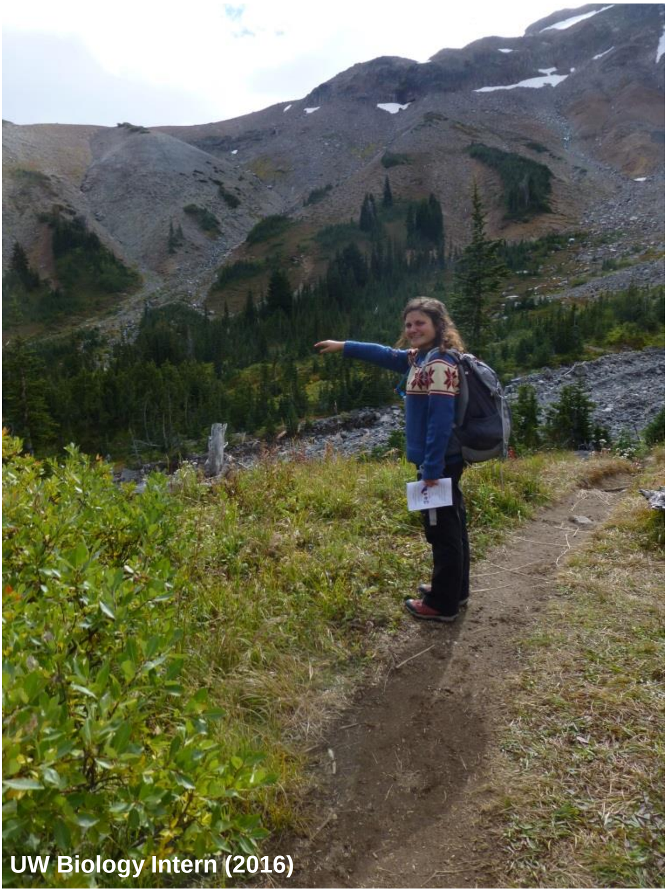

Plot 17

Return to the main trail and continue hiking through Glacier Basin. Be careful not to trip on the metal pipes running across the trail. You will pass a very large boulder in the meadow to the left, and the trail will come close to the river as you hike slightly upwards almost to the crest of the hill. You will see a medium sized rock on the left, stop 15ft before it and reach out to your right, away from the river, to find Plot 17 (near a trident shaped root in the trail). You made it to the end!

GPS: 46.88844 lat 121.70431 long
Elevation: 1831m

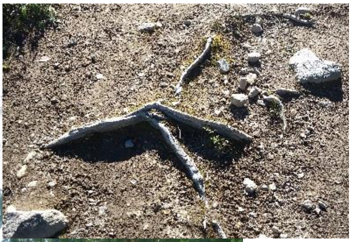

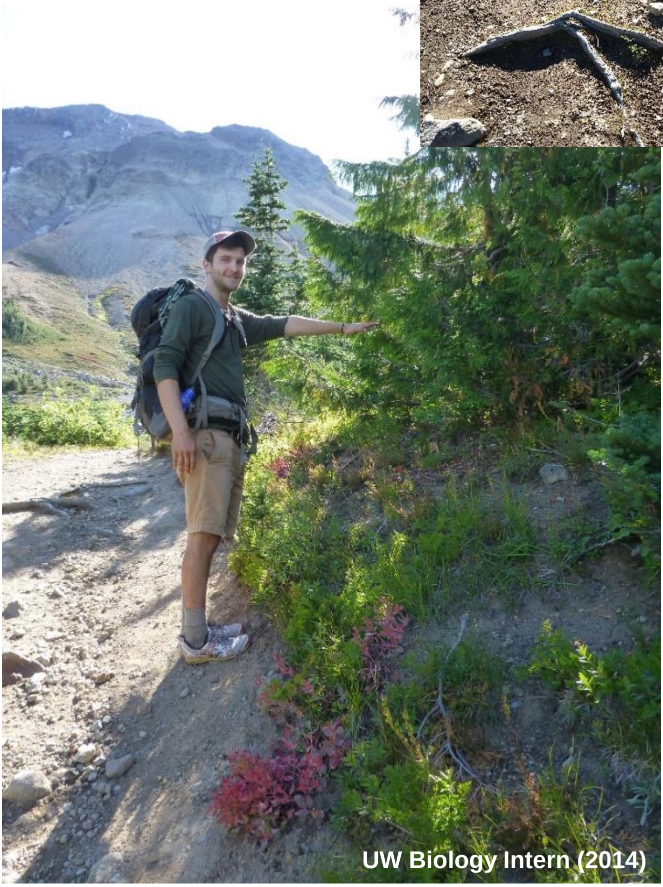

UW Biology Intern (2014)

Collecting Phenology Data

The ten focal species we chose to have you monitor are relatively abundant, represent a diversity of plant families, rely on a wide range of pollinators, and bloom at different times of the summer. While some of these species have ‘look-alikes’, most plots are located to minimize this (but see Identification Tips pages after the focal species descriptions for some exceptions).

At each plot, you will collect phenology data by noting whether the four phenophases (defined below) are present *or* absent in the plot by circling Y *or* N on your datasheet. You will often find multiple phenophases, which is fine – just note presence or absence for each phenophase. The four phenophases are a continuum of reproduction which we are trying to categorize into discrete stages, which may be hard (some may appear to be between stages). Trust your instinct and categorize the plant in the stage you think it is ‘closest’ to. If you are very unsure, note your uncertainty in the ‘notes’ section.

Wildflower phenophases:

Budding – The beginning growth of the flower. The bud will turn into a flower but hasn’t yet opened.

Flowering – Usually the showy part of the plant that holds the reproductive parts (stamens and pistils). A plant is flowering when the petals have opened, and the reproductive parts are visible and available for pollination and reproduction.

Ripening Fruit – A fruit develops from the female part of the flower. As the fruit ripens, the seeds within it develop. Fruits can take many forms, from a hard, fleshy capsule to a juicy berry to a feathery tuft on the end of a seed.

Releasing Seed – After the fruit ripens, the seeds are released to begin the next generation. Fruits can be carried away (or dispersed) by gravity, by wind, or by animals.

Note - The species description pages include a line drawing of each species, drawn by Kari Berger, followed by photos and descriptions of each phenophase (which differs by species).

Additional Data to Collect

We are asking you to collect other information that can also influence our data, and thus, our ability to understand the link between climate and wildflower phenology.

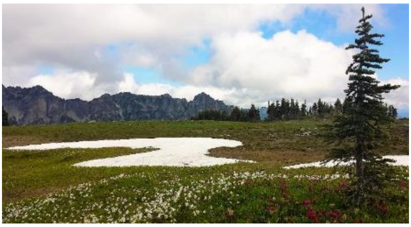

Snow: We have already found that the timing of wildflower phenology is tightly linked to the date of snowmelt. We do have microclimate sensors at each plot which help us monitor the date of snowmelt, but these sometimes fail or go missing. Please help us collect additional data by noting whether a plot is fully covered by snow (write ‘Y’ in the snow column), half covered (write ‘½‘ in the snow column), or free of snow (write ‘N’ in the snow column).

Herbivory / Damage: Many of our focal species are quite tasty to meadow mammals, including deer, elk, and marmots. Often entire stalks will disappear mid-development. If you notice that a reproductive stalk of a focal species is obviously broken, please mark a 'Y' in the herbivory column for that species. Because there could be multiple stalks per species per plot, there may still be other reproductive stalks in the plot (continue to monitor those stalks according to the phenophase(s) they display). Don't spend extra time looking for herbivory, and only note this information if it is obvious - it will be easiest to observe in species with tall stalks, like Sitka Valerian (top), Western Anemone (bottom-left), and Bracted Lousewort (bottom-right).

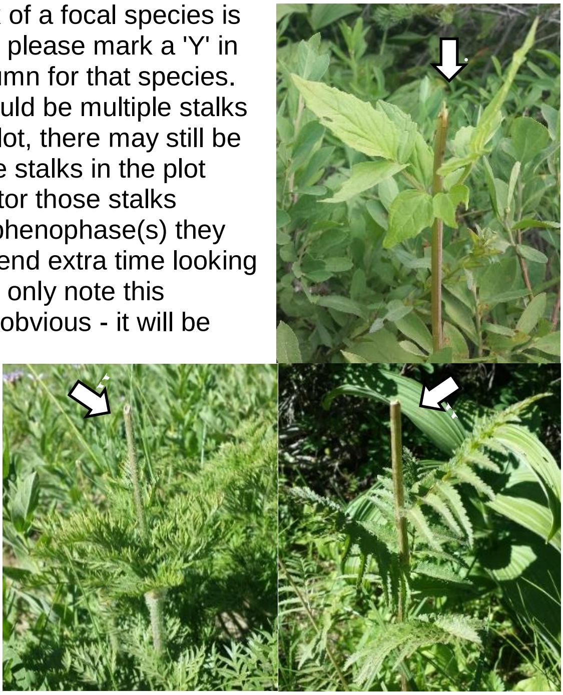

American Bistort
Polygonum bistortoides

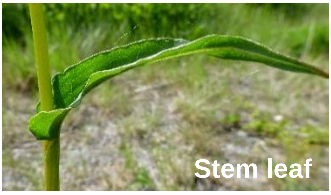

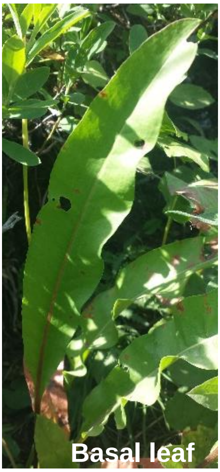

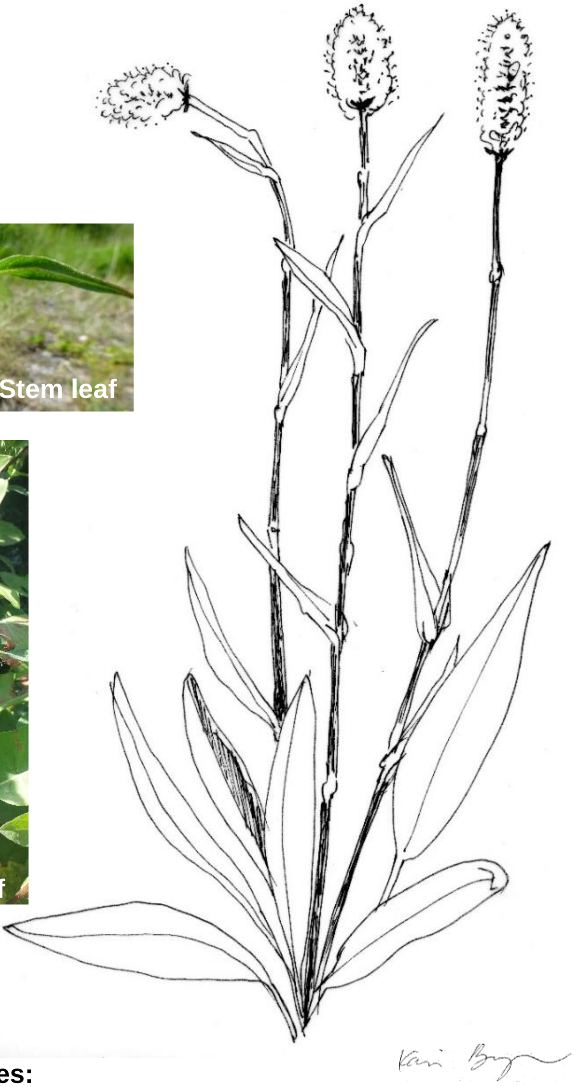

Plant and Leaves:

- Plant tall (12-24 inches), with long unbranched stem
- Leaves mostly basal with distinct petioles (stalks); oblong 4-8 inches long with distinctly light colored midrib. Stem leaves reduced with sheath attachment in place of petioles.

American Bistort

Polygonum bistortoides

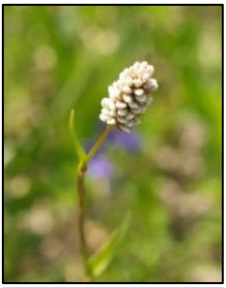

Budding:

- Many small buds cluster on top of a stem that is 1-2 feet (30-60cm)

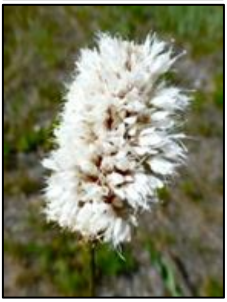

Flowering:

- Flowers are white and bristle-brush-like
- 8 stamens protrude from each blossom, looking like bristles
- The flowers smell awful (this plant is fly-pollinated and this is how it attracts pollinators)

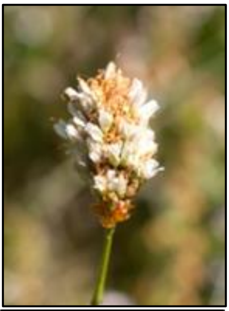

Ripening Fruit:

- The flowers stay attached to the fruits but if you were to touch them (don't!) they would feel hard
- You know the plant is fruiting when the base of each flower turns reddish

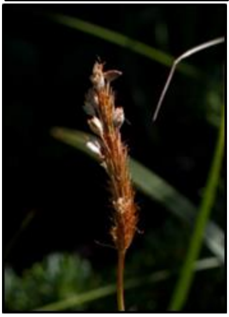

Releasing Seed:

- The white flower-looking structures fall off leaving the bare stalk
- The stalk is rusty-red and still appears hairy

Bracted Lousewort
Pedicularis bracteosa

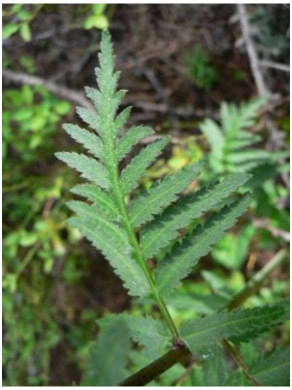

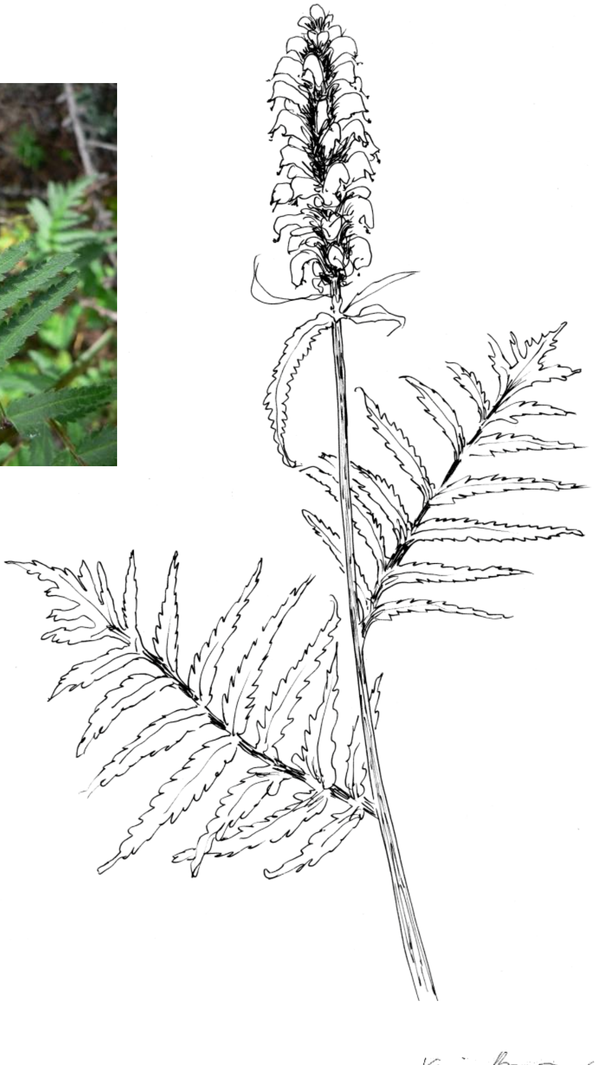

Plant and Leaves:
- Often the tallest meadow plant, reaching 3 feet (90 cm)
- Leaves are fernlike, dark green to purple

Bracted Lousewort

Pedicularis bracteosa

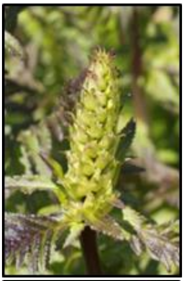

Budding:

- Buds cluster in groups of up to 40 on the tall spike-like infloresense

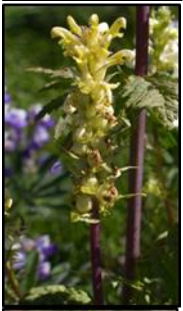

Flowering:

- Yellow flowers crowd together on a long spike on top of the stem
- If you look closely, each small flower has a hood and a lower lip – some say it looks like a parrot’s beak
- Stem and leaves are hairless but the inflorescence is typically hairy

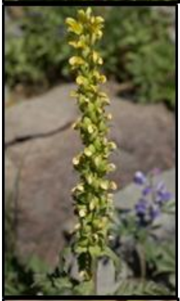

Ripening Fruit:

- The flowers turn into small, green, ball-like capsules that contain the seeds
- There may still be remnants of the flower parts left on the fruits, but the flower parts will be wilted and turning brown
- As the fruit develops the capsules turn from green to brown

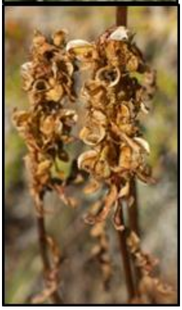

Releasing Seed:

- The fruits crack open at the top (like an urn) and release small seeds
- The stalk sounds like a rattle when the seeds are releasing because the capsules, seeds, and stems are so dry

***** NOTE ***** It is common for individual *Pedicularis* stalks to have multiple phenophases – make careful observations & be sure to consider which phenophases are present and absent.

Broadleaf Arnica

Arnica latifolia

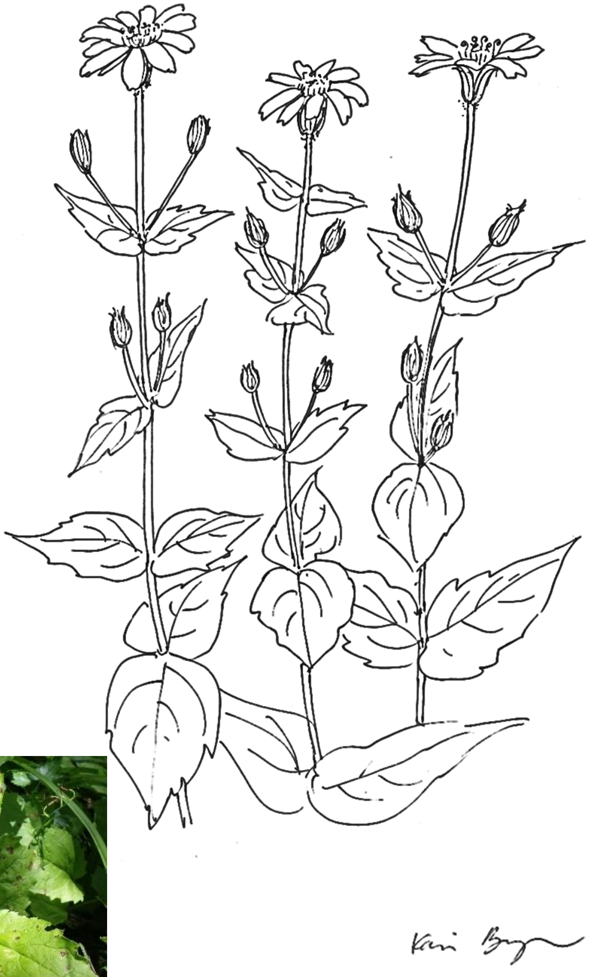

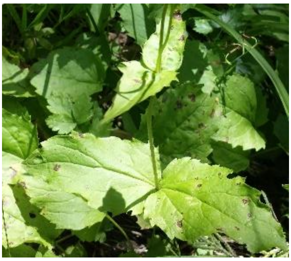

Plant and Leaves:

- Finely hairy, erect unbranched stems 4-24 in.(10-60cm) tall
- Coarsely toothed leaves opposite, rounded to elliptical. Usually 2-4 pairs per stem

Broadleaf Arnica
Arnica latifolia

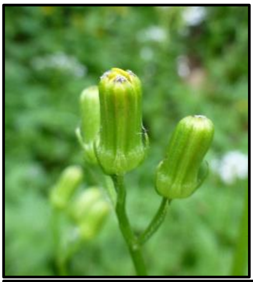

.

Budding:
- The green sepals enclose the yellow flowers

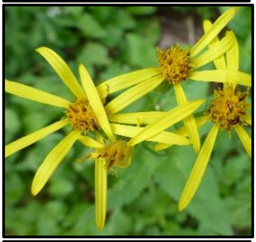

Flowering:
- 1-5 bright yellow flower heads with 8-10 ray flowers (.5-1 inch long) surrounding a central disc.
- Flower ‘petals’ are squared off and toothed at the end.

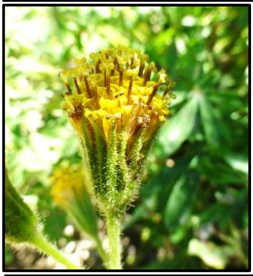

Ripening Fruit:
- Ray flowers absent or withered
- Fruits develop where disc flowers were to long white bristles, resembling a dandelion

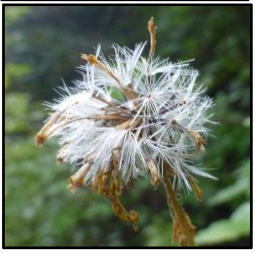

Releasing Seed:
- White bristly pappus carries the seed away in the wind

****NOTE*****Aster* and *Arnica* can look similar when in fruit and releasing seed, but *Arnica* seeds are less densely packed.

Cascade Aster

Aster ledophyllus

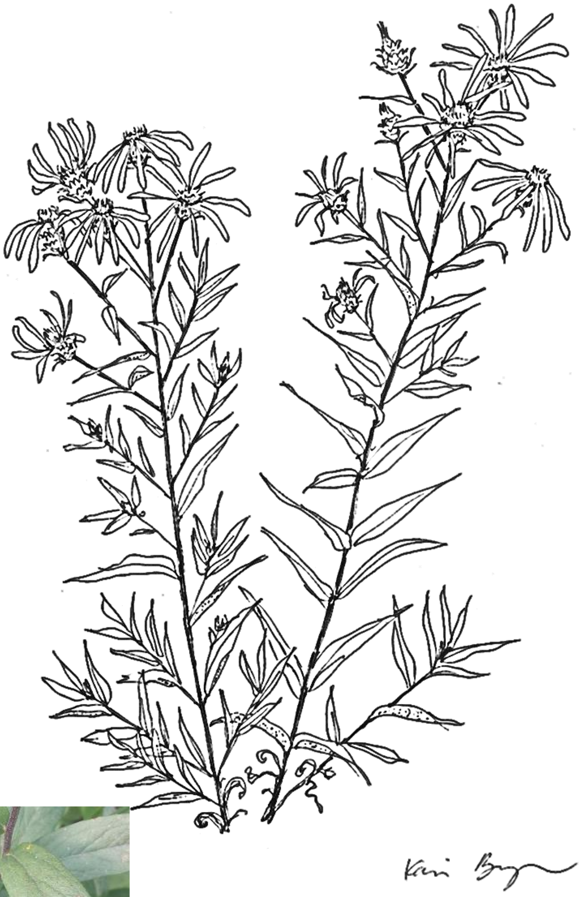

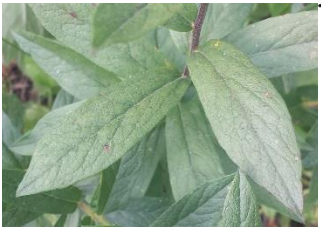

Plant and Leaves:

- 12- 24 inches (30-60cm) tall unbranched stems
- Reduced, scale like lower leaves. Upper leaves uniform in size, lanceolate or elliptic, 1-2.5 inches (3-7cm) long. Lower surface covered with grey, cottony hairs

Cascade Aster

Aster ledophyllus

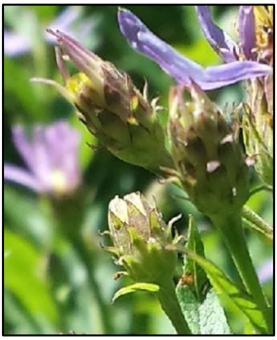

Budding:

- Bud starts tight and green.
- Eventually, you see small light purple petals which look undeveloped and enclose the yellow center

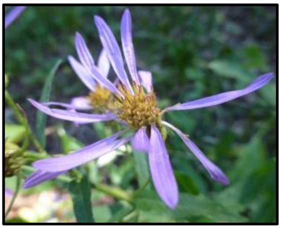

Flowering:

- One to several flowers per stem, 1-1.5 inches (2.5-4cm) broad, with 6-20 ray flowers ("petals") lavender to deep-purple (10-20mm long) surround the small yellow disc flowers (the center of the daisy)

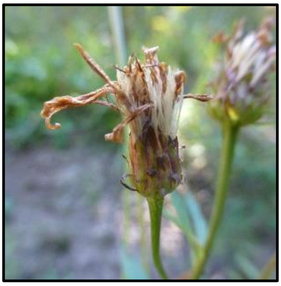

Ripening Fruit:

- A brown head on a single stalk
- No more ray flowers attached, or they are reflexed back and quite brown
- Disc flowers are swelling into fruits with hairy ends

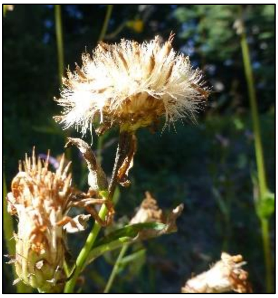

Releasing Seed:

- White hairy seeds will begin to fall off the brown stalk
- Much like Dandelions, Cascade Aster is wind-dispersed

****NOTE**** *Aster* and *Arnica* can look similar when in fruit and releasing seed, but *Aster* seeds are more densely packed.

Glacier Lily

Erythronium grandiflorum

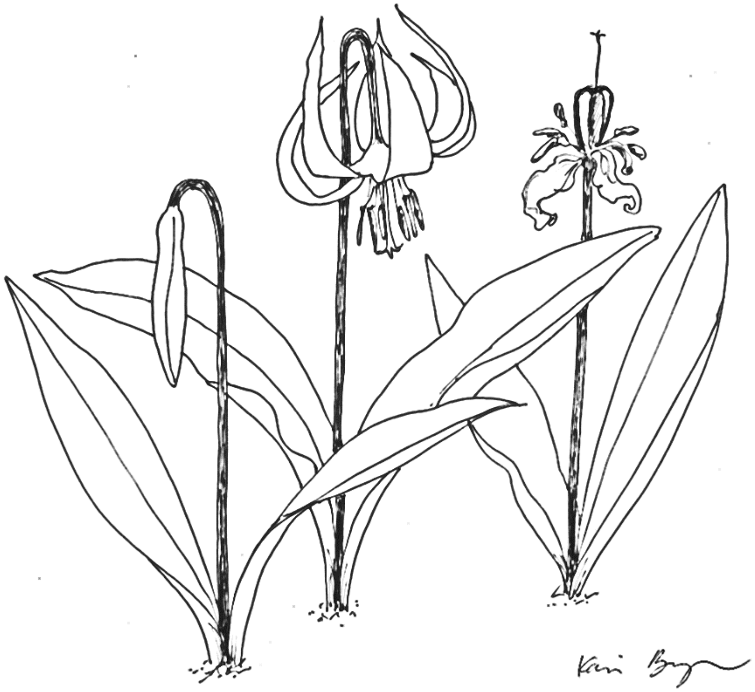

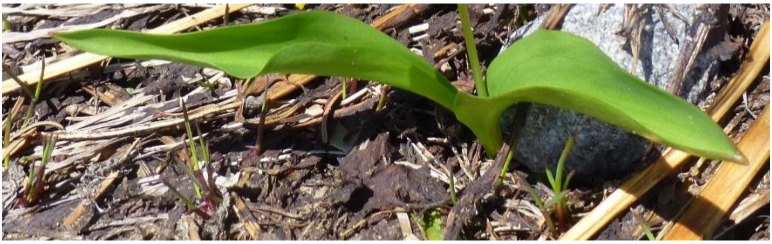

Plant and Leaves:

- Among the first meadow plants to flower, and frequently through the snow. Plant is 6-16 inches tall (15-40 cm)
- Usually two basal leaves 4-8 inches (10 to 20cm) long, narrow gradually to broad blades

Glacier Lily

Erythronium grandiflorum

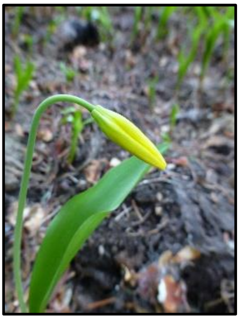

Budding:

- A small yellow bud emerges from a leafless stalk with two basal leaves

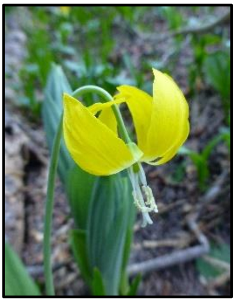

Flowering:

- Usually solitary bell-shaped yellow flowers on 6-12 inch (15-30cm) stem
- 6 yellow 1-2 in (2.5-5cm) petals sweep back to show the white center and the 6 long anthers with stamens
- Flowers frequently graceful, nodding, and facing down

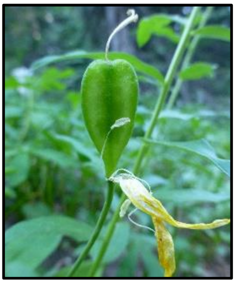

Ripening Fruit:

- Three sided capsule at the end of a long stalk
- Capsule starts green and small (0.2 in or ~0.5 cm) and eventually develops into a larger red-brown capsule (up to 1.5 in or 4cm)

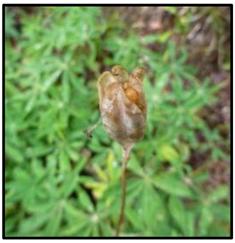

Releasing Seed:

- The capsule opens at the top to release the small round, brown, papery seeds
- The stalk sounds like a rattle when seeds are releasing because capsules, seeds, and stems are so dry (acts as a catapult for long-distance dispersal)

Scarlet Paintbrush
Castilleja miniata

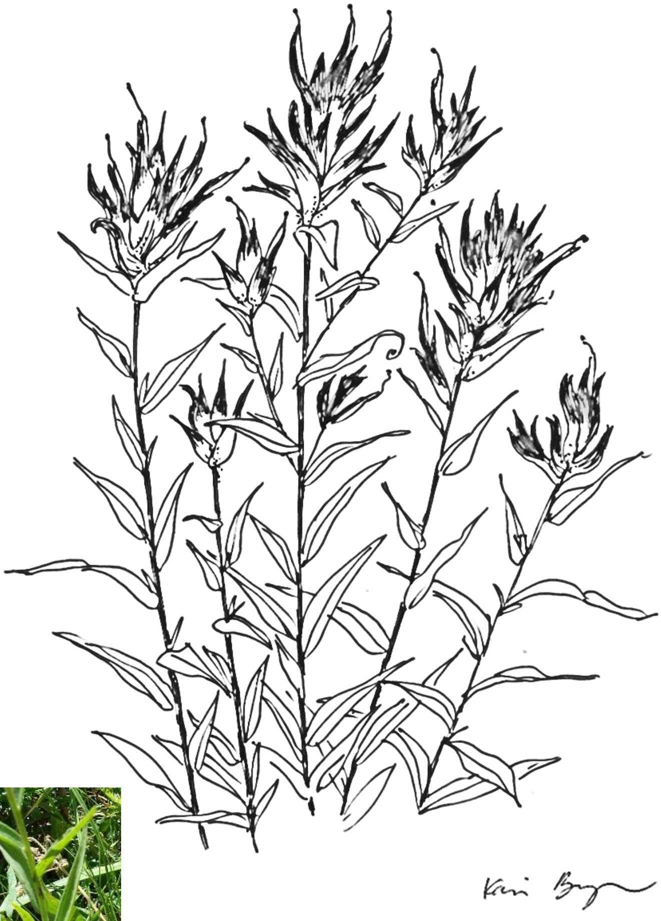

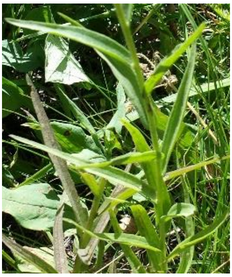

Plant and Leaves:
- Small plant, ~6-12 inches (20-40 cm) tall
- long, thin, lance shaped leaves 1-2 inches (2-4 cm) long

Scarlet Paintbrush

Castilleja miniata

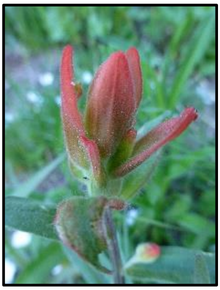

Budding:

- The reddish orange bracts are closed into a loose, orange ball; there are no yellow projections

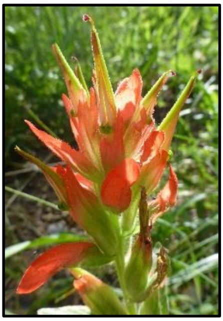

Flowering:

- The orange, brush-like looking "flowers" are not petals, but are called bracts (modified leaves)
- The true flowers are yellow projections that extend beyond the orange bracts
- Count the plant as flowering when you can see the yellow projections

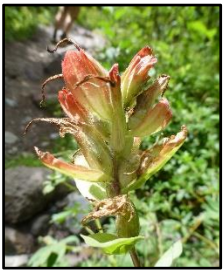

Ripening Fruit:

- Initially, fruits start out green at the base of the dying flowers
- Eventually fruits turn into brown, 2 celled dead looking fruit capsules on the stalk

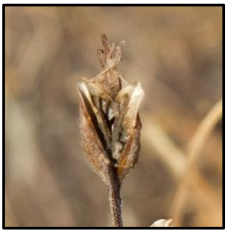

Releasing Seed:

- The seeds release when the capsule cracks open and you can see small brown seeds that look like little pellets (No larger than the bullet point on this page)
- The stalk sounds like a rattle when the seeds are releasing because the entire plant is so dry

Sharptooth Angelica
Angelica arguta

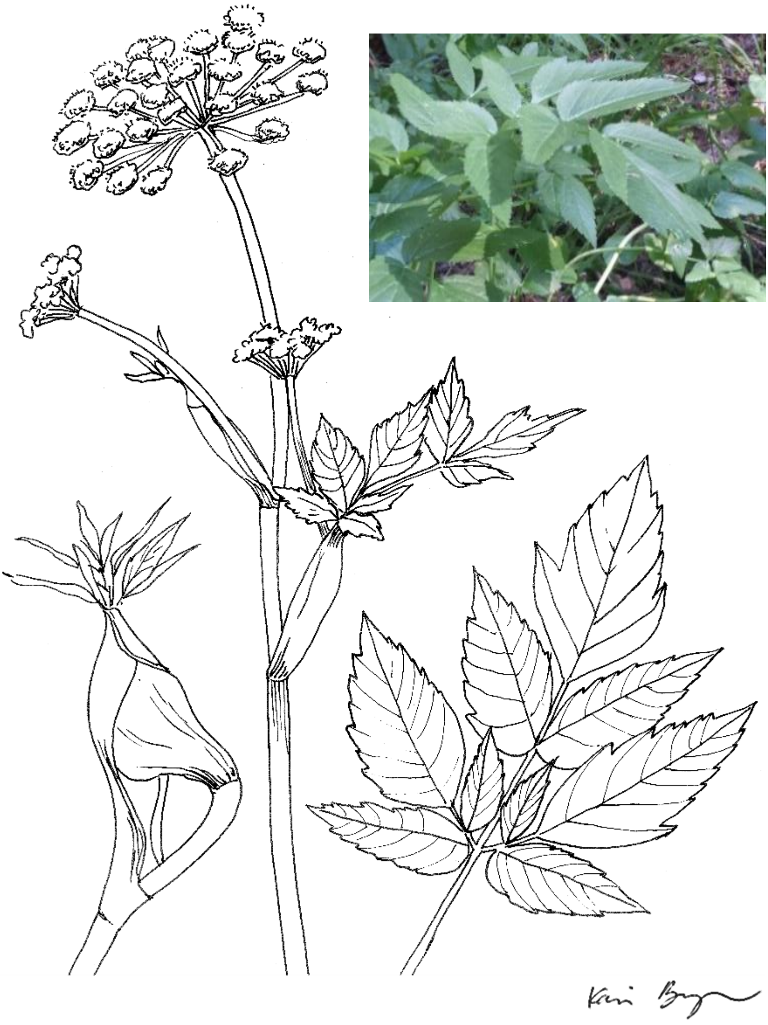

Plant and Leaves:

- Large plant 20-50 inches (50-120cm) tall.
- Basal leaves large and compound, with toothed individual leaflets up to 2 inches (5cm)

Sharptooth Angelica

Angelica arguta

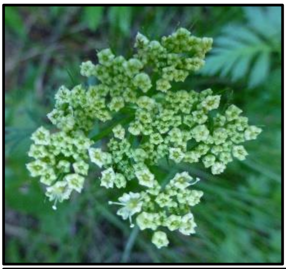

Budding:

- Small white to pinkish buds, clustered in groups of 20-50 at the end of an umbel

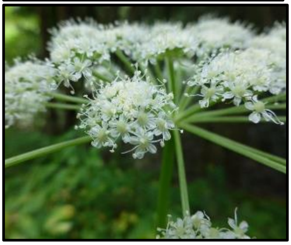

Flowering:

- An umbel containing many white flowers each with 15-30 rays terminating in an umblet.
- Individual flowers have 5 white petals
- Each stalk holds 1 or 2 umbels

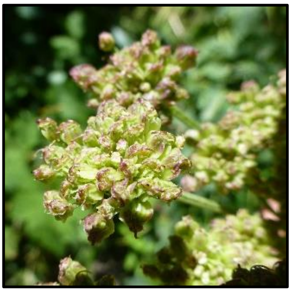

Ripening Fruit:

- Green pod- or seed-like structures replace each flower; many pods comprise the umbel
- Fruits look like anise or fennel seeds –they are all in the same plant family!

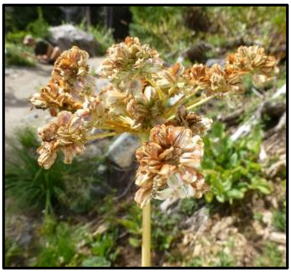

Releasing Seed:

- Green pods turn brown and split open length-wise
- Narrowly winged fruits fall off leaving a white, lacey, hair-like structure

****NOTE**** Individual *Angelica* stalks may have multiple phenophases – make careful observations & be sure to consider which phenophases are present and absent.

Sitka Valerian

Valeriana sitchensis

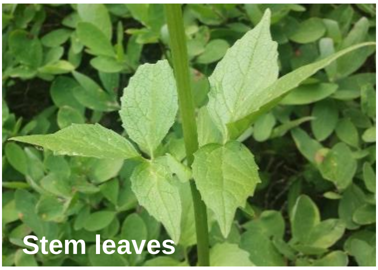

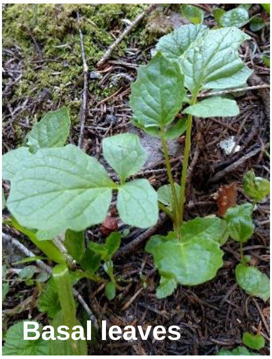

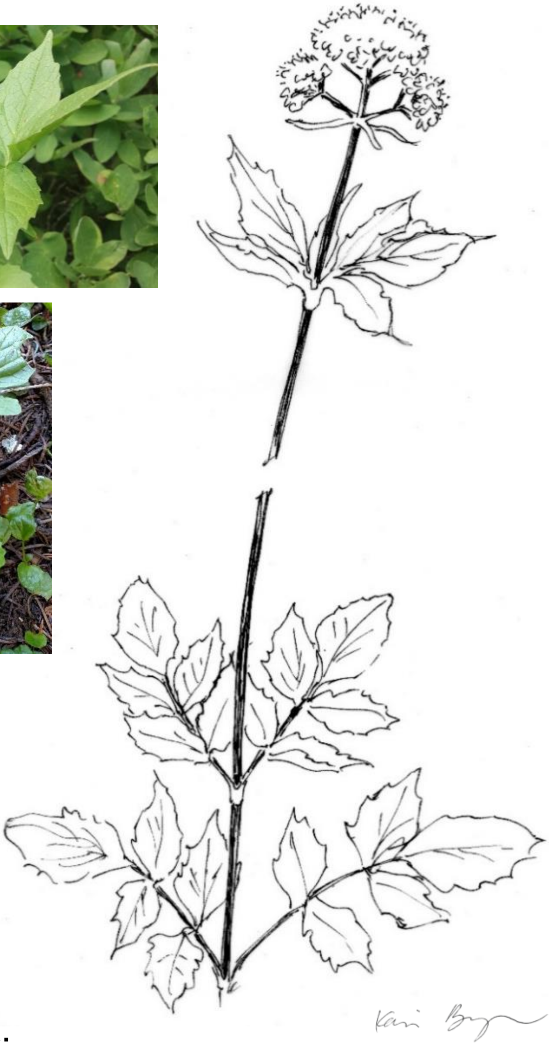

Plant and Leaves:

- Plant 12-48 inches tall (~30-120 cm); robust, dark purplish green stem with leaves and flowers / fruits cluster.
- Leaves 1-3 inches (3-8 cm) long, coarsely toothed and lanceolate to ovate / roundish; basal leaves have long stalks. Basal leaves on non-reproductive plants are more rounded.

Sitka Valerian

Valeriana sitchensis

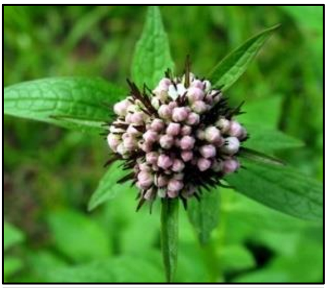

Budding:

- Small white/pinkish buds on top of a square stem that can be 1-4ft (60-120cm tall)

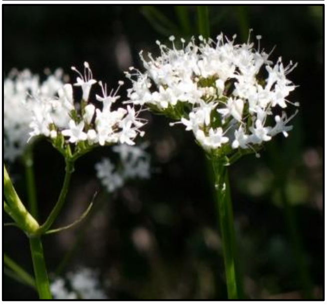

Flowering:

- The flowers are white and fragrant, and sometimes pink
- Each corolla is 6-8 mm long and has 3 stamens
- Flowers cluster into slightly-rounded umbels

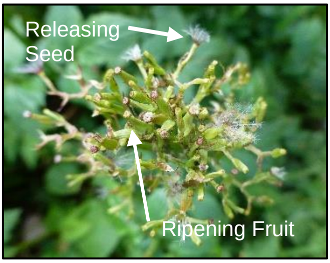

Ripening Fruit:

- Flowers fall off, leaving a vase-like structure which is the fruit

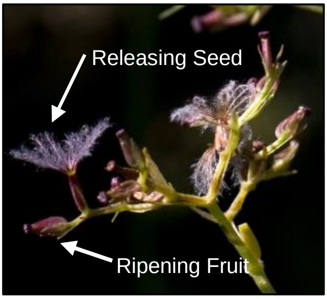

Releasing Seed:

- When fruits release, feathery hairs protrude from the vase for wind dispersal

****NOTE**** It is common for individual *Valeriana* stalks to have multiple phenophases – make careful observations & be sure to consider which phenophases are present <u>and</u> absent.

Subalpine Lupine

Lupinus arcticus

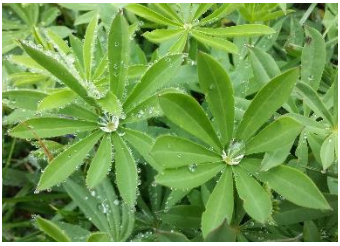

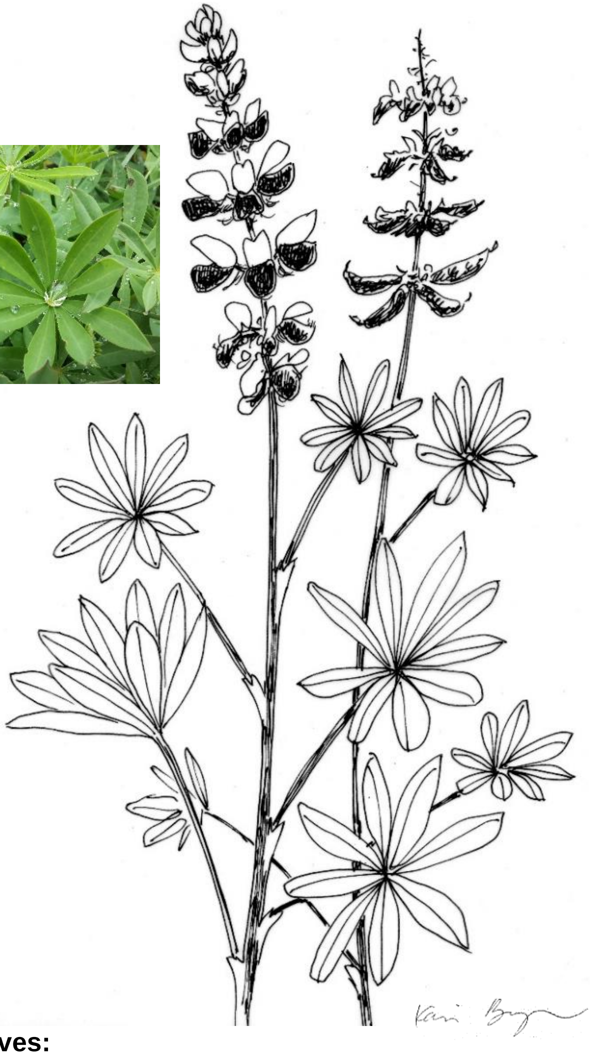

Plant and Leaves:

- Hairy stems are 6-16 inches (15-40 cm) tall and usually un-branched
- The long, stalked, compound leaves bear 5-7 slightly hairy oblanceolate leaflets, 0.6 -1.4 inches (1.5-3.5 cm) long

Subalpine Lupine

Lupinus arcticus

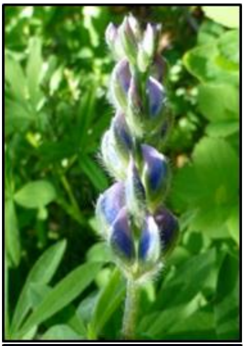

Budding:
- Small purple buds
- Early stages have hairy sepal lining the back of the bud

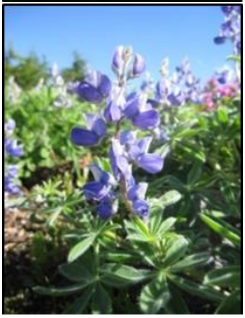

Flowering:
- The flowers are blue to blue-violet and occasionally have white centers
- Each inflorescence has multiple rows (1-6) of 5 flowers encircling the stem

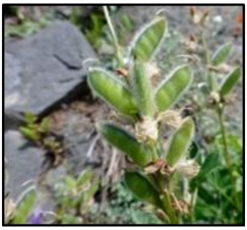

Ripening Fruit:
- Green pods that look like beans replace the flowers
- They are fuzzy and develop to brown pods ~ 1 inch (3cm) long

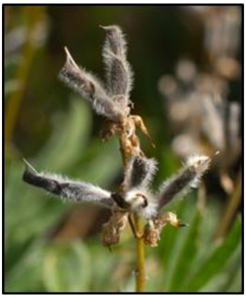

Releasing Seed:
- Once brown, the pods split open, and each half curls back onto itself

****NOTE**** It is common for individual *Lupine* stalks to have multiple phenophases – make careful observations & be sure to consider which phenophases are present and absent.

Tall Bluebell

Mertensia paniculata

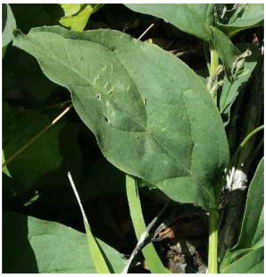

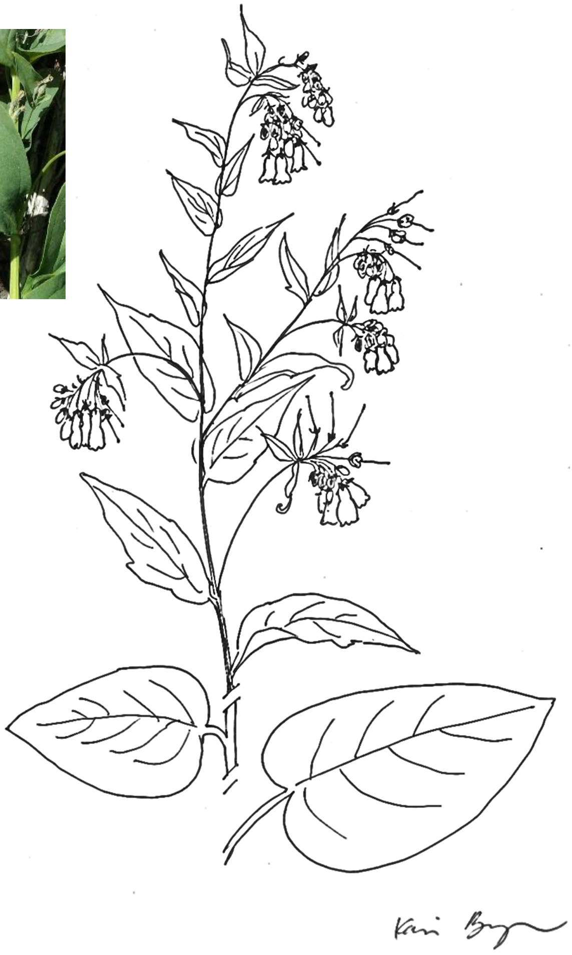

Plant and Leaves:

- Numerous stems 8-60 inches (20-150cm).
- Basal leaves 2–8 inches (5-20cm) long, about half as wide, with long petioles, egg- to heart-shaped
- Stem leaves thin, with obvious veins and short, winged petioles

Tall Bluebell

Mertensia paniculata

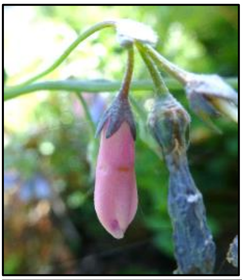

Budding:

- Pinkish bud becoming blue-violet

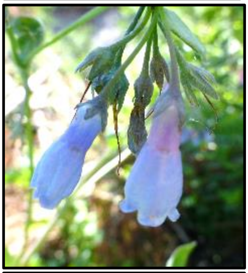

Flowering:

- Blue, occasionally pink, bell shaped flowers, in a loose nodding bundle.
- Flower is a short tube (fused corolla) flaring into 5 lobes, 10-14mm long

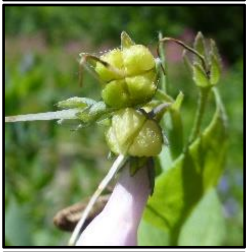

Ripening Fruit:

- 4 green nutlets clustered together
- The elongated white style projects from the center

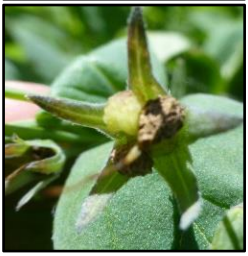

Releasing Seed:

- As fruits begin to dry, nutlets turn brown and wrinkly as style shrivels to a thread-like tail

Identification Tips I

- American Bistort (*P. bistortoides*), left, can often be confused with Showy Sedge (*Carex spectabilis*), on right. Showy Sedge has thin, long grass-like/sedge leaves while American Bistort has larger wider leaves that are dark green with a white midvein. Showy Sedge has smaller white flowers that tend to only be 'showy' on the lower half of the inflorescence (note, showy part are actually male anthers!), while American Bistort tends to flower synchronously within a stalk.

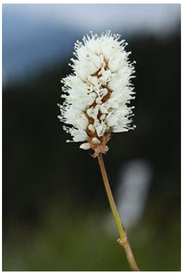

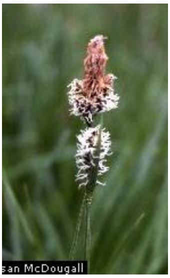

- Cascade Aster (*A. ledophyllus*), left, can be confused with Mountain Daisy (*E. peregrinus*), on right. Mountain Daisy has shorter, more densely packed, and numerous ray flowers (these are what look like the purple flower petals) than Cascade Aster. Mountain Daisy has more basal leaves, which are also wider than Cascade Aster leaves. Note, plots were chosen to not have both species (although this is variable).

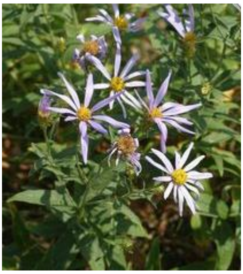

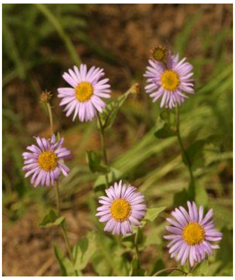

- The fruit and seed phenophases of Cascade Aster (*Aster ledophyllus*) and Broadleaf Arnica (*Arnica latifolia*) can look similar at first glance. The fruit and seed on Cascade Aster are more condensed than Broadleaf Arnica, which are more loosely packed (see species descriptions on previous pages for pictures).

Identification Tips II

- Sharptooth Angelica (*Angelica arguta*) looks quite similar to Canby's Lovage (*Ligusticum canbyi)* and Common Cow-parsnip (*Heracleum maximum*), which both also occur along the Glacier Basin Trail. Although all three species have white flowers in umbels, there are ways to distinguish them. The most reliable way to do so is by looking at the foliage / leaf shape. All three species have compound leaves, but Common Cow Parsnip has three leaflets while Canby's lovage leaves and Sharptooth Angelica have >3 leaflets. Canby's Lovage leaflets are fernlike, Sharptooth Angelica has toothed (but not fern-like) leaflets and Common Cow Parsnip has palmate leaflets that (individually) look a bit like maple leaves. Canby's Lovage tends to be a smaller plant than both the Sharptooth Angelica and Common Cow Parsnip, and tends to occur at higher elevations. See below for pictures outlining differences in the foliage.

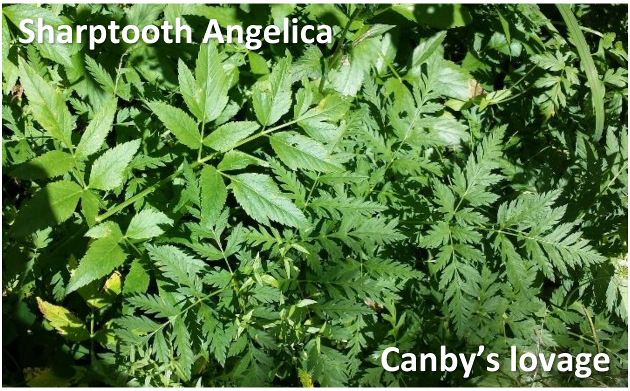

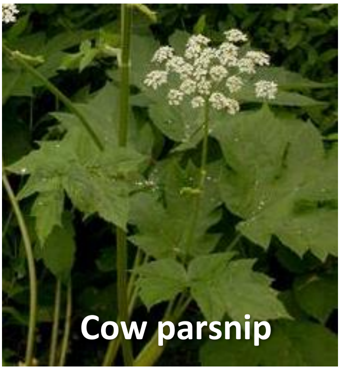

Glossary

Anther – A sack where pollen is stored; found at the end of the stamen filament

Basal – Positioned at the base; “basal leaves” are a cluster of leaves at the base of the stem

Bracts – A specialized leaf, usually at the base of the flower, usually green but can be colored

Calyx – All the sepals of a flower

Compound Leaf – A single leaf that is made up of many smaller leaflets; for example, one lupine leaf is made up of 5-7 leaflets

Corolla – All the petals of a flower

Disc Flower – The central flowers of a flower in the sunflower family; for example, on a daisy each of the small yellow things is a single flower

Filament –The stalk of a stamen that holds the anther

Flower – The reproductive part of the plant; houses the male and female parts

Inflorescence – A cluster of flowers

Lanceolate – Shaped like a blade of a spear; used most often to describe a leaf shape

Oblanceolate – A shape that is opposite of lanceolate, where the widest part is at the top; used most often to describe a leaf shape

Oblong – An elongated, rectangular shape

Obovate – Egg-shaped, the widest part above the middle

Ovary – The expanded basal part of the pistil, containing the ovules

Ovate – Egg-shaped, the widest part below the middle

Ovule – An unfertilized fruit

Petal – An individual part of the corolla, generally colored

Petiole – A leaf stalk

Pistil – The female part of the flower, consisting of an ovary, a style, and a stigma

Ray flower – Outer, showy flower of a flower in the sunflower family; each “petal” is actually a flower!

Sepal – An individual part of the calyx, usually greenish and underneath the petals

Sheath – A thin tissue at the base of a leaf that encircles, or partially encircles the stem

Stamen – The male part of the flower, usually a stalk-like filament with a pollen-producing anther on top

Step – When walking normally, each time either foot hits the ground is one step. For example, Left, Right, Left, Right is counted as 4 steps.

Stigma – The portion of the stigma where pollen lands to start the fertilization process

Style – The slender stalk that connects the stigma to the ovary

Umbel – An inflorescence in which multiple flower stalks radiate from a common point

Species occurrence across Glacier Basin plots

	1	2	3	4	5	6	7	8	9	10	11	12	13	14	15	16	17
American Bistort												✓			✓	✓	
Bracted Lousewort										✓		✓			✓		✓
Broadleaf Arnica				✓	✓	✓			✓	✓	✓						
Cascade Aster					✓						✓	✓	✓	✓		✓	
Glacier Lily										✓	✓		✓	✓	✓		✓
Scarlet Paintbrush		✓									✓	✓		✓			
Sharptooth Angelica	✓		✓	✓		✓	✓										
Sitka Valerian						✓		✓	✓	✓		✓	✓		✓	✓	✓
Subalpine Lupine				✓	✓	✓	✓	✓	✓	✓	✓	✓	✓	✓	✓		✓
Tall Bluebell	✓	✓	✓				✓				✓			✓			

Notes:

Thank you!

We hope that you enjoyed your hike and we appreciate your help in discovering how climate and climate change influence the wildflowers of Mt. Rainier National Park.

Please don't forget to return your data sheet!
You may drop it off in the brown metal MeadoWatch box near the Glacier Basin Trailhead or mail it to us at:

MeadoWatch
University of Washington - Department of Biology
Box 351800, Life Sciences Building
Seattle, WA 98195-1800

Questions? Comments?

MeadoWatch (mwatch@uw.edu); Janneke Hille Ris Lambers (jhrl@uw.edu); Meera Sethi (meeralee@uw.edu)

All photos taken by Natasha Lozanoff or the HRL lab.
Hand drawn wildflowers by Kari Berger.

This material is based upon work supported by the National Science Foundation under Grant Number NSF DEB-1054012 and NASA NNX14AC34G (Applied Sciences Division). Other sponsors (current and past) include the Department of Biology, the National Park Service at Mt. Rainier, Rite-in-the-Rain, Washington's National Park Fund, the Doug and Maggie Walker Endowed Professorship of Natural History, and 113 donors who contributed to our crowdfunding campaign.

Any opinions, findings, and conclusions or recommendations expressed in this material are those of the author(s) and do not necessarily reflect the views of these agencies.

2019

MEADOWATCH

A Citizen Science Project
Exploring Wildflower Phenology
At Mount Rainier National Park

Reflection Lakes

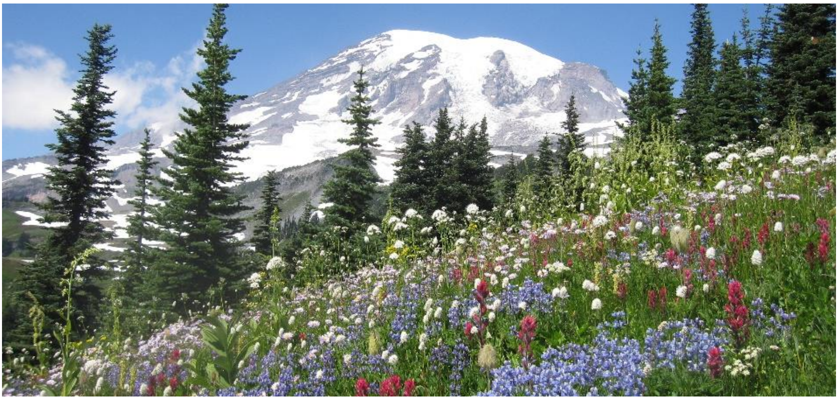

www.meadowatch.org

Background

The goal of **MeadoWatch** is to collect high quality data on the seasonal timing of flowering (i.e. wildflower phenology) in the high mountain meadows of Mt. Rainier. Scientists like us need this kind of data to gain an understanding of the biological impacts of climate change, and we cannot do it without your help! As volunteers, you will help us by monitoring the phenology (see picture below for different 'phenophases') of several plant species over a large gradient in elevation (and climate). This information helps us understand how climate change might alter wildflower phenology, and eventually, could aid resource managers at Mt. Rainier National Park prepare for climate change. By participating, you get the chance to engage in scientific research, and (we hope) gain an understanding of the potential impacts of climate change. Thank you for your help!

Phenology is the study of the timing of recurring life stages (e.g. budding, flowering, fruiting, and releasing seeds) – in other words, the biological manifestation of the seasons. Phenology is frequently cued by climatic variables (e.g. snow disappearance, temperature), and thus, will likely be sensitive to climate change. **Phenophases** are stages of phenology, as illustrated below by Mountain Daisy.

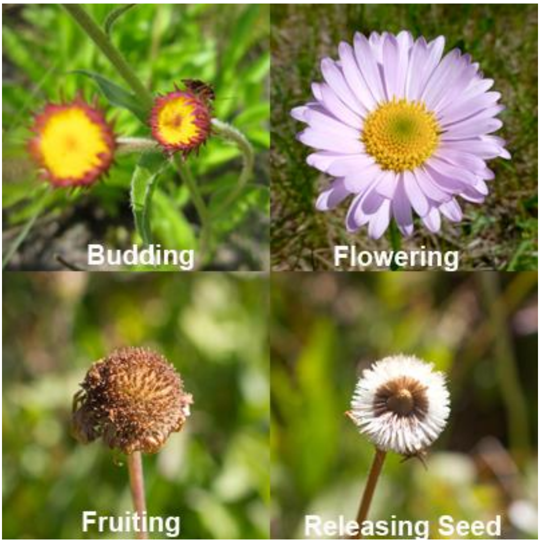

Directions to Reflection Lakes Hike

To help us collect data, you will: **1)** Hike along a system of trails to reach the eleven plots (detailed directions on pages 5-15) **2)** identify the phenophases present and absent for ten focal species (descriptions and photos on pages 18-37) **3)** record what you see on a data sheet (which you should have with you) **4)** then return your data sheet to the brown metal box outside building L-112 at Longmire or mail it back to us.

The eleven plots are located along the east branch of the Lakes Trail, part of the Skyline Trail and the Paradise Glacier trail (see map below). Your hike is 5.5 miles roundtrip and approximately 2,000 feet (600 meters) of elevation gain. To start your hike, park at the easternmost Reflection Lakes parking lot (there are 2) and walk east along the road ~0.1 miles. Follow directions to the Wonderland Trail and High Lakes Trail, and then directions to the first plot (page 5). Our directions to each plot are written assuming you will be hiking uphill.

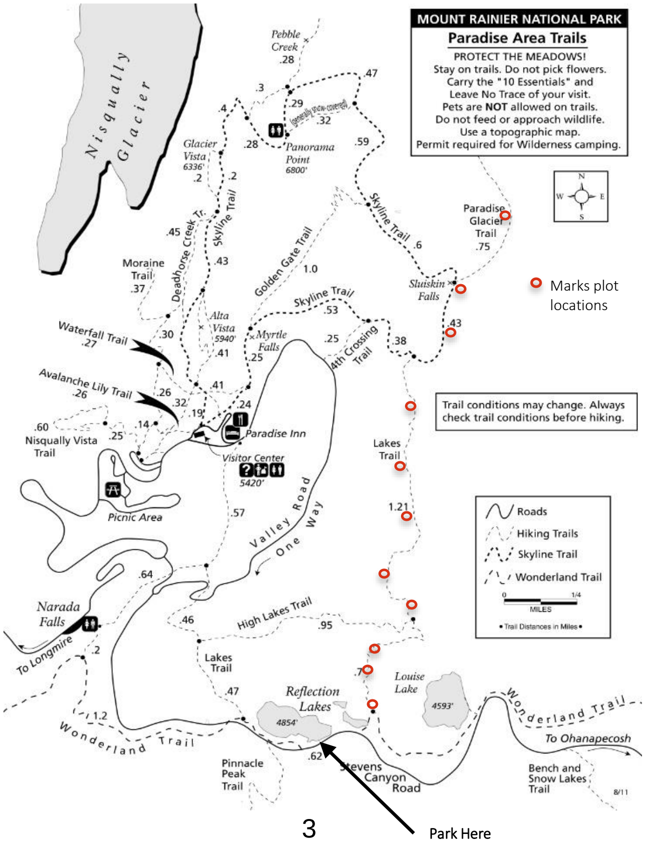

Finding the Plots

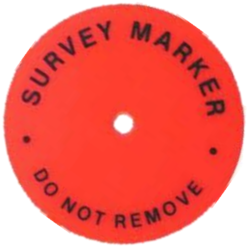

Use the directions and photos within the following pages to find each of the eleven plots. The location of the plot will also be marked by an orange or yellow plastic survey washer located in the trail (see picture right). However, these survey markers might not be present early in the season, although you may see flagging marking our buried microclimate sensors. They may also get covered in trail dust or well-meaning visitors may remove them (we will check this and regularly replace any missing markers). We also provide GPS coordinates (if you have your own unit – WSG 84 datum), but you should be able to find the plots without a GPS unit. Directions to some plots describe streams or small drainages, which may be dry in late summer (but the ditch where the stream once ran will still be visible).

Once at the location, stand with the survey washer between your feet, facing in the direction explained in the plot-specific directions. You will be observing the phenology of our focal species in a rectangular plot made with your arm span as the length of the plot and one arms' length as the width of the plot (see below).

Please help maintain this beautiful natural resource by <u>staying on the trail at all times</u> (you will never need to leave the trail to collect data).

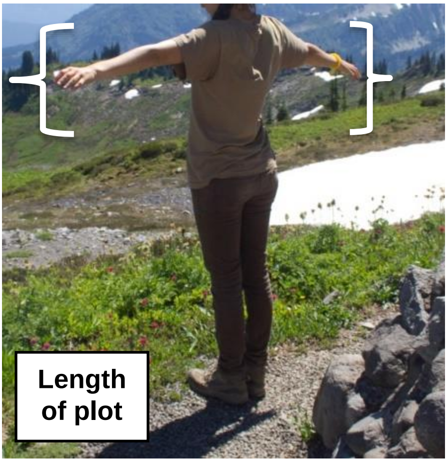

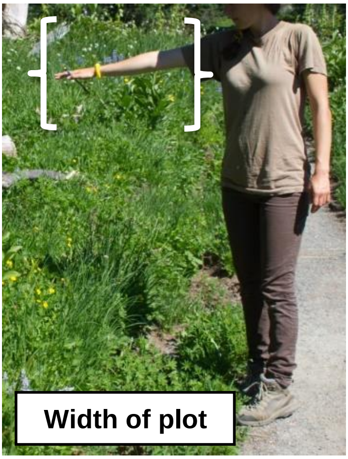

Plot 1

After leaving the road, proceed ~0.1 miles along the Wonderland Trail to the junction with the Lakes Trail (there will be a sign post on the left). Plot 1 is about 20 feet before the turnoff, on your right. The center of plot 1 is on the first of the two erosion beams that make a triangle in the trail.

GPS: 46.77011 lat 121.72317 long
Elevation: 1496 m

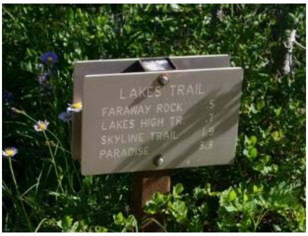

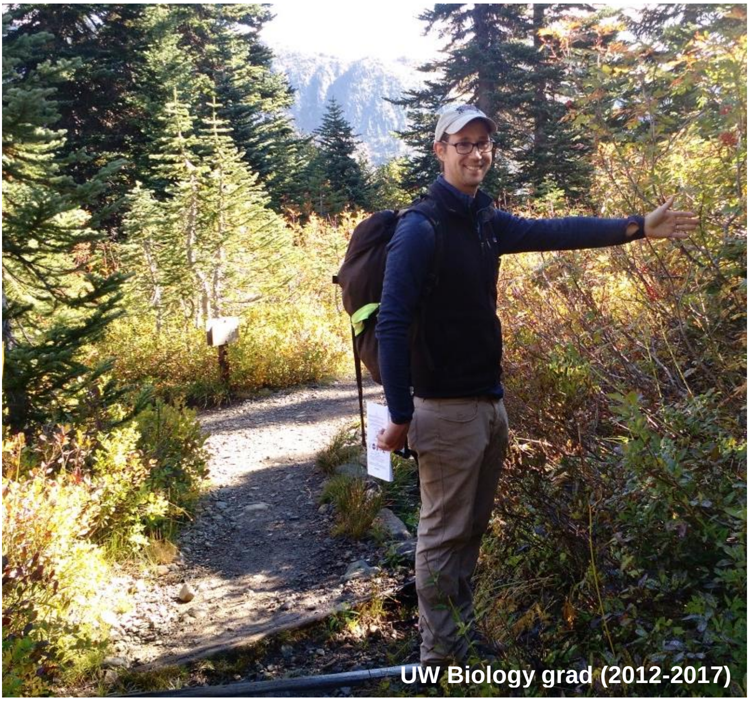

Plot 2

Continue from Plot 1, taking the junction left after the sign to go on the Lakes Trail for ~0.2miles. You will be walking uphill gradually with wooden steps throughout (thank goodness for trail maintenance!). The first time that the trail descends (after leaving Plot 1) you will approach a large stream crossing. In the springtime this stream can be shin-deep and in the summer it is dry – climate does amazing things to our world! (Be cautious while crossing this stream as rocks may be slippery and unbalanced and spring snow bridges may be thin). Plot 2 is on your left as you descend to the stream crossing (but before you cross it). Straddle the wooden beam leading down to the stream and the plot is directly in front of you.

GPS: 46.77334 lat 121.72337 long
Elevation: 1540 m

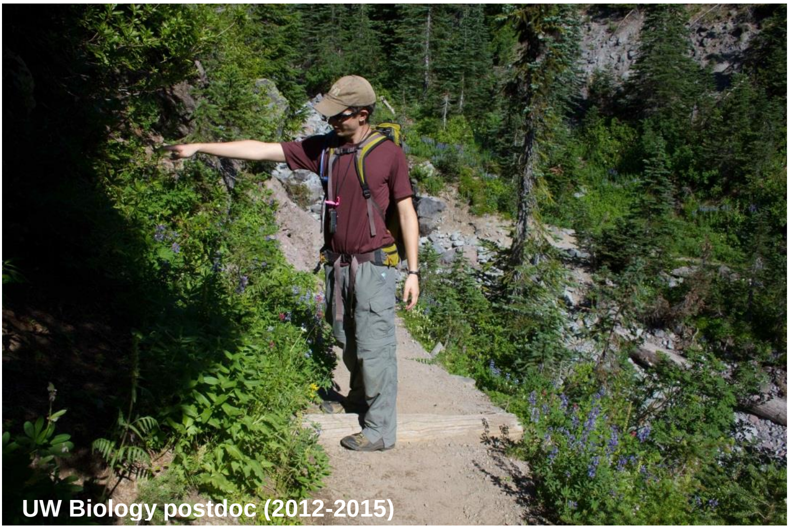

Plot 3

Continue across the stream from Plot 2 and up the trail ~0.3 miles to Faraway Rock. On a clear day you will have a great view of one of the Reflection Lakes and Steven’s Canyon Road. Immediately after Faraway Rock you will pass a pond on your right. Continue uphill for ~0.1 miles and you will come to a switch back to the right (a switchback is a turn in the trail that is nearly 180-degrees). Right after the switchback there will be wooden steps and a series of rocks in the trail. Plot 3 is just past the switch back on the left, after the 2nd step past a small boulder on the left that is the size of a large pillow.

GPS: 46.77499 lat 121.71969 long
Elevation: 1605 m

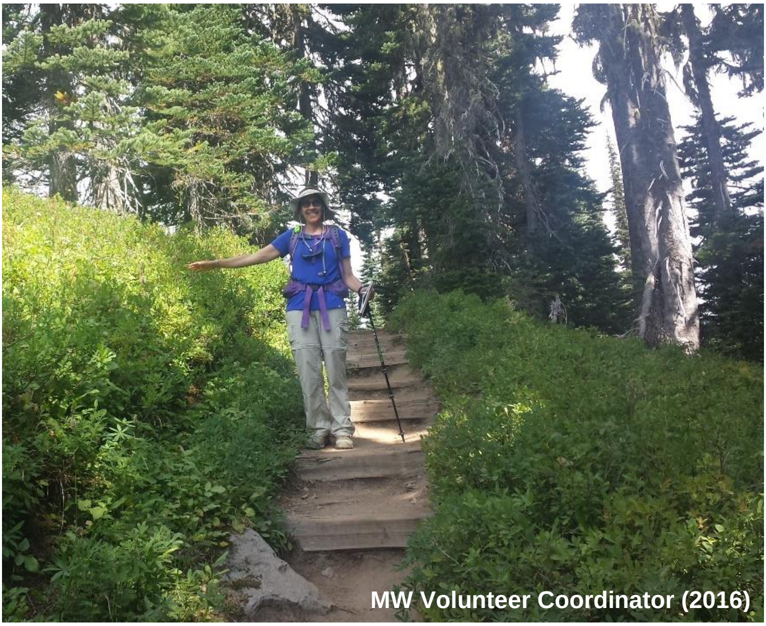

Plot 4

Continuing from plot 3, walk uphill toward the junction with the High Lakes Trail (~0.15 miles). You will pass through a stand of trees and the sign for the High Lakes Trial will be on the right of the trail. About 20 feet after you pass the sign, plot 4 will be located on your right.

GPS: 46.77573 lat 121.72025 long
Elevation: 1612 m

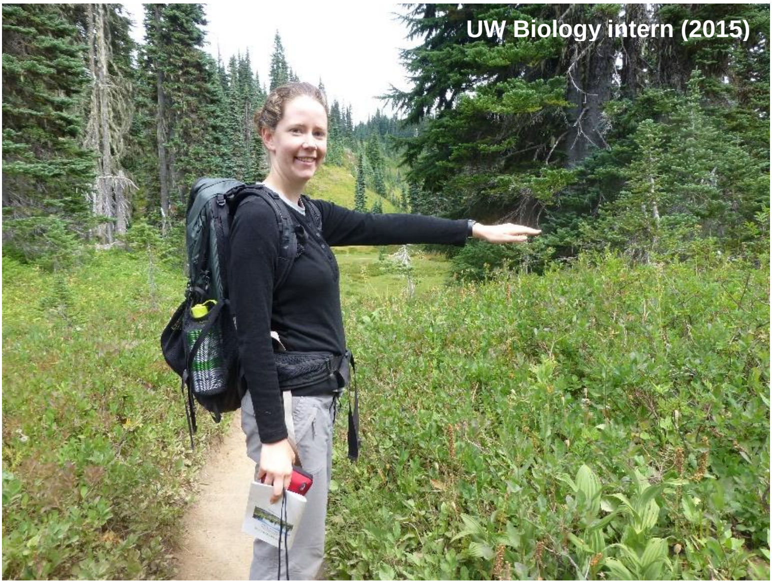

Plot 5

Continue on the Lakes Trail towards Paradise by taking a right at the trail junction. Pass a small pond on your left, and then gradually ascend ~0.2 mi to a large pond on the right. Continue your ascent ~0.1 mi. Just past the pond, the trees will start to thin. On your left, there will be several fallen trees on a hillside. Once you see the triangular-erosion-prevention beams, take four more steps until you reach the next single beam. You will place your feet on each side of this beam and turn to your left to find plot 5.

GPS: 46.777801 lat 121.721638 long
Elevation: 1650 m

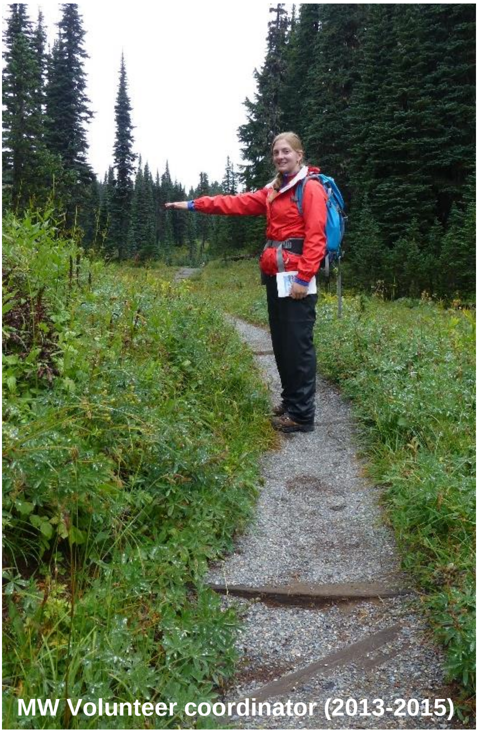

MW Volunteer coordinator (2013-2015)

Plot 6

After leaving plot 5, you will pass through a large clearing and a tree-sprinkled meadow on the left. Then you will climb a small hill out of the meadow and pass another, much smaller pond on the left. In a few more minutes (~0.2 mi) you will climb two small hills. On the second hill there are many cut logs, dead trees and old stumps. Plot 6 is on the left, directly between the log with many branches and the straight one to its left.

GPS: 46.78170 lat 121.71968 long
Elevation: 1680 m

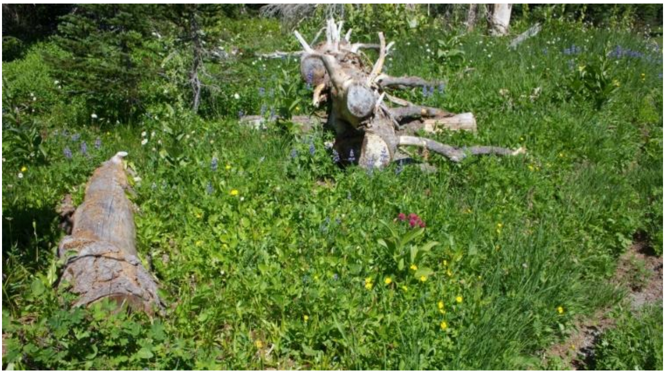

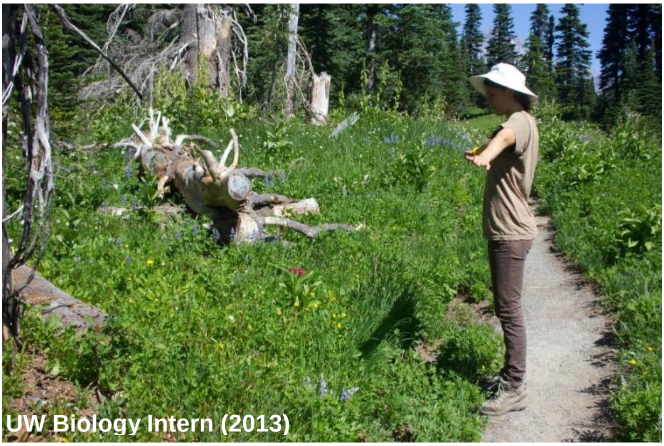

UW Biology Intern (2013)

Plot 7

Moving on from plot 6, you will walk out of the clearing and toward a small stand of trees. You will pass a wonderful view of Steven’s Canyon on your right. Proceeding on, you come to some stone stairs that lead up into a large open meadow with magnificent views of the Rainier summit! Plot 7 is located on your left about 50 feet after the last stone stair. There will be an erosion beam across the trail, stand 1 step uphill from this beam. There is a small Subalpine fir sapling and patch of Pink Mountain Heather just outside and on the uphill side of the plot.

GPS: 46.78403 lat 121.72019 long
Elevation: 1711 m

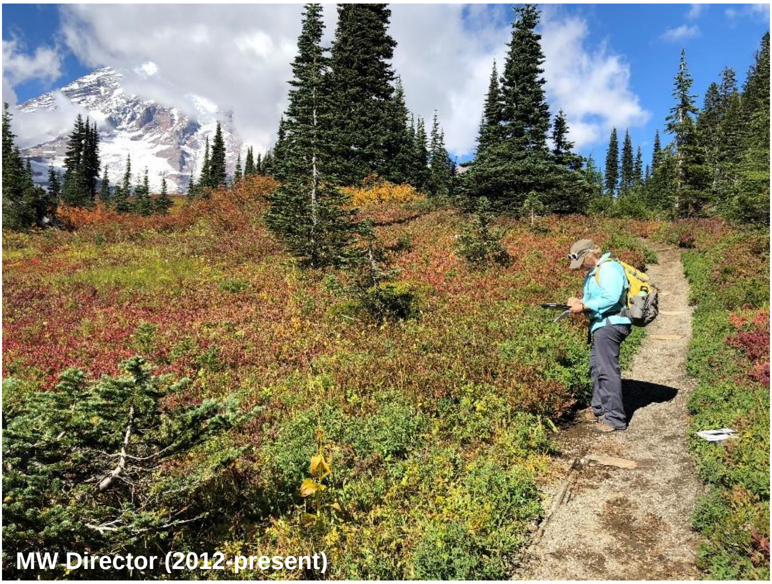
MW Director (2012-present)

Plot 8

Continue on from plot 7 up the trail, at this point, trees are very patchy. You will pass two sparse stands of Subalpine fir on your left as the trail gently winds upward. Keep an eye out for a large fallen log (~25 feet long and 5” in diameter), it is on the left side of the trail. The log is on the last rise before the trail descends into an outwash basin. Plot 8 is at the apex of this hill, about 30 feet beyond the log, on your right.

GPS: 46.78736 lat 121.71936 long
Elevation: 1745 m

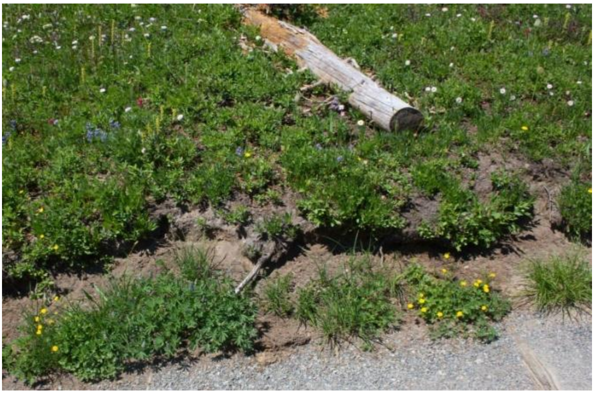

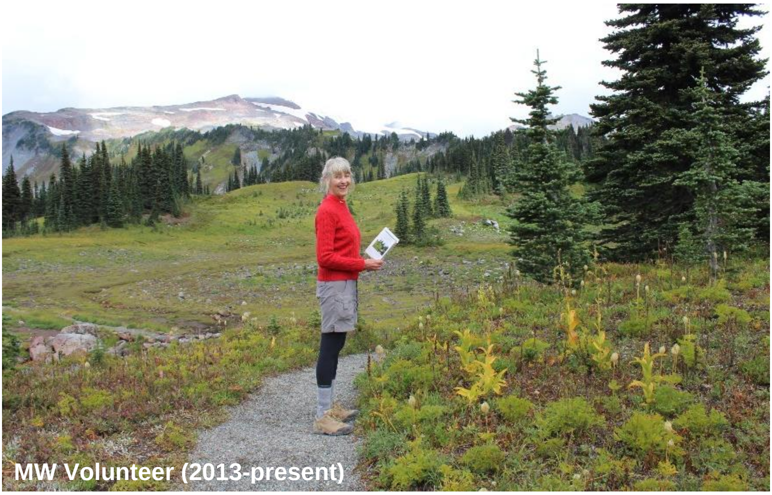

Plot 9

Proceed ~0.2 miles to the junction with the Skyline Trail and turn right onto the Skyline trail toward Panorama point. Proceed ~0.2 miles along the Skyline Trail and after ascending a hill, you will see a large, flat-topped rest boulder on the right side of the trail (this boulder is not to be confused with a 2-boulder rest stop on the left you will pass earlier). Plot 9 is on the right just before the zig-zag in the trail and in the gap between a line of baby trees and the last patch of baby trees, approximately 2/3 of the way towards this last patch of trees.

GPS: 46.79190 lat 121.71693 long
Elevation: 1805 m

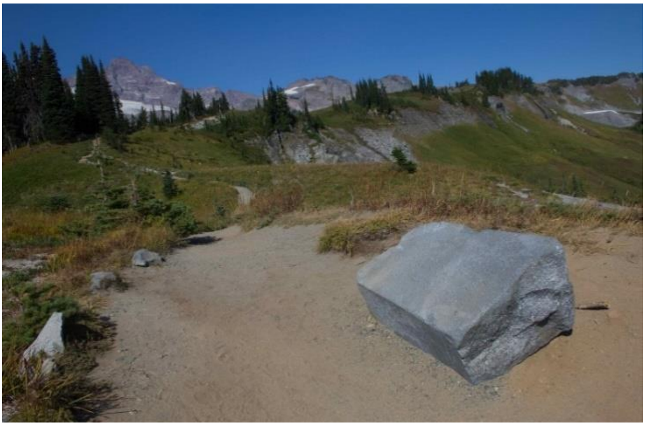

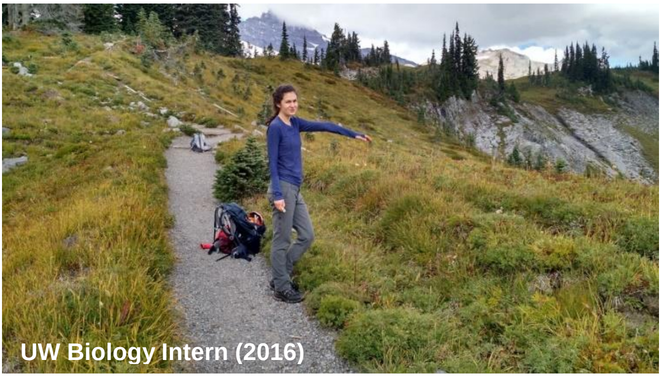

Plot 10

Proceed up the wooden steps (and uphill) about 0.2 miles, or a ~10 minute hike from Plot 9. You will reach the Van Trump Monument that provides a nice place to sit with a beautiful view of the summit. Plot 10 is on the back side of the monument, looking out into the valley. Facing the bench, move to the back left corner and imagine your left hip at the back edge corner of the Monument. You will see some broader stones to your left. The Mount Rainier summit will be directly behind you.

GPS: 46.79403 lat 121.71604 long
Elevation: 1840 m

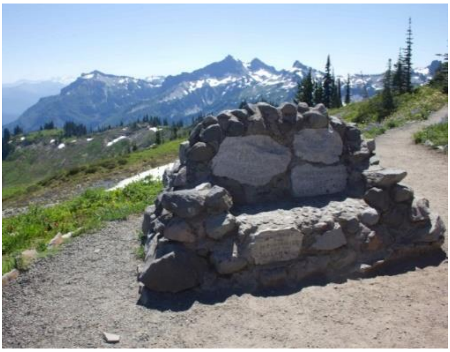

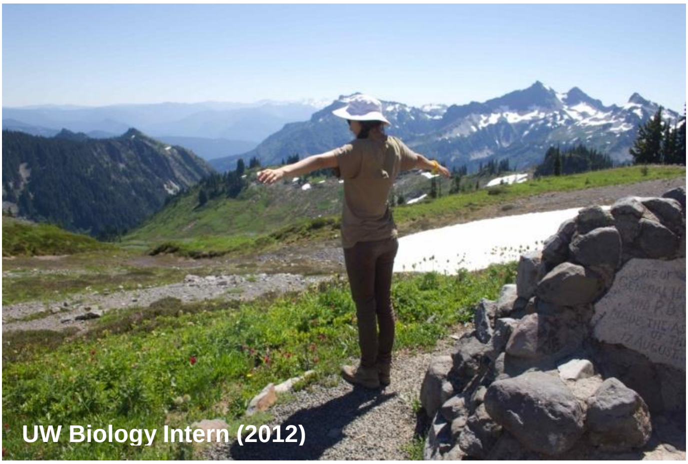

UW Biology Intern (2012)

Plot 11

Just past the Van Trump Monument, you will come to the junction with the Paradise Glacier Trail (there is a large flat boulder in the center of the junction). Turn right along the Paradise Glacier Trail and proceed ~0.4 miles. You will come to a small stream with a pile of large rocks leading up to it. (Warning: the stream may be dry late in the summer.) After about 25 steps from the small stream crossing there will be a big boulder on your right that is half covered with vegetation. Stop 0.5 meters beyond the boulder – Plot 11 is on the right side of trail so your back is to the mountain. You made it to the end!

GPS: 46.79654 lat 121.71194 long
Elevation: 1880 m

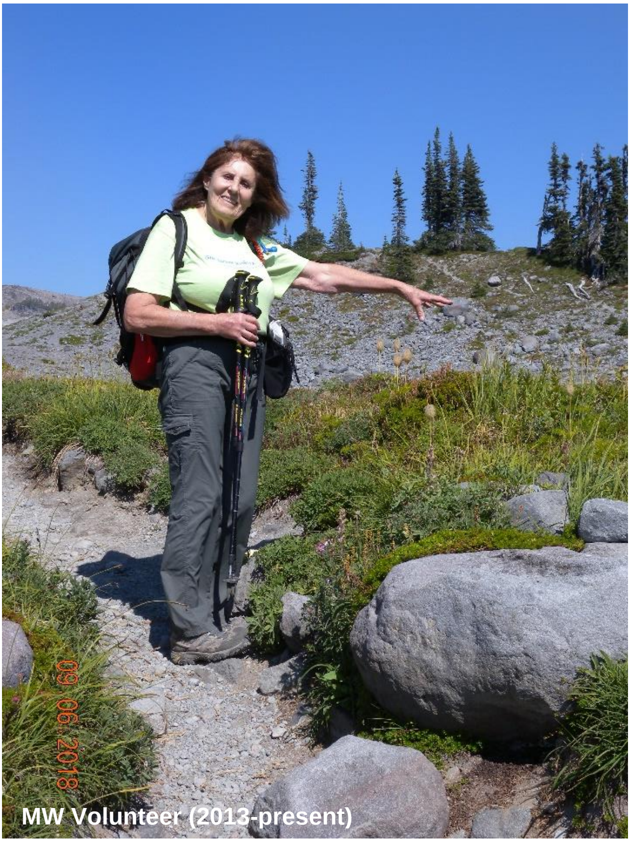

MW Volunteer (2013-present)

Collecting Phenology Data

Our ten focal species and their phenophases (see below) are described in the following pages. The ten species we chose represent a wide variety of plant families, rely on a wide range of pollinators, and bloom at different times of the summer. Additionally, they are relatively abundant (each is in more than one plot, but not in all plots – as will be obvious from your data sheet). Finally, while some of these species have 'look-alikes' in the park, we chose plots for censusing that do not contain any species whose flowers are easy to confuse with our ten focal species.

At each plot, you will collect phenology data by noting which of four phenophases (defined below) are present in the plot. You will often find multiple phenophases – just record which are present and absent – no need to rank their abundance anymore! Of course, these phenophases are a continuum of plant development, and you may observe some bud/flowers/fruits that appear to be between stages. We are trying to categorize the entire process of reproduction into discrete stages. If this is the case, trust your instinct and categorize the plant in the stage you think it is 'closest' to. If you are very unsure, there is a place on the data sheet to note your uncertainty.

Wildflower phenophases you will observe:

Budding – The beginning growth of the flower. The bud will turn into a flower but hasn't yet opened.

Flowering – Usually the showy part of the plant that holds the reproductive parts (stamens and pistils). A plant is flowering when the petals have opened, and the reproductive parts are visible and available for pollination and reproduction.

Ripening Fruit – A fruit develops from the female part of the flower. As the fruit ripens, the seeds within it develop. Fruits can take many forms, from a hard, fleshy capsule to a juicy berry to a feathery tuft on the end of a seed.

Releasing Seed – After the fruit ripens, the seeds are released to begin the next generation. Fruits can be carried away (or dispersed) by gravity, by wind, or by animals.

Each of the focal species pages include a line drawing of each species, drawn by Kari Berger, followed by an individual photo and description of each phenophase (which differs by species).

Additional Data to Collect: Snow

We are asking you to collect other information that can also influence our data, and thus, our ability to understand the link between climate and wildflower phenology.

Snow: We have already found that the timing of wildflower phenology is tightly linked to the date of snowmelt. We have microclimate sensors at each plot which help us monitor the date of snowmelt, but these sometimes fail or go missing. Please help us verify snowmelt dates by noting whether a plot is fully covered by snow (write ‘Y’ in the snow column), half covered (write ‘½‘ in the snow column), or free of snow (write ‘N’ in the snow column).

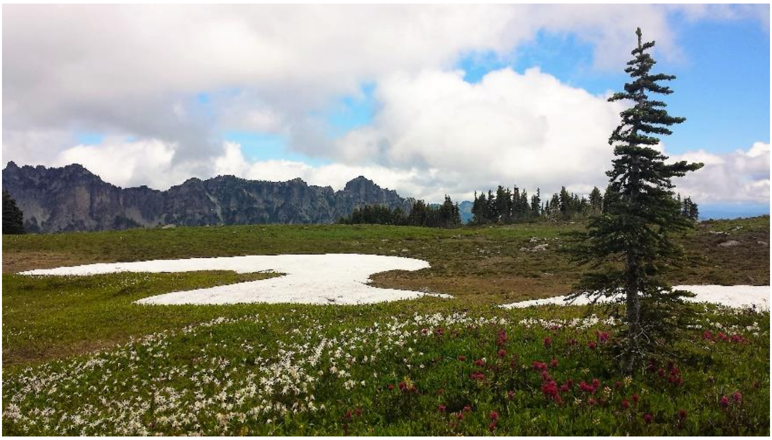

Additional Data to Collect: Herbivory

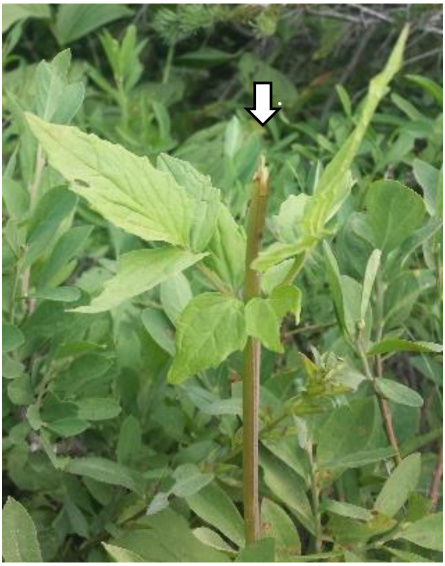

Herbivory / Damage: Many of our focal species are quite tasty to meadow mammals, including deer, elk, and marmots. Often that means that entire stalks will disappear mid-development. If you notice that a reproductive stalk of a focal species is obviously broken, please mark a 'Y' in the herbivory column for that species. Because there could be multiple stalks per species per plot, there may still be other actively reproductive stalks in the plot (continue to monitor those stalks according to the phenophase(s) they display). Don't spend extra time looking for herbivory, and only note this information if it is obvious - it will be easiest to observe in species with tall stalks, like Sitka Valerian (top-right), Western Anemone (bottom-left), and Bracted Lousewort (bottom-right).

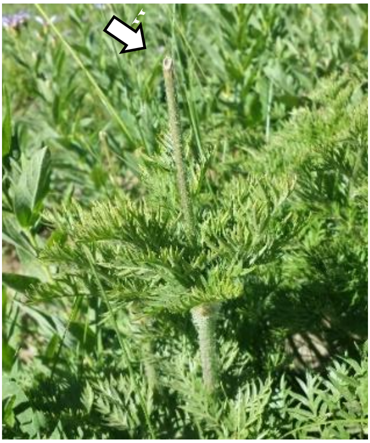

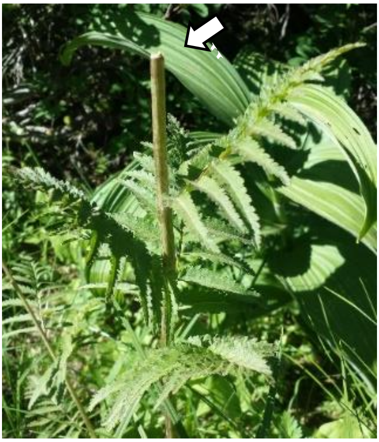

American Bistort

Polygonum bistortoides

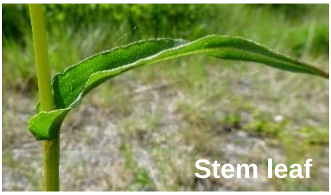

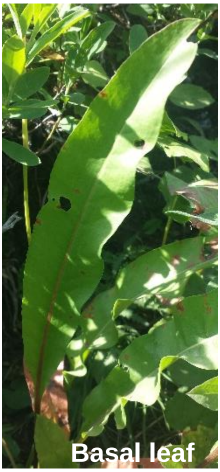

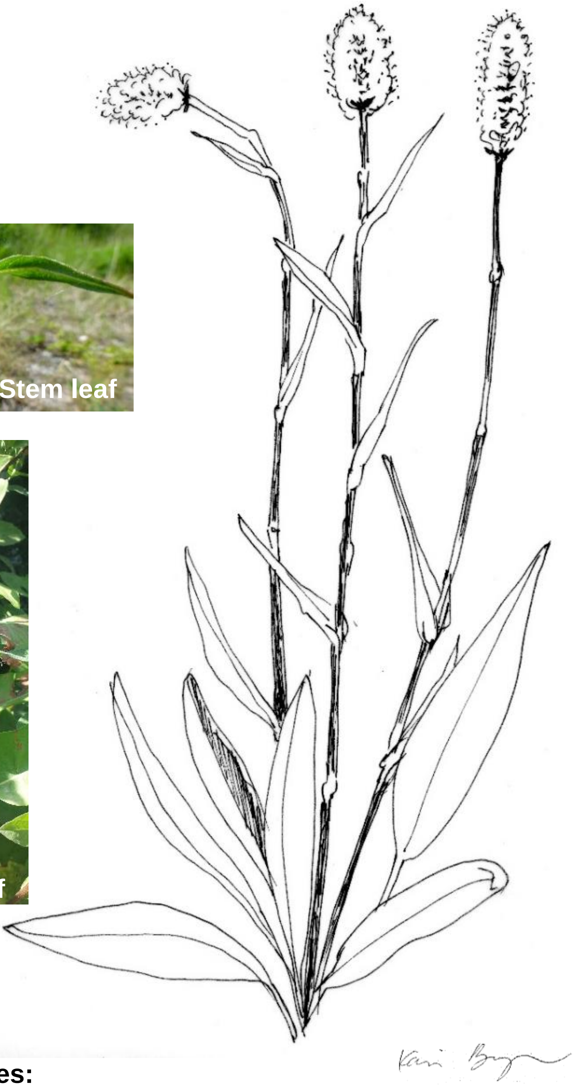

Plant and Leaves:

- Plant tall (12-24 inches), with long unbranched stem
- Leaves mostly basal with distinct petioles (stalks); oblong 4-8 inches long with distinctly light colored midrib. Stem leaves reduced with sheath attachment in place of petioles.

American Bistort

Polygonum bistortoides

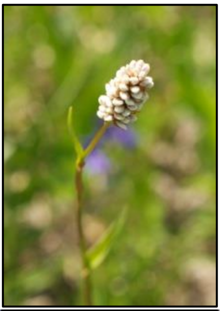

Budding:

- Many small buds cluster on top of a stem that is 1-2 feet (30-60cm)

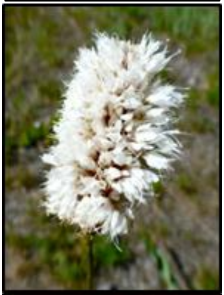

Flowering:

- Flowers are white, bristle-brush-like
- 8 stamens protrude from each blossom, looking like bristles
- The flowers smell awful (this plant is fly-pollinated and this is how it attracts pollinators)

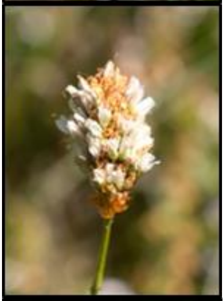

Ripening Fruit:

- The flowers stay attached to the fruits but if you were to touch them they would feel hard (don't touch in the plots!)
- You know the plant is fruiting when the base of each flower turns reddish

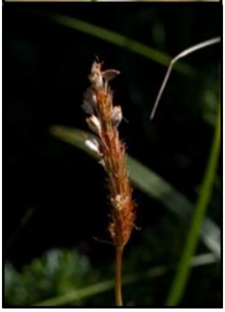

Releasing Seed:

- The white flower-looking structures fall off leaving the bare stalk
- The stalk is rusty-red and still appears hairy

Avalanche Lily

Erythronium montanum

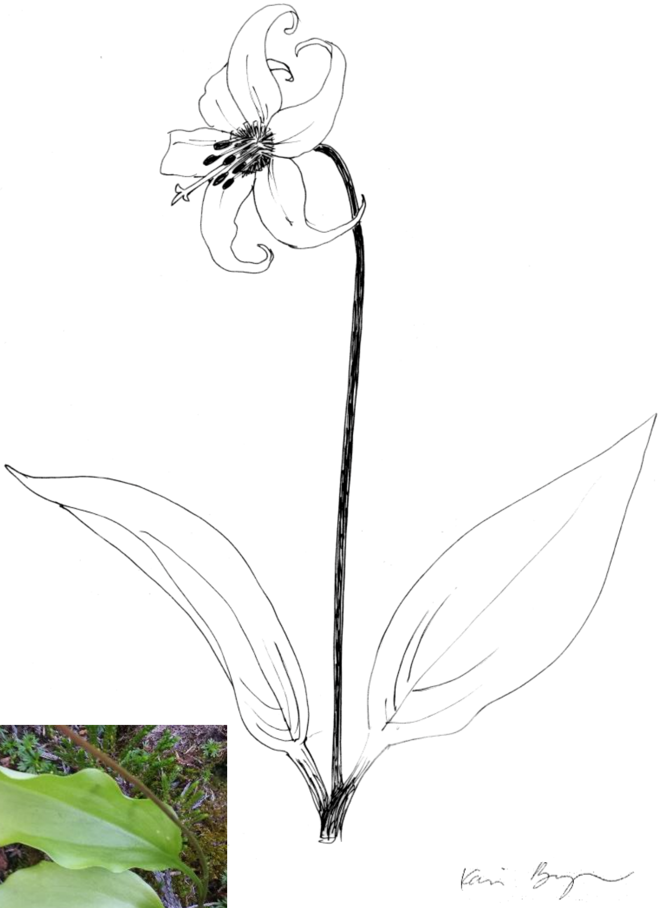

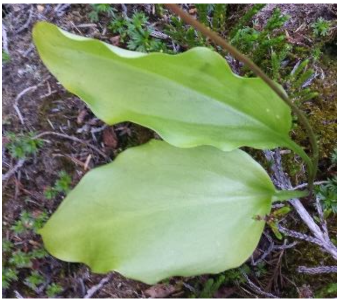

Plant and Leaves:

- Among the first meadow plants to flower, and frequently through the snow. Plant is 6-16 inches tall (15-40 cm)
- Two sole basal leaves; 4-8 inches long and half as wide; leaves fleshy and true green

Avalanche Lily
Erythronium montanum

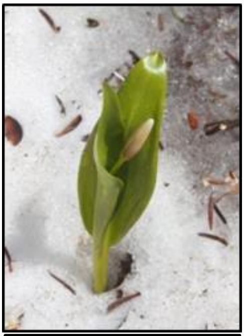

Budding:
- A small white to pinkish bud emerges from a leafless stalk with two basal leaves

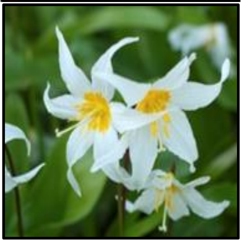

Flowering:
- 1 to 2, occasionally 3+ white flowers on 6-16 inch (15-40 cm) stem
- 6 petals sweep back to show the yellow center and the 6 long anthers with stamens
- Flowers frequently face down

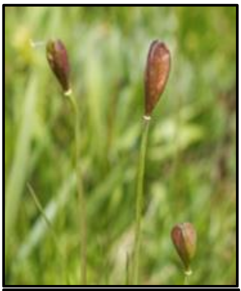

Ripening Fruit:
- Three sided capsule at the end of a long stalk
- Capsule starts green and small (0.2 in or ~0.5 cm) and eventually develops into a larger red-brown capsule (up to 1.5 in or 4 cm)

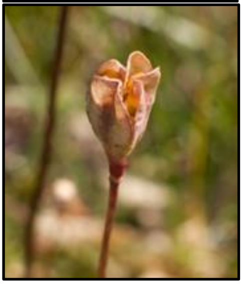

Releasing Seed:
- The capsule opens at the top to release the small round seeds
- The stalk sounds like a rattle when the seeds are releasing because the capsules, seeds, and stems are so dry (this also acts as a catapult for long-distance dispersal)

Bracted Lousewort

Pedicularis bracteosa

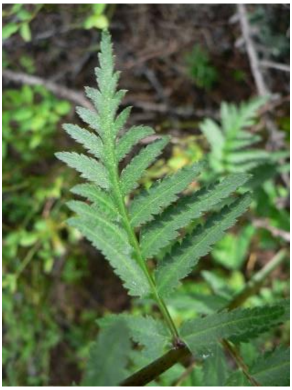

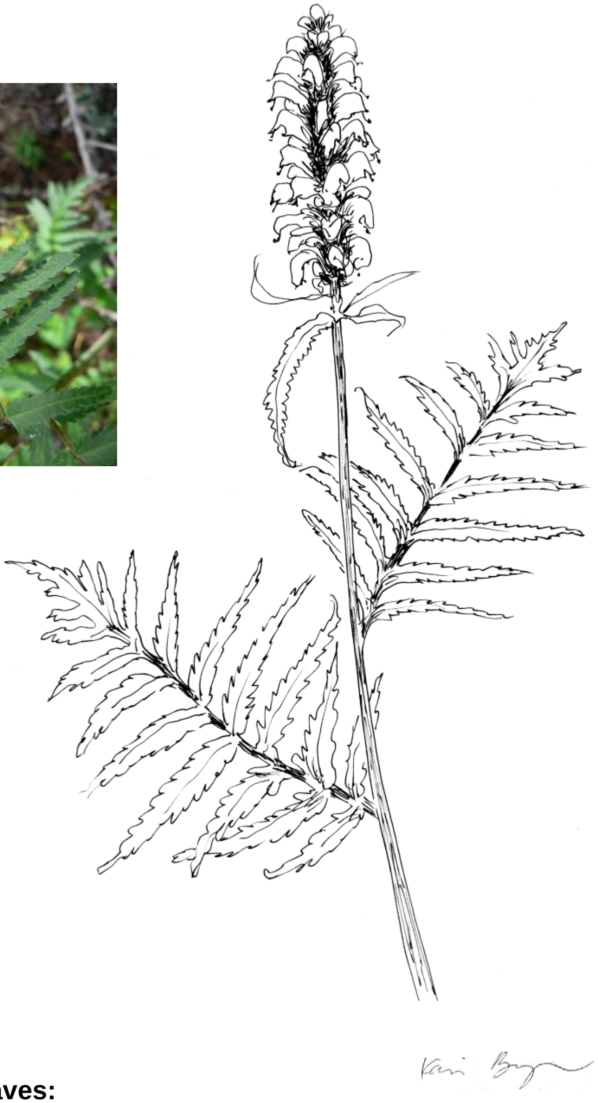

Plant and Leaves:

- Often the tallest meadow plant, reaching 3 feet (90 cm)
- Leaves are fernlike, dark green to purple

Bracted Lousewort
Pedicularis bracteosa

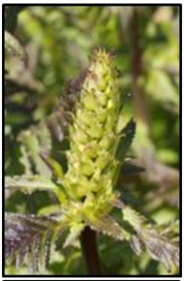

Budding:
- Buds cluster in groups of up to 40 on the tall spike-like inflorescense

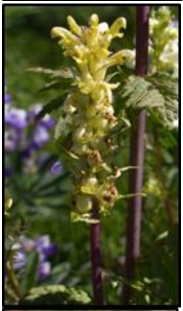

Flowering:
- Yellow flowers crowd together on a long spike on top of the stem
- If you look closely, each small flower has a hood and a lower lip – some say it looks like a parrot’s beak
- Stem and leaves are hairless but the inflorescence is typically hairy

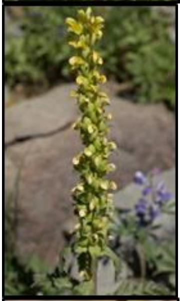

Ripening Fruit:
- The flowers turn into small, green, ball-like capsules that contain the seeds
- There may still be remnants of the flower parts left on the fruits, but the flower parts will be wilted and turning brown
- As the fruit develops the capsules turn from green to brown

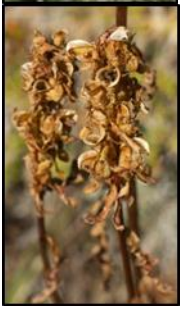

Releasing Seed:
- The fruits crack open at the top (like an urn) and release small seeds
- The stalk sounds like a rattle when the seeds are releasing because the capsules, seeds, and stems are so dry

***** NOTE ***** It is common for individual *Pedicularis* stalks to have multiple phenophases – make careful observations & be sure to consider which phenophases are present <u>and</u> absent.

Gray’s Lovage

Ligusticum grayi

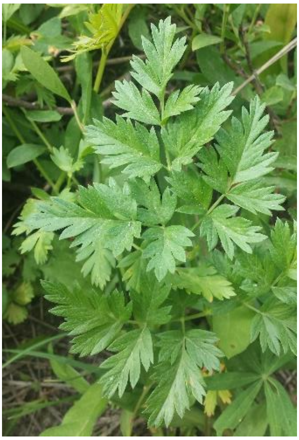

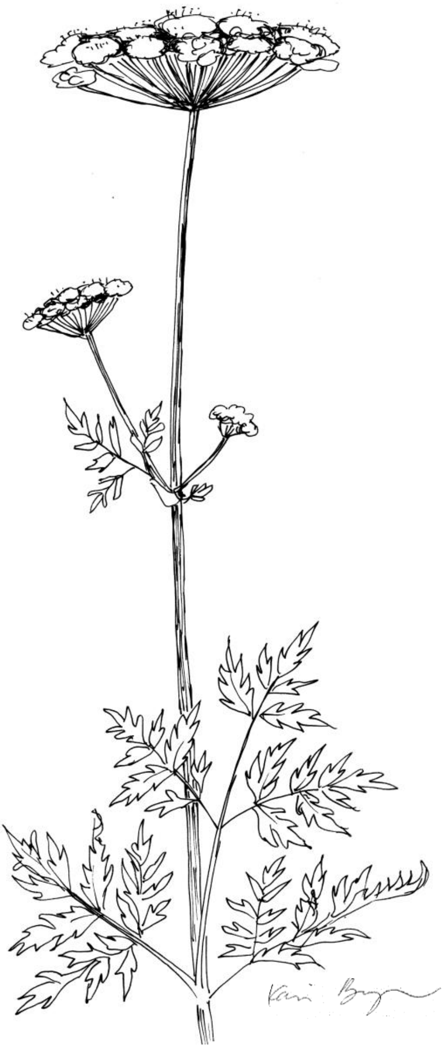

Plant and Leaves:

- Plant 8-24 inches (20-60 cm) tall
- Leaves mostly basal; divided into leaflets with narrowly pointed lobes; carrot-like

Gray's Lovage

Ligusticum grayi

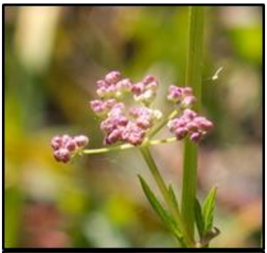

Budding:

- Small white-pinkish buds, clustered in groups of 20-50 at the end of an umbel (upside down umbrella)

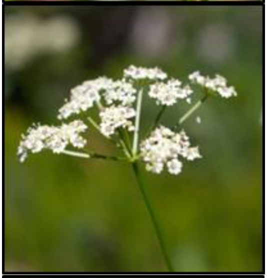

Flowering:

- An umbel with many white flowers with 7-10 rays each
- One stalk holds 1 or 2 umbels

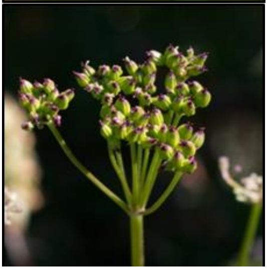

Ripening Fruit:

- Green pod- or seed-like structures replace each flower; many pods on each the umbel
- Fruits look like anise or fennel seeds – which isn't surprising because they are all in the same plant family!

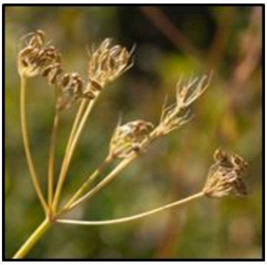

Releasing Seed:

- Green pods turn brown and split open length-wise
- Fruits fall off leaving a white, lacey, hair-like structure

****NOTE**** It is common for individual *Ligusticum* stalks to have multiple phenophases – make careful observations & be sure to consider which phenophases are present <u>and</u> absent.

Magenta Paintbrush

Castilleja parviflora

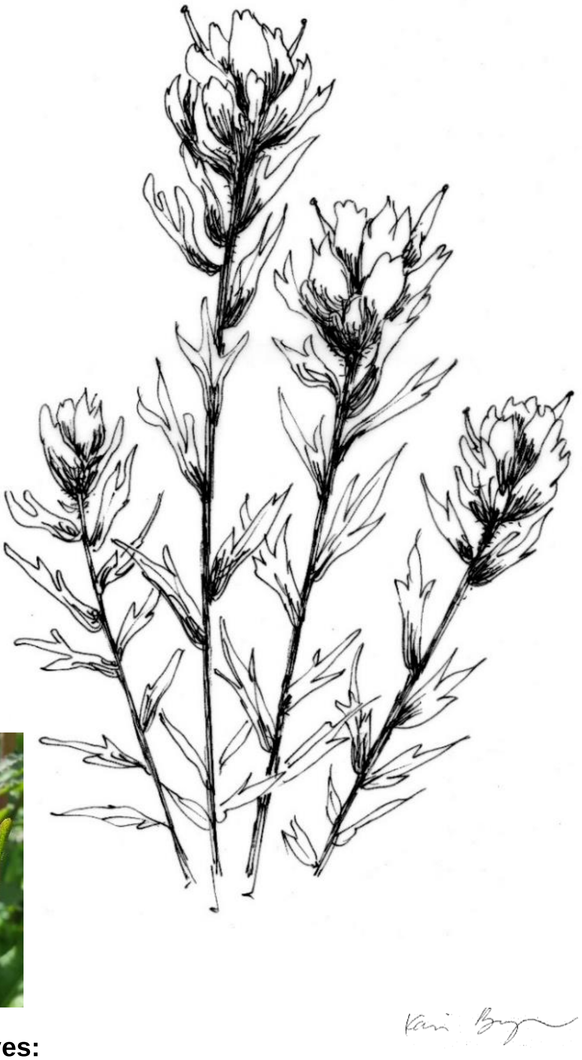

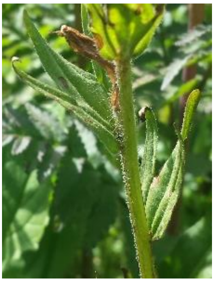

Plant and Leaves:

- Small plant, ~6-12 inches (15-30 cm) tall
- Leaves 1-2 inches (2-4 cm) long with 1 or 2 slender, finger-like lobes

Magenta Paintbrush
Castilleja parviflora

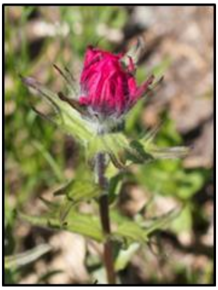

Budding:
- The red/magenta bracts are closed into a loose, dark-red ball; there are no yellow projections

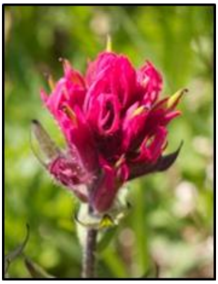

Flowering:
- The magenta, brush-like looking "flowers" are not petals, but are called bracts (modified leaves)
- The true flowers are yellow projections that extend beyond the red bracts
- Count the plant as flowering when you can see the yellow projections

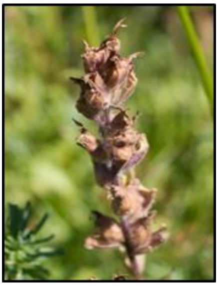

Ripening Fruit:
- Initially, fruits start out green at the base of the dying flowers
- Eventually fruits turn into brown, dead looking fruit capsules on the stalk

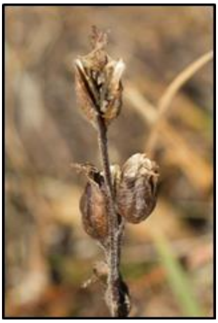

Releasing Seed:
- The seeds release when the capsule cracks open and you can see small brown seeds that look like little pellets They are no larger than the bullet point to the left
- The stalk sounds like a rattle when the seeds are releasing because the capsules, seeds, and stems are dry.

Mountain Daisy

Erigeron peregrinus

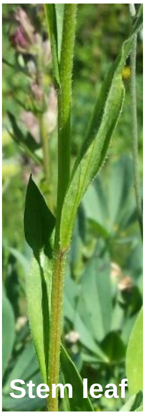

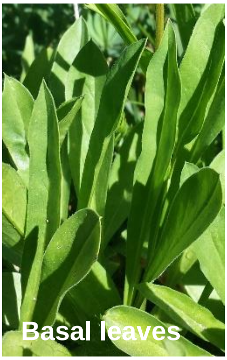

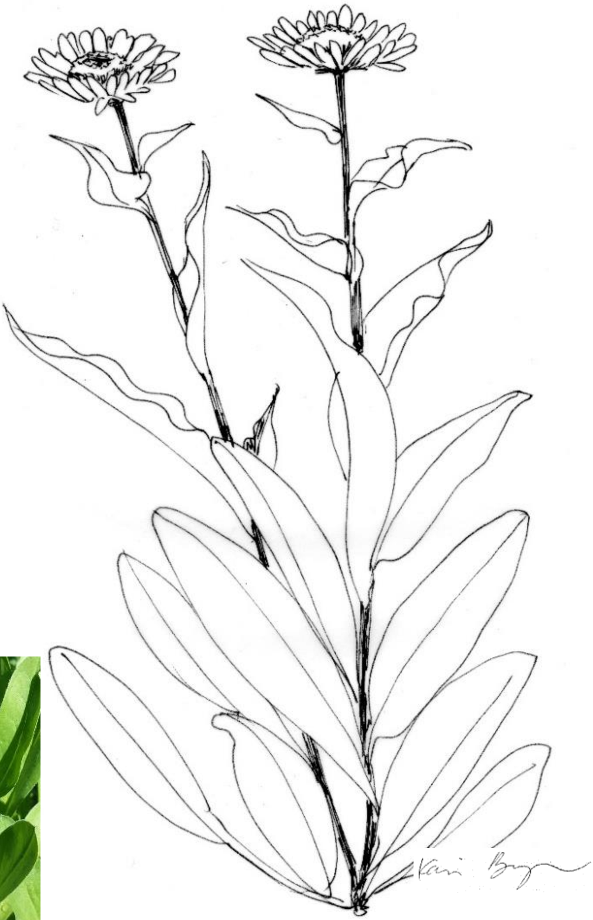

Plant and Leaves:

- Plant ~12-24 inches tall (30-60 cm), unbranched, short stems.
- Basal leaves are alternate, stalked, spoon-shaped 2-8 in. long (5-20 cm). Stem leaves are smaller, lanceolate.

Mountain Daisy
Erigeron peregrinus

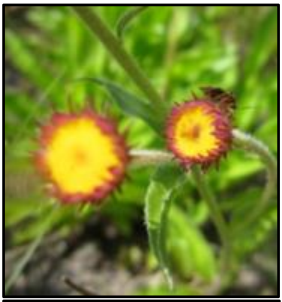

Budding:
- The small pink petals look undeveloped and enclose the yellow center

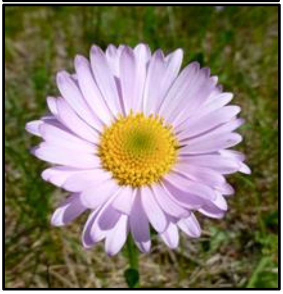

Flowering:
- Usually one flower head per stem, 1-1.5 inch broad, with ray flowers ("petals") lavender to rose-purple surrounding yellow disc flowers (the center of the daisy)
    - See Glossary for definition of Ray and Disc flowers

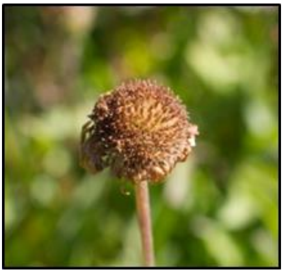

Ripening Fruit:
- A brown head on a single stalk
- No more ray flowers attached, or if so are reflexed back and brown
- Disc flowers are swelling into fruits

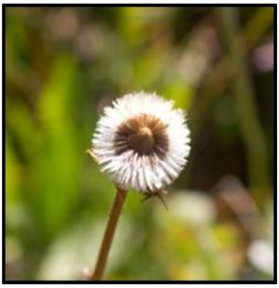

Releasing Seed:
- When some of the seeds have started to disperse, you can see the bare head underneath
- Much like Dandelions, Mountain Daisy is wind-dispersed

****NOTE**** *Erigeron* and *Microseris* can look similar when in fruit and releasing seed, but *Erigeron* seeds are more densely packed.

North Microseris

Microseris alpestris

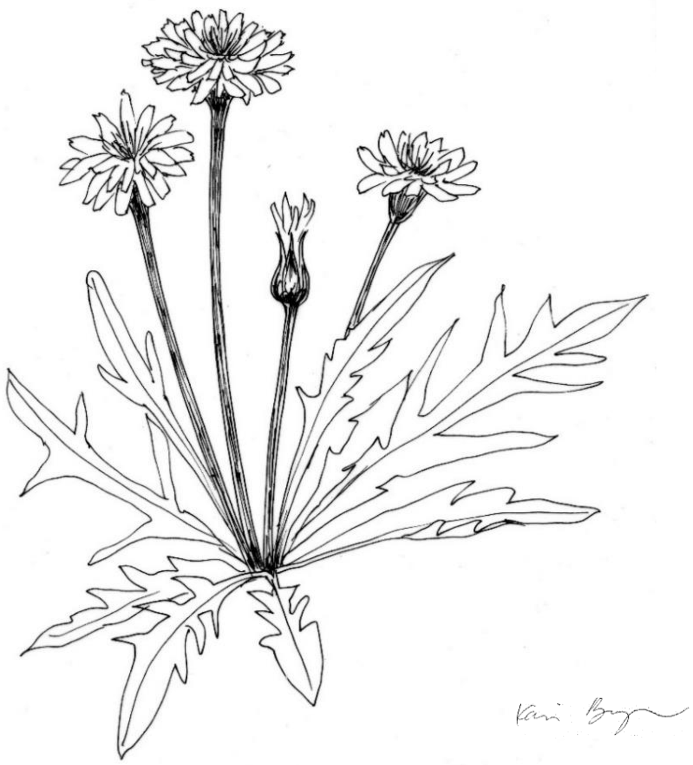

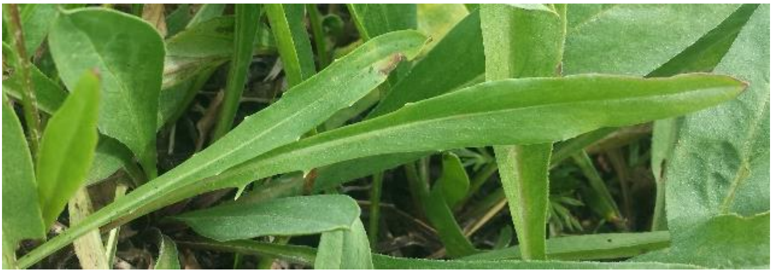

Plant and Leaves:

- Plant erect each stem holding a single flower head; stem hairless below cup of green bracts that hold flower head.
- The leaves are basal, hairless, spoon-shaped to lanceolate, 2-6 inches (5-15 cm) long with a few slender to many teeth

North Microseris
Microseris alpestris

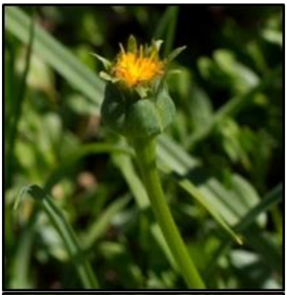

Budding:
- The green sepals enclose the yellow flowers

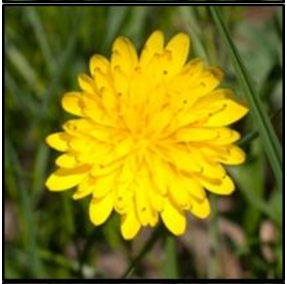

Flowering:
- Bright yellow flower heads are solitary on a leafless stem
- Composed of ray flowers only, heads are 0.7-1.4 inches (2-3.5 cm) broad

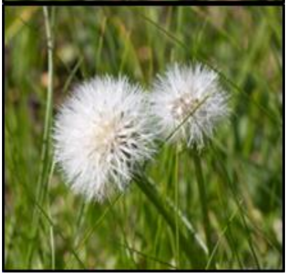

Ripening Fruit:
- The fruits are replacing the flowers, and begin as hair-like structures concealed in a loose ‘bud’. If you gently peek inside the bud you will see white (not yellow)
- Fruits develop to long white bristles, resembling a dandelion

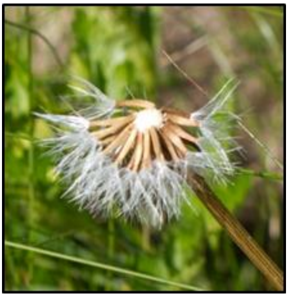

Releasing Seed:
- When the seeds start to disperse, you can see the bare head underneath
- Much like Dandelion and Mountain Daisy, *Microceris* is wind-dispersed

****NOTE****_Erigeron_ and *Microseris* can look similar when in fruit and releasing seed, but *Microseris* seeds are less densely packed.

Sitka Valerian

Valeriana sitchensis

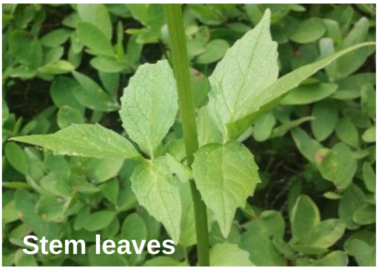

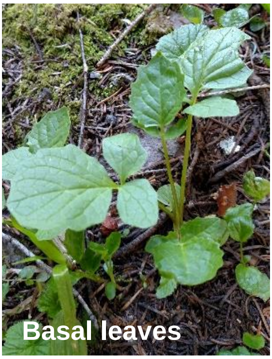

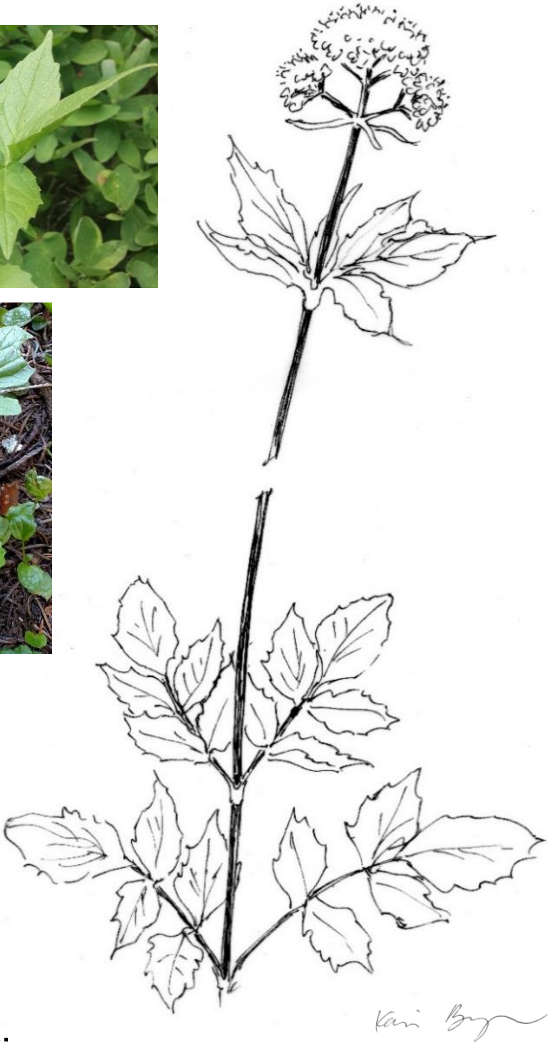

Plant and Leaves:

- Plant 12-48 inches tall (~30-120 cm); robust, dark purplish green stem with leaves and flowers / fruits cluster.
- Leaves 1-3 inches (3-8 cm) long, coarsely toothed and lanceolate to ovate / roundish; basal leaves have long stalks. Basal leaves on non-reproductive plants are more rounded.

Sitka Valerian
Valeriana sitchensis

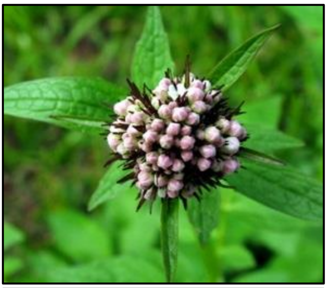

Budding:
- Small white/pinkish buds on top of a square stem that can be 1-4ft (30-120cm tall)

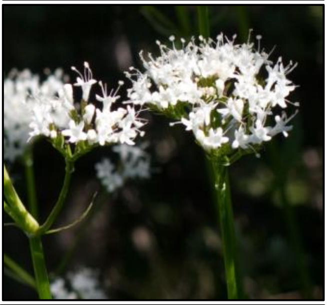

Flowering:
- The flowers are white and fragrant, and sometimes pink
- Each corolla is 6-8 mm long and has 3 stamens
- Flowers cluster into slightly-rounded umbels

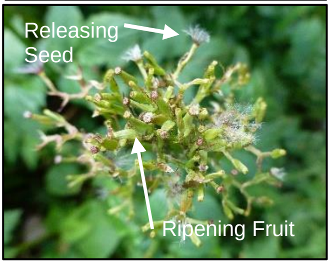

Ripening Fruit:
- Flowers fall off, leaving a vase-like structure which is the fruit

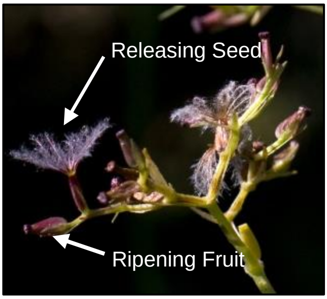

Releasing Seed:
- When fruits release, feathery hairs protrude from the vase for wind dispersal

****NOTE**** It is common for individual *Valeriana* stalks to have multiple phenophases – make careful observations & be sure to consider which phenophases are present and absent.

Subalpine Lupine

Lupinus arcticus

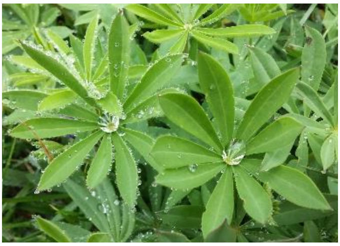

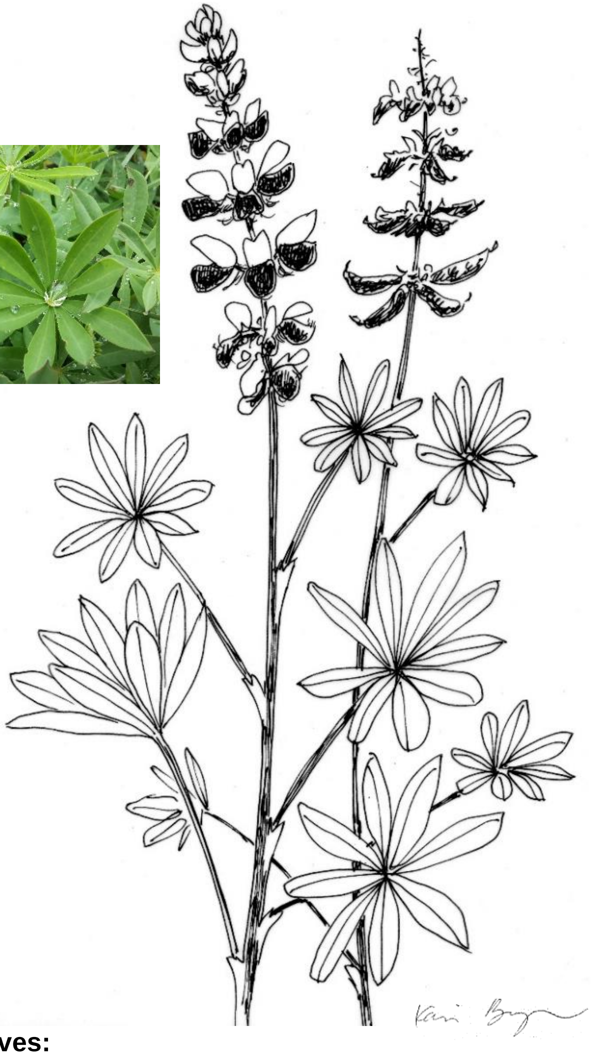

Plant and Leaves:

- Hairy stems are 6-16 inches (15-40 cm) tall and usually un-branched
- The long, stalked, compound leaves bear 5-7 slightly hairy oblanceolate leaflets, 0.6 -1.4 inches (1.5-3.5 cm) long

Subalpine Lupine

Lupinus arcticus

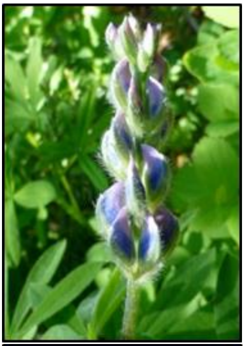

Budding:
- Small purple buds
- Early stages have hairy sepal lining the back of the bud

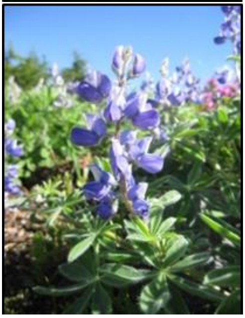

Flowering:
- The flowers are blue to blue-violet and occasionally have white centers
- Each inflorescence has multiple rows (1-6) of 5 flowers encircling the stem

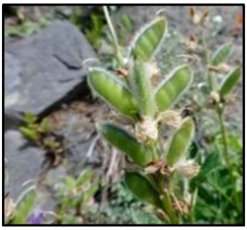

Ripening Fruit:
- Green pods that look like beans replace the flowers
- They are fuzzy and develop to brown pods ~ 1 inch (3cm) long

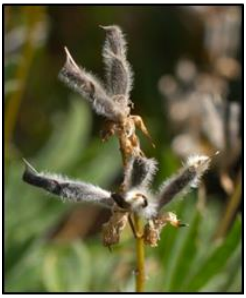

Releasing Seed:
- Once brown, the pods split open, and each half curls back onto itself

****NOTE**** It is common for individual *Lupine* stalks to have multiple phenophases – make careful observations & be sure to consider which phenophases are present and absent.

Western Anemone
Anemone occidentalis

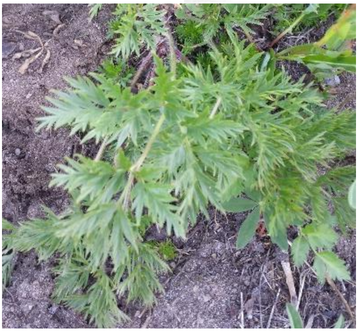

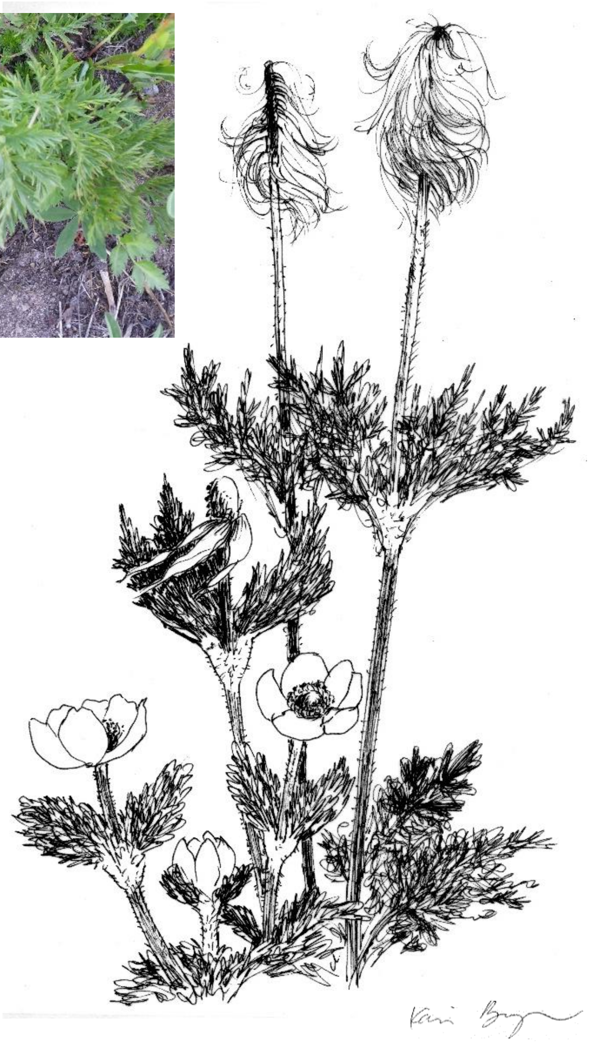

Plant and Leaves:

- Plant is clothed with shiny, silvery hairs, grows continuously throughout the season (up to 24 inches, 60 cm tall)
- Leaves basal and on stem, 4-8 inches (10-20 cm) long, feathery-looking, deeply divided into linear segments

Western Anemone

Anemone occidentalis

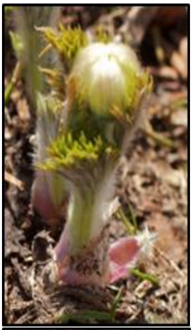

Budding:

- When budding, this is a small plant with a fuzzy, bulbous top
- This is one of the first species to emerge in spring with buds often close to snow

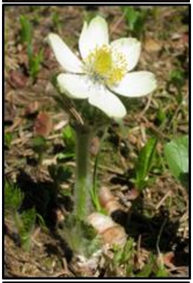

Flowering:

- 6-7 petal-like white sepals with many yellow stamens and many yellow-green pistils in the center
- Flower head is helioptropic, meaning it follows the sun – sometimes you can see insects warming themselves on the flower

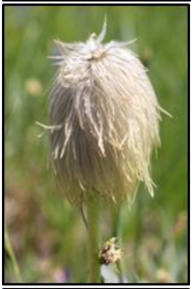

Ripening Fruit:

- The plant looks like a “mop top” or “mouse on a stick” (or a truffula tree from Dr. Seuss!)
- Once the petals fall off, each “hair” starts small at 0.4 in(~1cm) and develops to nearly 2 inches (5 cm)
- Each “hair” of the mop has one seed at the bottom

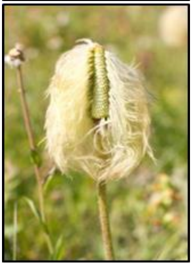

Releasing Seed:

- When the fruits/seeds or “hairs” start to fall off, the plant is releasing its’ seeds.
- When this occurs, the bare yellow stalk shows below the “hair”

Identification Tips

- American Bistort (*P. bistortoides*), pictured on the left, can often be confused with Showy Sedge (*Carex spectabilis*), on the right. Showy Sedge has thin, long grass-like/sedge leaves while American Bistort has larger wider leaves that are dark green with a white midvein. Showy Sedge has smaller white flowers that tend to only be 'showy' on the lower half of the inflorescence (note, showy part are actually male anthers!), while American Bistort tends to flower synchronously within a stalk.

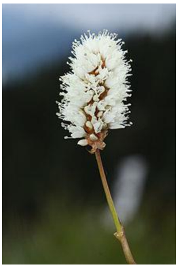

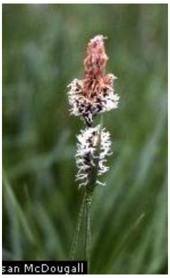

- Mountain Daisy (*E. peregrinus*), left, can often be confused with Cascade Aster (*A. ledophyllus*), on the right. Mountain Daisy has shorter, more densely packed, and numerous ray flowers (these are what look like the purple flower petals) than Cascade Aster. Mountain Daisy has more basal leaves, which are also wider than Cascade Aster leaves. Plots generally do not have both species (although this is variable).

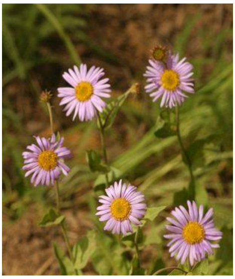

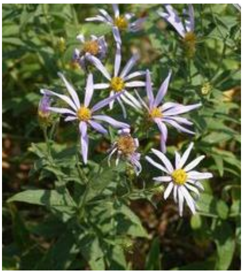

- The fruit and seed phenophases of Mountain Daisy (*E. peregrinus*) and North Microseris (*M. alpestris*) can look similar at first glance. The fruit and seed on Mountain Daisy are more condensed than North Microseris, which are more loosely packed. The foliage is also quite different (see species descriptions on previous pages for pictures).

Species occurrence across plots

	1	2	3	4	5	6	7	8	9	10	11
American Bistort			✓		✓	✓	✓	✓	✓	✓	
Avalanche Lily	✓			✓	✓	✓	✓				
Bracted Lousewort		✓	✓	✓	✓		✓	✓			
Gray’s Lovage	✓		✓	✓		✓	✓	✓			
Magenta Paintbrush		✓		✓	✓	✓	✓	✓		✓	✓
Mountain Daisy	✓						✓	✓	✓		✓
North Microseris						✓		✓	✓		✓
Sitka Valerian			✓	✓		✓	✓		✓		
Subalpine Lupine		✓	✓		✓	✓	✓	✓			✓
Western Anemone								✓	✓	✓	✓

Glossary

Anther – A sack where pollen is stored; found at the end of the stamen filament

Basal – Positioned at the base; “basal leaves” are a cluster of leaves at the base of the stem

Bracts – A specialized leaf, usually at the base of the flower, usually green but can be colored

Calyx – All the sepals of a flower

Compound Leaf – A single leaf that is made up of many smaller leaflets; for example, one lupine leaf is made up of 5-7 leaflets

Corolla – All the petals of a flower

Disc Flower – The central flowers of a flower in the sunflower family; for example, on a daisy each of the small yellow things is a single flower

Filament –The stalk of a stamen that holds the anther

Flower – The reproductive part of the plant; houses the male and female parts

Inflorescence – A cluster of flowers

Lanceolate – Shaped like a blade of a spear; used most often to describe a leaf shape

Oblanceolate – A shape that is opposite of lanceolate, where the widest part is at the top; used most often to describe a leaf shape

Oblong – An elongated, rectangular shape

Obovate – Egg-shaped, the widest part above the middle

Ovary – The expanded basal part of the pistil, containing the ovules

Ovate – Egg-shaped, the widest part below the middle

Ovule – An unfertilized fruit

Petal – An individual part of the corolla, generally colored

Pistil – The female part of the flower, consisting of an ovary, a style, and a stigma

Ray flower – Outer, showy flower of a flower in the sunflower family; each “petal” is actually a flower!

Sepal – An individual part of the calyx, usually greenish and underneath the petals

Sheath – A thin tissue at the base of a leaf that encircles, or partially encircles the stem

Stamen – The male part of the flower, usually a stalk-like filament with a pollen-producing anther on top

Step – When walking normally, each time either foot hits the ground is one step. For example, Left, Right, Left, Right is counted as 4 steps.

Stigma – The portion of the stigma where pollen lands to start the fertilization process

Style – The slender stalk that connects the stigma to the ovary

Umbel – An inflorescence in which multiple flower stalks radiate from a common point

NOTES

Thank you!

We hope that you enjoyed your hike and we appreciate your help in discovering how climate and climate change influence the wildflowers of Mt. Rainier National Park.

Please don't forget to return your data sheet!
Drop it off in the brown metal MeadoWatch box on the porch of building L-112 at Longmire or mail it to us at:

MeadoWatch
University of Washington - Department of Biology
Box 351800, Life Sciences Building
Seattle, WA 98195-1800

Questions? Comments?

MeadoWatch (mwatch@uw.edu); Janneke Hille Ris Lambers (jhrl@uw.edu); Meera Sethi (meeralee@uw.edu)

All photos taken by Natasha Lozanoff or the HRL lab.
Hand drawn wildflowers by Kari Berger.

This material is based upon work supported by the National Science Foundation under Grant Number NSF DEB-1054012 and NASA NNX14AC34G (Applied Sciences Division). Other sponsors (current and past) include the Department of Biology, the National Park Service at Mt. Rainier, Rite-in-the-Rain, Washington's National Park Fund, the Doug and Maggie Walker Endowed Professorship of Natural History, and 113 donors who contributed to our crowdfunding campaign.

Any opinions, findings, and conclusions or recommendations expressed in this material are those of the author(s) and do not necessarily reflect the views of these agencies.

2019
